# Stimuli‐Responsive Silsesquioxane Nanozymes for Organocatalysis in Water and Prodrug Activation in Cells

**DOI:** 10.1002/anie.8446184

**Published:** 2026-04-06

**Authors:** Rabia Zahid, Ariadna Lázaro, Guillermo Moreno‐Alcántar, María Sancho‐Albero, Pierre Picchetti

**Affiliations:** ^1^ Institute of Nanotechnology (INT) Karlsruhe Institute of Technology (KIT) Karlsruhe Germany; ^2^ Institute of Functional Interfaces (IFG) Karlsruhe Institute of Technology (KIT) Karlsruhe Germany; ^3^ Center For Cooperative Research in Biomaterials (CIC biomaGUNE) Basque Research and Technology Alliance (BRTA) Donostia‐San Sebastian Spain; ^4^ Instituto De Nanociencia y Materiales De Argaón (INMA) CISC‐Universidad De Zaragoza Zaragoza Spain; ^5^ Department of Chemical and Environmental Engineering University of Zaragoza Zaragoza Spain; ^6^ Networking Research Center in Biomaterials Bioengineering and Nanomedicine (CIBER‐BBN) Instituto De Salud Carlos III Madrid Spain; ^7^ Instituto De Investigación Sanitaria De Aragón (IISA) Zaragoza Spain

**Keywords:** sol–gel, nanozyme, organocatalysis, enzyme‐mimic, stimuli‐responsive

## Abstract

Synthetic nanozymes have emerged as promising alternatives to natural enzymes for catalytic and therapeutic applications, yet their limited stability, aqueous compatibility, and catalytic scope impede broader utilization. Here, we report a mild, one‐step sol–gel synthesis that yields ultrasmall, water‐stable octa‐amino silsesquioxanes functioning as metal‐free nanozymes. These minimalistic nanostructures exhibit aldolase‐like organocatalytic activity in water and enable dynamic, stimuli‐responsive modulation of catalysis through reversible supramolecular aggregation and disaggregation triggered by specific chemical inputs, thus forming a multifunctional platform for tunable catalysis and biomedical applications. Structural simplicity, stability, and functional versatility together permit tunable, enzyme‐like catalysis in water without auxiliary surfactants or phase‐transfer additives. Furthermore, the nanozymes display high biocompatibility and efficient cellular internalization, enabling their use in living cells, for instance, as intracellular prodrug activators via retro‐aldol activation of a doxorubicin prodrug in human glioblastoma and metastatic melanoma cells, resulting in selective cytotoxicity. This system provides a cost‐effective, sustainable, and scalable platform for water‐compatible, metal‐free organocatalysis that bridges abiotic catalysis and biological function. These findings demonstrate how rationally designed silsesquioxane frameworks can emulate natural enzyme reactivity while integrating adaptive, stimuli‐responsive behavior, broadening the applicability of synthetic nanozymes to catalytic and therapeutic contexts.

## Graphical abstract

Amino‐bearing silsesquioxanes are water‐stable nanozymes that exhibit aggregation‐ and disaggregation‐dependent catalysis and can be applied to intracellular prodrug activation for cancer therapy.

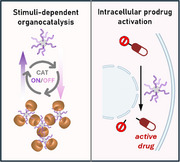

## 1 Introduction

The development of synthetic materials that mimic the catalytic properties of natural enzymes, such as synthetic macrocycles [1, 2, 3, 4, 5, 6, 7, 8, 9] metal‐complexes [10, 11], nanoparticles (nanozymes) [12, 13, 14, 15], has emerged as an important strategy to overcome the limitations of catalysis in industry and to enable next‐generation therapeutic approaches [16], including prodrug‐activation‐based therapies [17, 18]. Nevertheless, there remains a need for nanomaterials that exhibit enhanced stability in water and biological media and can be produced cost‐effectively at scale for both biological and industrial applications. A wide range of nanosystems functionalized with metal complexes [12, 19, 20, 21], as well as engineered nanomaterials [22, 23, 24, 25, 26, 27, 28, 29, 30, 31, 32, 33, 34, 35, 36] including metallacages [37, 38, 39], have been proposed as next‐generation platforms for green catalysis and potentially safter nanomedicines. In catalysis, there is a critical need for water‐compatible organocatalytic nanomaterials that operate in purely aqueous media, are synthetically scalable, and can transform challenging, poorly water‐soluble substrates under sustainable conditions [40, 41]. In the context of catalysis that is relevant to biomedical applications, metal complexes [42, 43] and metal‐based nanoparticles [28, 30, 44] are available, but there is also a clear need to expand concepts toward organocatalytic materials that are synthetically scalable, water‐stable, and biocompatible. Metal‐free options, for example in the design of prodrug activators, are therefore highly desirable as prodrug strategies are increasingly viewed as a paradigm shift for improving treatment safety and efficacy, particularly in oncology [44, 45, 46, 47, 48].

Silsesquioxanes [49] are an attractive class of hybrid organic–inorganic materials consisting of a rigid siloxane core bearing well‐defined organic pendants, which can be readily functionalized. Traditionally used as building blocks for coatings and fillers [50, 51], they have more recently found roles in biomedical applications as additives in hydrogels or fibers [52]. However, their exploitation as nanozymes, especially in fully aqueous systems, has been limited by stability issues, and the development of mild, sustainable routes to functional, water‐stable derivatives, as well as their application in organocatalysis and biological contexts, remains largely underexplored. For instance, organocatalytic transformations such as the lysine‐catalyzed aldol reaction in aldolases remain largely unexplored in nanozyme‐based biomedical applications, with only a handful of examples in other contexts [53, 54, 55], and a few early precedents involving catalytic antibodies [56, 57, 58]. Mimicking such systems with silsesquioxanes could provide valuable alternatives to antibodies, which are often limited by low intracellular abundance and by the immunogenicity associated with exogenous administration [59, 60], and to metal‐based catalysts, whose speciation, fate, and off‐target toxicity in biological systems are difficult to control [61]. In addition, functionalization possibilities of the organic groups of silsesquioxanes could be exploited to introduce also stimuli‐responsive organocatalysis, for example, mimicking the dynamic aggregation and disaggregation of enzymes in cells to tune their activity [62, 63, 64].

In this work, we report the design and efficient, mild, one‐step sol–gel synthesis of a series of amino‐modified, ultrasmall, water‐stable silsesquioxane nanoparticles with a octahedral architecture. The incorporation of amino groups endows these materials with enzyme‐like properties by enabling straightforward supramolecular organocatalysis in water in a reversible, stimuli‐responsive manner via controllable supramolecular aggregation and disaggregation. Furthermore, showcasing their broad applicability also in a biological context, we demonstrate that these octa‐amino silsesquioxanes are biocompatible and can mediate the activation of a doxorubicin prodrug in two cancer cell models (glioblastoma and melanoma), leading to efficient cytotoxicity activation. Overall, our findings establish silsesquioxanes as a versatile and powerful class of colloidal nanozymes, bridging biomimetic catalysis with clinically relevant prodrug‐activation applications.

## 2 Results and Discussion

### 2.1 Synthesis of Silsesquioxane‐Based Nanozymes

**FIGURE 1:**
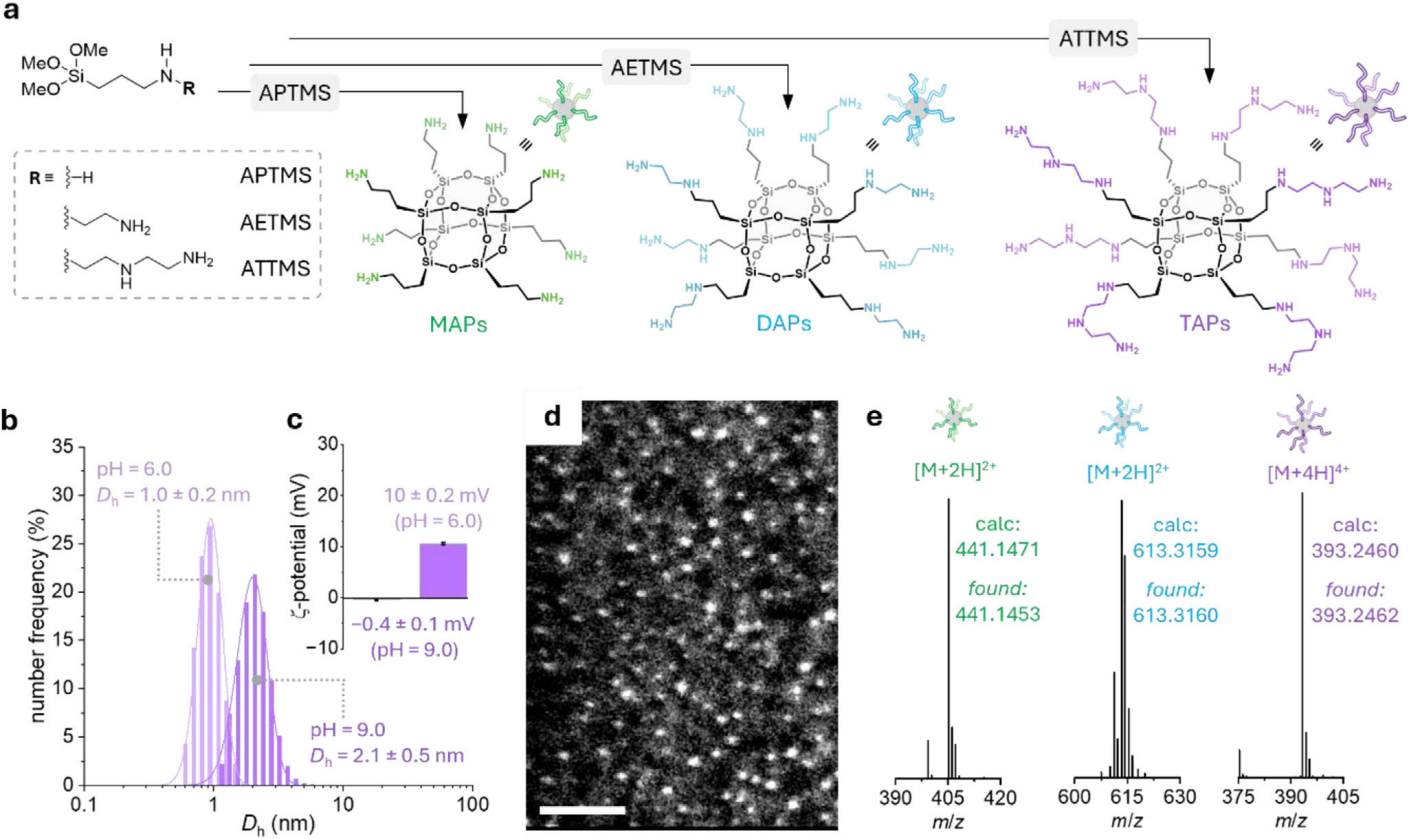
(a) Schematic representation of the use of amino‐functionalized trimethoxysilanes for the preparation of octahedral silsesquioxanes: MAPs, DAPs, and TAPs using APTMS, AETMS, and ATTMS, respectively. (b) DLS analysis of TAPs (10 mg/mL in 10 mM ammonium buffer) at pH 6.0 and pH 9.0. (c) *ϛ*‐potential analysis of TAPs (10 mg/mL in 10 mM ammonium buffer) at pH 6.0 and pH 9.0. (d) STEM image of TAPs; scale bar = 20 nm. (e) HR‐ESI‐MS analysis of MAPs, DAPs, and TAPs.

The synthesis of octahedral silsesquioxanes was carried out via controlled hydrolysis and condensation of amino‐bearing trialkoxyorganosilanes (see Supporting Information), by modifying a previously reported protocol for pure silica nanoparticles [65]. Specifically, a series of amino‐bearing trialkoxyorganosilanes, (3‐aminopropyl)trimethoxysilane (APTMS), N‐(2‐aminoethyl)‐3‐aminopropyltrimethoxysilane (AETMS), and 3‐[(trimethoxysilyl)propyl]diethylenetriamine (ATTMS) were employed to test the feasibility of integrating catalytically active amino functionalities into the final particles. These precursors were selected to introduce organic side chains bearing monoamino, diamino, or triamino groups, respectively, leading to the formation of nanoparticles hereafter referred to as MAPs, DAPs, and TAPs (Figure 1a).

Interestingly, although we initially expected the formation of larger particles (∼20 nm), similar to those reported when tetraethyl orthosilicate (TEOS) was used in the original procedure, our results differed in both composition and morphology. We first observed that dynamic light scattering (DLS) analysis of the resulting colloidal dispersion revealed the presence of ultrasmall nanoparticles, with hydrodynamic diameters (*D*_h_) at pH 9.0 of 1.7 ± 0.3 nm for MAPs, 2.5 ± 0.4 nm for DAPs, and 2.1 ± 0.5 nm for TAPs (Figures 1b and S1). Upon adjusting the pH to 6.0, the *D*_h_ of the particles decreased to 1.0 ± 0.3, 1.0 ± 0.4, and 1.0 ± 0.2 nm for MAPs, DAPs, and TAPs (Figures 1b and S1), respectively. This size reduction can be attributed to particle disaggregation at acidic pH, where protonation‐induced electrostatic repulsion outweighs interchain hydrogen bonding and dispersion interactions. This was further confirmed by ζ‐potential (ζ‐pot) measurements (see Figures 1c and S1 inset), which showed that the particles transition from an almost neutral charge state to a positively charged state upon acidification of colloidal dispersion. In addition, scanning transmission electron microscopy (STEM, Figure 1d) as well as TEM images of TAPs (Figure S2a,b) showed nanoparticles with diameters of 1–2 nm (Figure S2c), and energy‐dispersive X‐ray spectroscopy (EDS, Figure S2d) confirmed the presence of silicon within the particles. When evaluating their long‐term stability, no colloidal disintegration or notable aggregation was observed for at least 1 month when storing them as synthesized TAPs at 4°C (Figure S3).

To gain deeper insight into the structure of the synthesized particles, nuclear magnetic resonance (NMR), attenuated total reflectance‐Fourier tansform infrared (ATR‐FTIR) spectroscopy, and mass spectrometry (MS) were used, confirming their cage‐like octa‐amino silsesquioxane framework. First, ATR‐FTIR analysis (Figure S4a) shows that all three samples exhibit two characteristic Si─O stretching bands at ∼1100 cm^−1^, indicative of cyclic Si─O─Si structures [66], suggesting cage‐like octasilsesquioxane structures. Additionally, the C─H vibrational bands at ∼2300 and ∼1450 cm^−1^, corresponding to organic residues in the nanostructures, follow an intensity trend where increasing organic chain length reduces the influence of the Si atom on terminal C─H vibrations, thereby enhancing relative band intensity. All samples show the characteristic vibrational band corresponding to non‐protonated amines (R─NH_2_ and R─NH─R) in the 3500–3200 cm^−1^ region and of protonated ammonium groups in the 2800–2300 cm^−1^ region (Figure S4b) [67], with only DAP and TAP showing bands attributable to R─NH_2_^+^─R at ∼2800 and ∼1660 cm^−1^ [68].

Confirmation of the prevalence of an octa‐amino silsesquioxane structure with a cage‐like framework with eight silicon vertices, was also obtained via ^1^H NMR (see Figures S5, S7, S9). The spectra show the typical broadened molecular peaks with the expected integrations of the aliphatic proton resonance signals occurring at 0.5 ppm (Si─C*H*_2_─), 1.6 ppm (C─C*H*_2_─C) and 2.6 ppm (─NH─C*H*_2_─ and ─C*H*_2_─NH_2_) [69, 70, 71, 72]. Further evidence was obtained by ^13^C NMR spectra (Figures S6, S8, S10), which show and match the expected and reported carbon signals of MAPs, DAPs, and TAPs, as well as high‐resolution electrospray ionizazion mass spectrometry (HR‐ESI‐MS) analysis (see Figures 1e and S11). The formation of octa‐amino silsesquioxanes in our synthetic methodology can be rationalized by the fact that trialkoxyorganosilanes contain nonhydrolysable Si─C bonds, which limit crosslinking and network growth by reducing the extent of Si─O─Si bond formation. Methoxy substituents were selected due to their faster hydrolysis rates compared to bulkier alkoxy groups [73, 74, 75]. The reactions were carried out in a biphasic cyclohexane–water system, where the organoalkoxysilane gradually diffused into the aqueous phase (10 mM ammonium buffer, pH 9.0), undergoing rapid polycondensation and ultimately leading to the formation of octahedral silsesquioxanes.

Compared to previously reported syntheses, this procedure enables the preparation of three amino‐functionalized polysilsesquioxanes, including the previously unreported TAP derivative, in good yields (>30%) within 12 h, under mild conditions and without the use of strong acids, extended reaction times (often exceeding one week), or additional purification and drying steps [72, 76, 77]. In addition, the overall yields of the isolated and freeze‐dried particles were 1.11 g for MAPs (60%), 1.26 g for DAPs (37%), and 1.60 g for TAPs (32%), respectively. These results demonstrate the good efficiency of the synthesis methodology, enabling their large‐scale preparation. To evaluate the broader applicability of the method, we also examined other trimethoxy‐ and triethoxy‐substituted organoalkoxysilanes (Figure S12), which, however, only led to the formation of large (µm‐sized) amorphous precipitates, highlighting both the importance of methoxy substituents on the silicon center and the compatibility of amines in the synthesis.

### 2.2 Biomimetic and Stimuli‐Responsive Catalytic Features of Octa‐amino Silsesquioxanes

Responsiveness to allosteric activators and competitive inhibitors is a characteristic of natural enzymes desirable to be encoded in enzyme‐mimetic systems. An important objective is the development of nanozymes that mimic aldolases in lysine‐like aldol and retro‐aldol reactions in water, whose activity can be reversibly regulated through supramolecular host–guest interactions. In this context, we envisioned TAPs as attractive nanozymes capable of mediating the transformation of water‐insoluble substrates in aqueous media without relying on supplementary surfactants [40, 78], because their confined aliphatic triamines create a lipophilic water–silsesquioxane core interface at which catalysis can occur. Moreover, we demonstrate that TAPs activity can be up‐ and downregulated on demand in a biomimetic fashion.

**FIGURE 2:**
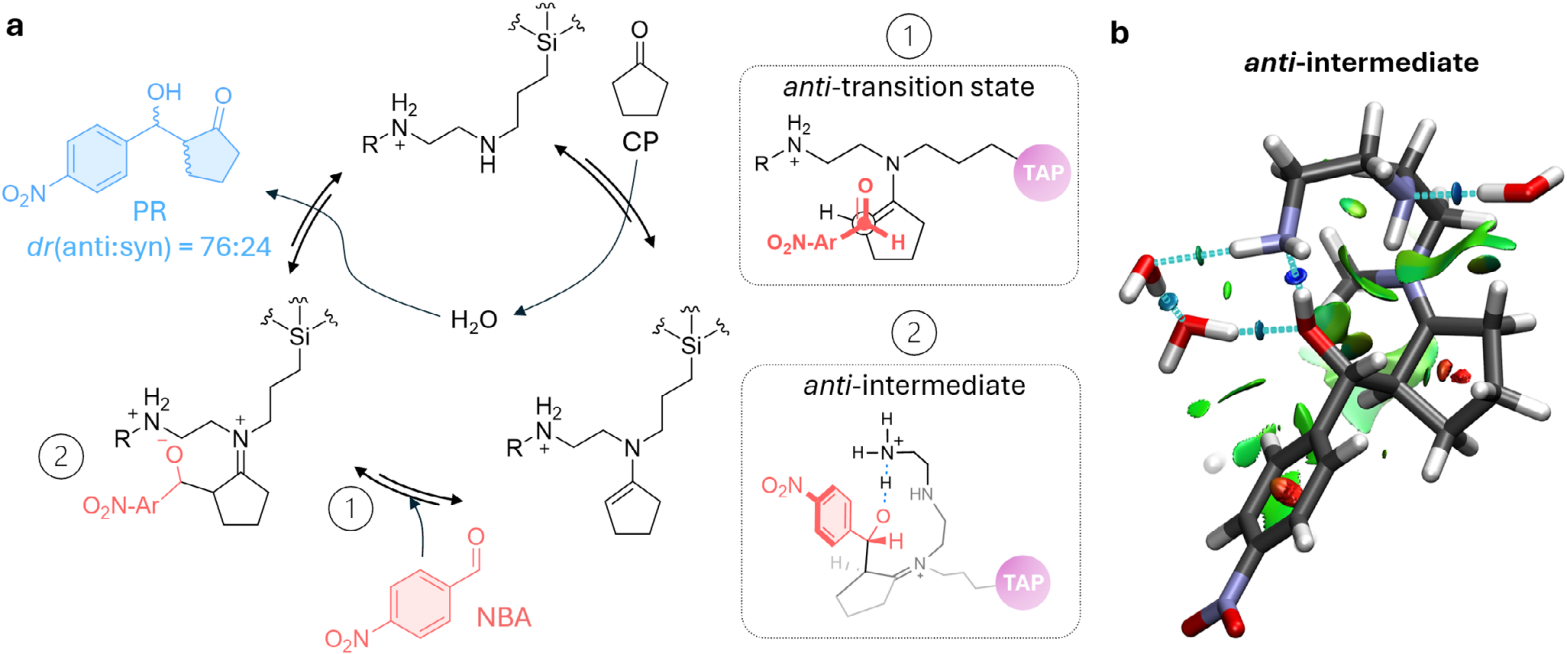
(a) Proposed mechanism of the aldol reaction between NBA and CP in the presence of TAPs (shown as truncated structures). Shown are the plausible spatial arrangements of the preferred transition state (1) and the intermediate (2), respectively, which account for the formation of the predominant antidiastereomer. (b) Optimized structure (PBE0‐D4/def2‐TZVPP/CPCM) of the anti‐intermediate, highlighting attractive H‐bonding regions (blue and cyan), attractive van der Waals contacts (green) and repulsive regions (red), which together contribute to the overall antistabilization in TAPs.

To assess the catalytic performance of TAPs, we investigated their ability to promote the aldol addition between 4‐nitrobenzaldehyde (NBA) and cyclopentanone (CP) in water at pH 7 (Figure 2; see Supporting Information), without any performance enhancers such as organic cosolvents, polymers, or micellization agents [79, 80, 81, 82, 83, 84, 85], a challenging setting for most catalysts (vide infra) that directly showcases the intrinsic efficiency of our nanozymes. Screening revealed that TAPs (6 mol%) afforded the aldol product (PR) in 20% isolated yield with a diastereomeric ratio (*anti*:*syn*) of 77:23 (Figure S13). No product was observed in the absence of TAPs (Figure S14). From a mechanistic perspective, the pronounced antiselectivity observed with cyclopentanone [83], together with density‐functional theory (DFT) calculations (see Supporting Information and Figure 2b and Figures S15,S16), supports a Zimmerman–Traxler‐type transition state in which an anti‐conformation minimizes steric repulsion between the aldehyde and both the silsesquioxane core and the cyclopentane ring (Figure 2). This transition state and the corresponding intermediate are further stabilized through several N⋯H⋯O H‐bonds, as also revealed by DFT calculations (Figure 2b) [86, 87, 88]. In contrast, these type of interactions are less favorable in the *syn*‐configured intermediary (Figure S16). Notably, the TAPs provide not only a suitable lipophilic interface but also a favorable hydrogen‐bonding network, thereby lowering the activation energies and closely resemble an enzyme‐like pocket, for which confinement plays an essential role.

Compared to available reports with free amines that require around 30 mol% catalyst [89], TAPs display markedly higher efficiency in water (see Table S1), highlighting their applicability and strong performance, especially given that cosolvents such as DMSO, micellization agents, polymers, or even water‐miscible starting materials are typically required to boost performance. This superior performance can be attributed to two key features. First, confinement of the amines around the ∼1.2 nm silsesquioxane core lowers their protonation tendency relative to nonconfined analogues, as seen for polyamine polymers [90, 91], thereby maintaining a significant fraction of catalytically active free amines. Second, the aliphatic nature and reduced protonation of the confined polyamines create a hydrophobic interlayer between the siloxane core and bulk water, which promotes substrate accumulation and provides a confined reaction space for catalysis [92]. Taken together with the observed diastereoselectivity in water, which proves the relevance of hydrogen‐bonding in stabilizing the intermediary states, these results support a cooperative mechanism in which surface amine chains preorganize NBA and CP, where one amine forms the enamine with the ketone, while neighboring protonated amines hydrogen‐bond to the nitro group or aldehyde oxygen to promote C─C bond formation (Figure 2).

**FIGURE 3:**
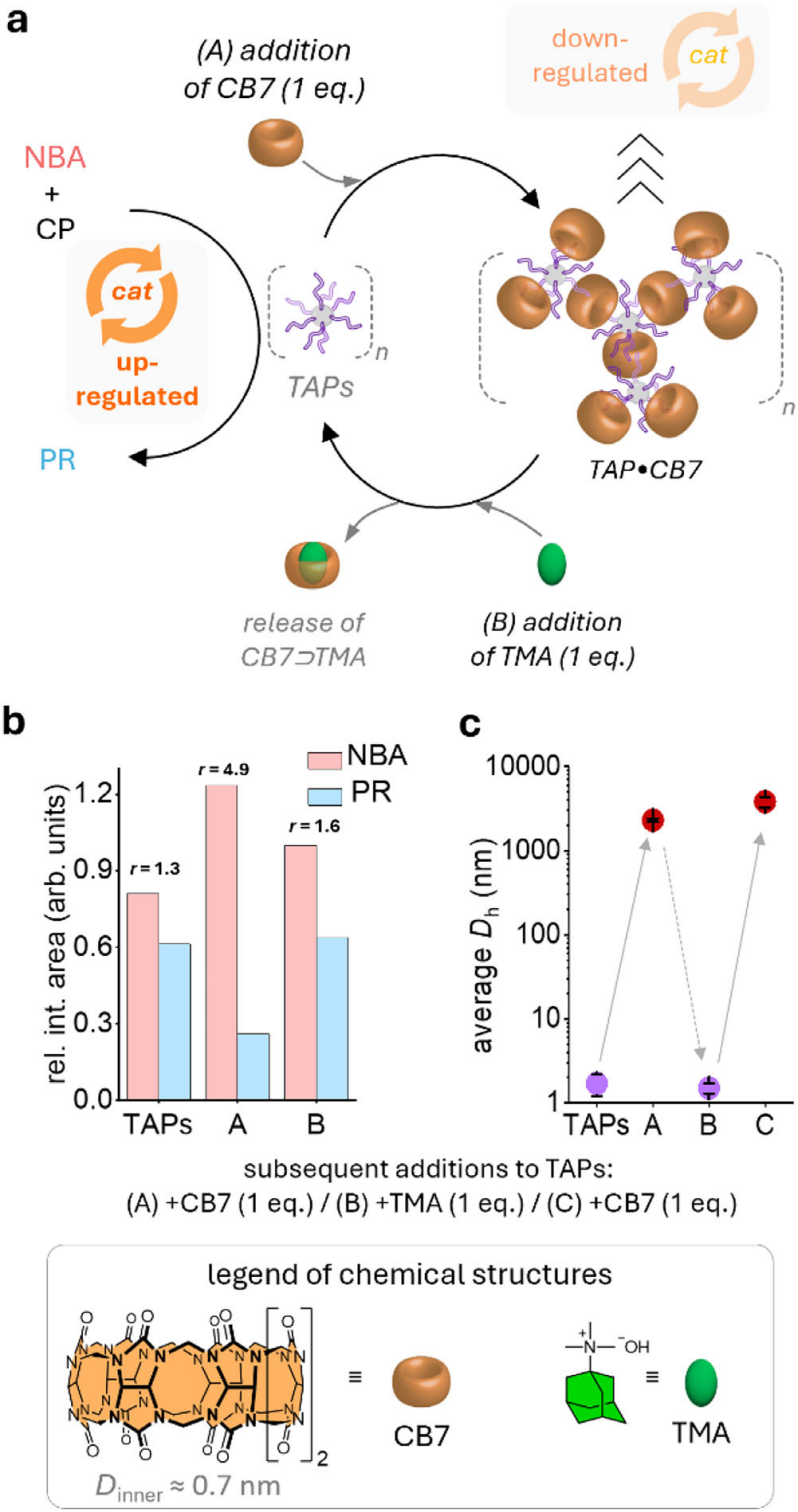
(a) Schematic representation of the supramolecularly controlled up‐ and downregulation of the TAP‐catalyzed aldol reaction. (b) Relative NMR integration areas of NBA and PR, along with the corresponding *r* value, calculated as the ratio between the integration areas of NBA and PR. (c) Aggregation and disaggregation of TAPs, triggered by the sequential addition of CB7 (1 equiv.) and TMA (1 equiv.).

Having established that TAPs efficiently catalyze the aldol reaction, we investigated whether their activity could be regulated in a biomimetic manner. We aimed to mimic up‐ and downregulation of catalysis via controlled aggregation, inspired by enzyme regulation through liquid–liquid phase separation–like processes [62, 63, 64]. In this respect, TAPs offer the required organic pendants that resemble the polyamine spermidine (SPE), a strong binder for cucurbituril (CB7, log *K*_a_ ≈ 8.7 for spermidine). [93] Thus, CB7 acts as an inhibitor that can reversibly crosslink TAPs, while a competitive guest can externally control the aggregation state and, consequently, catalysis (Figures 3 and S17). To implement this concept, we used CB7 as a supramolecular crosslinker to generate micrometer‐sized TAP•CB7 aggregates, and *N*,*N*,*N*‐trimethyl‐1‐adamantylammonium hydroxide (TMA, log *K*_a,CB7 _ =  12.2) [94] as a high‐affinity competitor that disassembles these aggregates by forming the more stable CB7⊃TMA complex. We anticipated that aggregated and disaggregated TAPs would display markedly different efficiencies in catalyzing the aldol reaction (Figure 3a).

To evaluate the effect of aggregation, we compared the normalized integration ratios of NBA and PR relative to an internal standard (*r*‐value), based on their aromatic proton signals (see SI). NMR studies revealed that catalysis was downregulated upon addition of CB7 (6.1 µmol, *V*_tot_ = 1 mL), which induced supramolecular crosslinking and the formation of micrometer‐sized TAP•CB7 aggregates. In this aggregated state, the *r*‐value (NBA/PR) increased from 1.3 (free TAPs) to 4.9, indicating reduced catalytic activity and accumulation of unreacted NBA (Figures 3b and S18). The reduced activity can be attributed to TAP crosslinking, as evidenced by DLS (Figure 3c, see also Figure S19) and TEM analyses (Figure S20), which confirmed the formation of micrometer‐sized aggregates, also visible by visual inspection (Figure S21). These aggregates limit the surface area available for catalysis and decrease substrate diffusivity toward the reactive amino sites. Catalysis was restored upon addition of TMA (1 equiv.), which triggered the disassembly of TAP•CB7 aggregates into free TAPs (Figures 3a and S19) via formation of the more stable CB7⊃TMA complex. The *r*‐value changed to 1.6, comparable to that observed for unaggregated TAPs, indicating catalytic upregulation as TAPs were released and dispersed. Control experiments in the absence of TAPs yielded only trace amounts of PR (Figure S22).

### 2.3 Biological Properties of TAPs and Their Application as Prodrug Activators

Beyond establishing a new nanozyme for stimuli‐responsive, biomimetic organocatalysis in water, we aimed to harness the intricate architecture of TAPs to showcase its versatility for biomedical applications, particularly prodrug activation. Existing organocatalytic amine‐containing antibodies [95, 96] often suffer from suboptimal performance and immunogenicity, [59, 60] underscoring the need for synthetic, nonprotein, water‐stable catalysts that function efficiently in complex biological environments, including inside cells.

**FIGURE 4:**
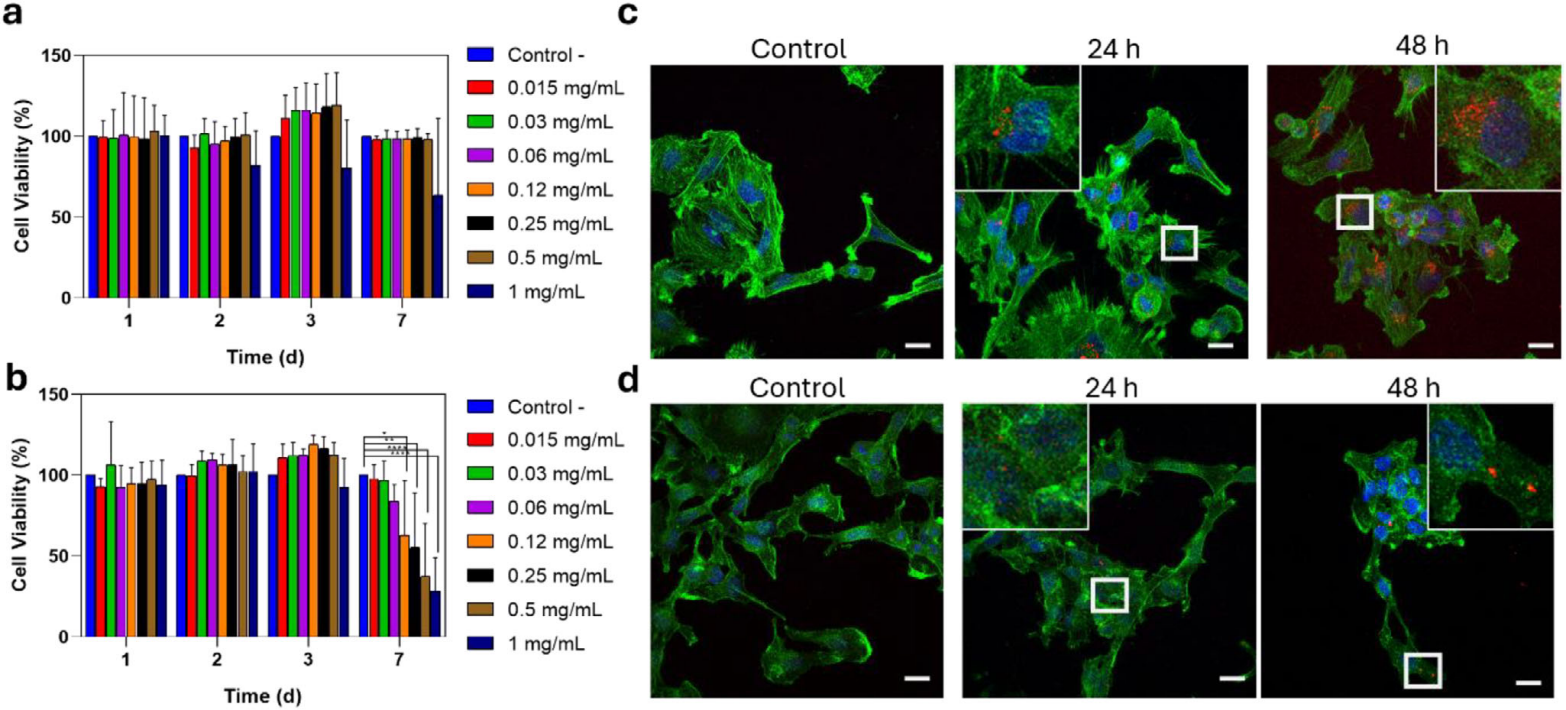
Biocompatibility study of TAPs on (a) U251‐MG cells and (b) B16‐F10 cells during 1, 2, 3, and 7 days. Confocal images of (c) U251‐MG cells and (d) B16‐F10 cells incubated with TAPs during 24 and 48 h (dose = 1 mg·mL^−^^1^). Scale bars: 20 µm. Cy5‐TAPs are shown in red, nuclei in blue and actin filaments in green.

Before evaluating TAPs for intracellular prodrug applications, we first assessed their biocompatibility in human U251‐MG glioblastoma cells (a clinically relevant model that offers valuable translational insights) and melanoma B16‐F10 cells (a high metastasis murine model). Cytotoxicity tests showed that TAPs exhibited high biocompatibility in both cell lines (Figure 4a,b), even at high concentrations (1 mg·mL^−^^1^) over 7 days of incubation. Results indicated that both cell cultures did not exhibit any statistically significant decrease on cell viability compared to control (nontreated) cells after 1, 2, and 3 days of incubation at any tested concentration (from 0.015 to 1 mg·mL^−1^). Only the highest concentrations of TAPs decreased cell viability of B16‐F10 cells after 7 days of incubation. TAPs were fully compatible at any tested time‐points and concentrations in the case of U251‐MG cells. Also, the biocompatibility of TAPs was evaluated against healthy cells (both human and murine; Figure S23). In particular, the potential cytotoxic effects were tested against human fibroblasts, human placental mesenchymal stem cells (hpMSCs), NIH‐3T3 cells (murine fibroblasts) and murine mesenchymal stem cells derived from bone marrow (mMSCs). The results demonstrate the high biocompatibility of TAPs also against the tested healthy cells. Indeed, only at the highest doses and the largest incubation time‐point (1 week) lead to a reduction of cell viability compared to control nontreated cells.

Further the cell‐uptake in two cell lines (see Supporting Information) was confirmed by Z‐stacks obtained by confocal microscopy using cyanine5‐labelled TAPs (Cy5‐TAPs, 1 mg·mL^−1^) during 24 and 48 h (Figure 4c,d and Figures S24,S25). Confocal‐laser scanning microscopy analysis clearly revelated the presence of red pixels covering the cytoplasmic region, corresponding to aggregated TAPs within the cells and demonstrating their successful internalization in U251‐MG and B16‐F10 cells.

**FIGURE 5:**
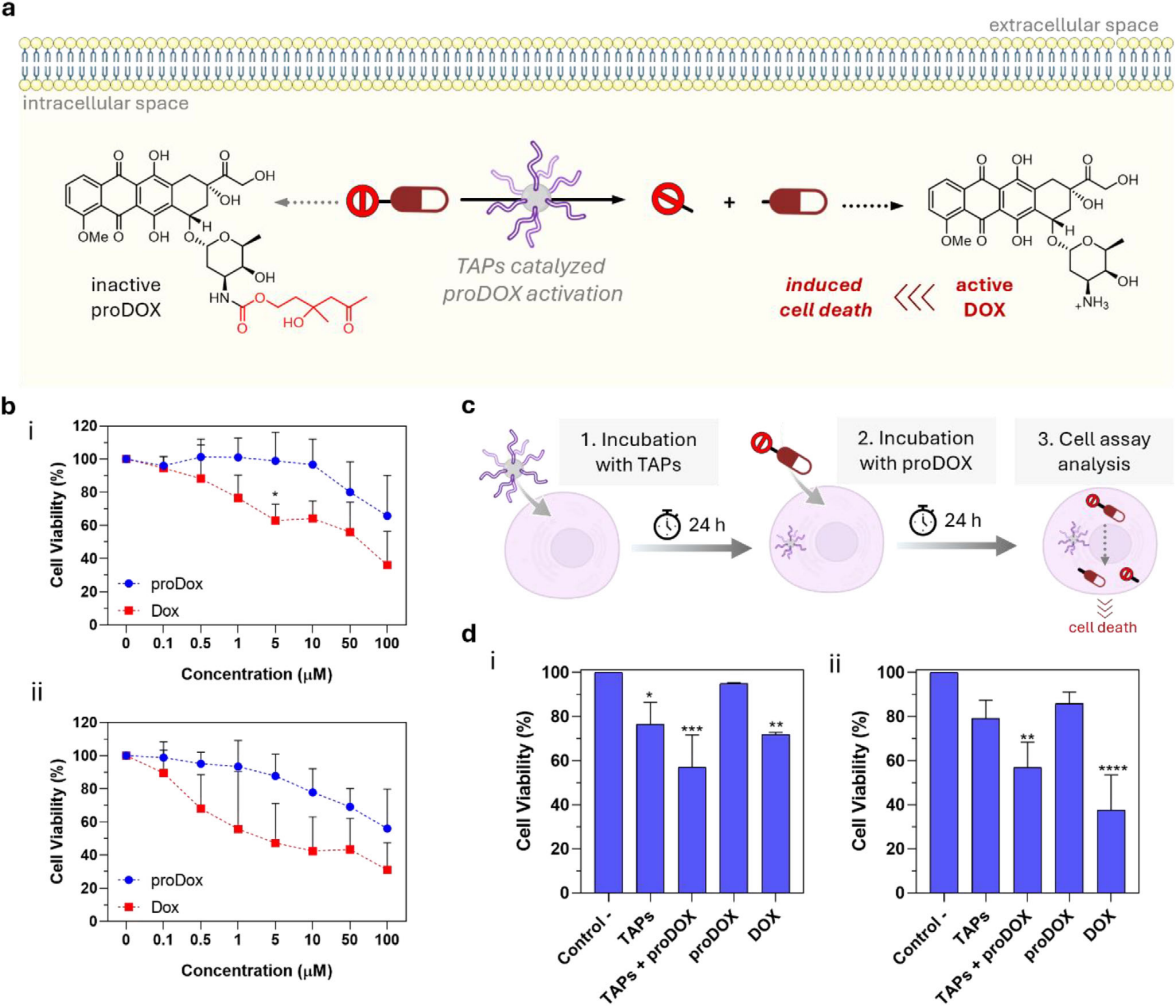
(a) Schematic representation of intracellular proDOX activation by TAPs. The inactive proDOX is converted by the catalytic activity of TAPs into DOX. The released DOX subsequently induces cell death through multiple modes of action. (b) Dose‐response curves after 24 h of treatment with proDOX and DOX in U251‐MG (i) and B16‐F10 cells (ii). (c) Scheme of proDOX intracellular activation incubation steps: incubation of cells with TAPs (0.3 mg·mL^−^^1^) during 24 h, followed by incubation with the proDOX (5 µM) for further 24 h. (d) Cell viability of U251‐MG cells (i) and B16‐F10 cells (ii). As a negative control, cells alone or treated just with TAPs or with proDOX separately were used; as a positive control, commercially available DOX (5 µM) was applied.

With this, we next aimed to exploit the nanozyme capabilities of TAPs for activating caged drugs in cells. Inspired by early work on catalytic antibodies, TAPs were used to uncage a β‐hydroxyketone–doxorubicin (DOX) derivative (proDOX, see Suppoorting Information for its synthesis) [95], in this caged form the DNA intercalation and topoisomerase II inhibition properties of DOX are largely suppressed (Figure 5a). TAPs catalyze the aldol formation between an aldehyde and a ketone and, by Le Châtelier's principle, should also promote the corresponding retro‐aldol reaction, enabling activation of caged proDOX, as previously shown with antibodies. [95, 96]

To prove this, we first confirmed that TAPs promote the retro‐aldol cleavage of a model β‐hydroxyketone (4‐(6‐(dimethylamino)naphthalen‐2‐yl)‐4‐hydroxybutan‐2‐one, DANO, see Supporting Information for its synthesis) [97]. This reaction can be monitored by luminescence (Figure S26), as DANO is weakly emissive, whereas its retro‐aldol product 6‐(dimethylamino)‐2‐naphthaldehyde (DAA) is strongly luminescent, providing a convenient readout of catalytic activity (Figure S27a). When TAPs were mixed with an aqueous solution of DANO, an increase in fluorescence intensity (116%) was observed, consistent with the formation of DAA, whereas in the absence of TAPs only a minor background increase (≈20%) was detected, confirming that they promote the retro‐aldol reaction (Figure S27) [98]. A pH study (Figure S28) further showed that this retro‐aldol activity is maximized under basic conditions and essentially absent under acidic conditions, consistent with pH‐dependent protonation of the polyamine sites on TAPs.

Once the retro‐aldol reaction–mediating capabilities of TAPs were confirmed, we proceeded to test their ability to uncage proDOX in living cells. We first evaluate the cytotoxicity of proDOX and DOX alone. Cells were treated for 24 h with DOX and proDOX at concentrations ranging from 100 to 0.1 µM and cell viability was determined. Comparison between the dose response curves of DOX and proDOX evidenced that the caged compound displays a significant reduction of the toxicity compared with DOX in both cell lines (U251‐MG cells above and B16‐F10 cells below, Figure 5b). In these experiments, proDOX induced little toxicity at *c* = 5.0 µM (∼99% and ∼88% cell viability for U251‐MG and B16‐F10 cells, respectively), whereas DOX at the same concentration decrease their cell viability to ∼63% and ∼47% for U251‐MG and B16‐F10 cells, respectively and in concordance with previous reports. Therefore, 5.0 µM was chosen as an ideal concentration of proDOX for the prodrug activation experiments [95].

For the intracellular proDOX activation, a nontoxic dose of TAPs (0.3 mg·mL^−1^) was used to limit particle‐induced toxicity. U251‐MG and B16‐F10 cells were incubated with TAPs during 24 h to allow their internalization. Afterward, the noninternalized particles were washed away (to prevent extracellular proDOX uncaging). The cells were then treated with proDOX and incubated during additional 24 h to allow its internalization and intracellular uncaging by TAPs. Subsequently, the cell viability of the treated cells was determined (Figure 5c). To ensure validation, three negative controls were included, namely U251‐MG cells treated with TAPs only, B16‐F10 cells treated with TAPs only, and cells incubated with proDOX alone. As a positive control, cells were treated with DOX, and cell viability was assessed in all conditions (see Supporting Information and Figure 5d). Our results showed that, when compared to untreated cells, U251‐MG cells treated separately either with TAPs or proDOX alone maintained good cell viability (76% and 95%, respectively). On the contrary, when cells were exposed to TAPs in combination with proDOX they exhibited a drastic decrease in viability (57%) which was comparable to the viability observed with DOX tratment (72%). Similarly, B16‐F10 cells, maintained high viability when separately treated either with TAPs or proDOX (79% and 86%) which was decreased to 57% when they were with TAPs in combination with proDOX, while the treatment with DOX resulted in a significantly lower viability (37%). Overall, these results confirm the ability of TAP nanozymes to mediate the intracellular uncaging of proDOX to DOX.

## 3 Conclusion

With the aim of providing new nanomaterials capable of complex, stimuli‐responsive tasks across a broad range of applications, this work introduces octa‐amino silsesquioxanes as ultrasmall, water‐stable nanozymes. We establish a scalable and mild one‐step bottom‐up sol–gel procedure on the gram scale, providing a cost‐effective route to these materials. Their enzyme‐like activity is regulated through reversible supramolecular aggregation and disaggregation, closely mimicking cofactor‐regulated enzyme function and offering new strategies to tune organocatalysis in water. To support their use in biological settings, we demonstrate good biocompatibility in both healthy and cancer cells, cellular uptake, and, importantly, the ability to catalyze intracellular prodrug activation in human glioblastoma and metastatic melanoma cells. Together, these results establish octa‐amino silsesquioxanes as promising nanozymes for catalysis under challenging water‐based conditions without performance enhancers, and for biomedical applications, as exemplified by a first proof‐of‐concept prodrug activation study. Using a β‐hydroxyketone‐caged doxorubicin as an inactive prodrug model, TAPs activate the prodrug inside cells, restoring its cytotoxic activity with an efficacy comparable to that of commercial doxorubicin. Compared to previously reported catalytic antibodies, TAPs offer clear advantages: (i) efficient and scalable synthesis via alkoxysilane chemistry, (ii) lower immunogenicity due to their non‐proteinogenic nature, (iii) a broader substrate scope and (iv) the ability to reach effective intracellular concentrations that are otherwise difficult to achieve with enzymes. Overall, by merging cost‐effective synthesis, biomimetic catalysis, and therapeutic function, TAPs establish a platform for the next generation of synthetic nanozymes that can potentially bypass the limitations of protein‐based systems. Moreover, the described stimuli‐responsive features represent important findings with implications not only for catalysis but also for the design of synthetic cells.

## Conflicts of Interest

The authors declare no conflicts of interest.

## Supporting information




**Supporting File 1**: Details about instruments, materials and methods, synthesis, characterization, analysis, and bioexperiments can be found in the Supporting Information. The authors have cited additional references within the Supporting Information [55, 86, 87, 89, 92, 95, 96, 97, 99, 100, 101, 102, 103, 104, 105, 106, 107, 108, 109].

## Data Availability

The data that supports the main findings of this study are available in the Supporting Information of this article as well as from the corresponding author upon reasonable request. The authors have cited additional references within the Supporting Information.
