# Supplementary material for: Stimuli‐Responsive Silsesquioxane Nanozymes for Organocatalysis in Water and Prodrug Activation in Cells
Source: Angew Chem Int Ed Engl. 2026 Apr 6;65(21):e8446184. doi: 10.1002/anie.8446184 (PMC13182218; doi:10.1002/anie.8446184)
Supplement: Supplementary file 1 — Supporting File 1: Details about instruments, materials and methods, synthesis, characterization, analysis, and bioexperiments can be found in the Supporting Information. The authors have cited additional references within the Supporting Information [55, 86, 87, 89, 92, 95, 96, 97, 99, 100, 101, 102, 103, 104, 105, 106, 107, 108, 109]. [file ANIE-65-e8446184-s001.docx]

**Supporting Information**

**Stimuli-Responsive Silsesquioxane Nanozymes for Organocatalysis in Water and Prodrug Activation in Cells**

Rabia Zahid,^1^ Ariadna Lázaro,^2^ Guillermo Moreno-Alcántar,^3^ María Sancho-Albero,^4,5,6,7^* Pierre Picchetti^1^*

^1^ Institute of Nanotechnology (INT) Karlsruhe Institute of Technology (KIT), Kaiserstrasse 12, 76131 Karlsruhe, Germany

^2^ Institute of Functional Interfaces (IFG), Karlsruhe Institute of Technology (KIT), Kaiserstrasse 12, 76131 Karlsruhe, Germany

^3^ Center for Cooperative Research in Biomaterials (CIC biomaGUNE), Basque Research and Technology Alliance (BRTA), 20014 Donostia-San Sebastian, Spain

^4^Instituto de Nanociencia y Materiales de Argaón (INMA), CISC-Universidad de Zaragoza, Campus Rio Ebro, Edificio I+D, C/ Poeta Mariano Esquillor, s/n, Zaragoza, 50018, Spain.

^5^Department of Chemical and Environmental Engineering University of Zaragoza, Spain, Campus Rio Ebro, C/ María de Luna, 3, Zaragoza, 50018, Spain.

^6^Networking Research Center in Biomaterials, Bioengineering and Nanomedicine (CIBER-BBN), Instituto de Salud Carlos III, Madrid, 28019, Spain.

^7^Instituto de Investigación Sanitaria de Aragón (IISA), Avenida San Juan Bosco, 13, Zaragoza, 50009, Spain.

E-Mail: [msancho@unizar.es](mailto:msancho@unizar.es), [pierre.picchetti@kit.edu](mailto:pierre.picchetti@kit.edu)

**Table of Contents**

[1. Abbreviations 3](#_Toc226124343)

[2. Instruments 4](#_Toc226124344)

[Attenuated total reflectance Fourier transform infrared spectroscopy 4](#_Toc226124345)

[Dynamic light scattering 4](#_Toc226124346)

[Electrospray ionization mass spectrometry 4](#_Toc226124347)

[Luminescence spectroscopy 4](#_Toc226124348)

[Nuclear magnetic resonance (NMR) spectroscopy 4](#_Toc226124349)

[pH measurements 4](#_Toc226124350)

[UV-Vis absorption spectroscopy 4](#_Toc226124351)

[3. Experimental details 5](#_Toc226124352)

[General information 5](#_Toc226124353)

[General synthesis of MAPs, DAPS and TAPs 5](#_Toc226124354)

[Synthesis of cyanine5-labelled TAPs 6](#_Toc226124355)

[pH-dependent DLS analysis 6](#_Toc226124356)

[pH titrations 6](#_Toc226124357)

[CB7- and TMA-dependent aggregation of TAP 6](#_Toc226124358)

[General procedure for performing TAPs-catalyzed aldol reactions in the absence of CB7 or TMA 6](#_Toc226124359)

[General procedure for performing aggregation-dependent TAPs catalysis 6](#_Toc226124360)

[General procedure for the TAPs-catalyzed retro-aldol reaction 7](#_Toc226124361)

[Synthesis of DANO 7](#_Toc226124362)

[Synthesis of proDOX 7](#_Toc226124363)

[Cell cultures. 8](#_Toc226124364)

[Biocompatibility studies. 8](#_Toc226124365)

[Intracellular proDOX activation 9](#_Toc226124366)

[Statistical analysis. 9](#_Toc226124367)

[Computational details 9](#_Toc226124368)

[4. Supporting figures 10](#_Toc226124369)

[6. Supporting tables 37](#_Toc226124370)

[7. Supporting references 40](#_Toc226124371)

## Abbreviations

AETMS N-(2-Aminoethyl)-3-aminopropyltrimethoxysilane

APTMS (3-Aminopropyl)trimethoxysilane

ATR Attenuated total reflectance

ATTMS 3-[(Trimethoxysilyl)propyl]diethylenetriamine

CB7 Cucurbit[7]uril

CDCl_3_ Deuterated chloroform

CP Cyclopentanone

CPCM Conductor-like polarizable continuum model

Cy5 Cyanine5

D_2_O Deuterium oxide

DAA 6-(dimethylamino)-2-naphthaldehyde

DANO 4-(6-(dimethylamino)naphthalen-2-yl)-4-hydroxybutan-2-one

DAP Di-amino nanoparticles

*D*_h_ Hydrodynamic diameter

DLS Dynamic light scattering

DOX Doxorubicin

EDX Energy-dispersive X-ray

eq. equivalents

ESI Electrospray ionization

EtOAc Ethyl acetate

FNA 6-(Dimethylamino)-2-naphthaldehyde

FTIR Fourier transform infrared spectroscopy

GOAT Global optimization algorithm for molecules and atomic clusters

k_b_ Boltzmann constant

LEV Levulinic acid

MAP Mono-amino nanoparticles

MS Mass spectrometry

MWCO Molecular weight cut-off

NBA 4-Nitrobenzaldehyde

NCI Non-covalent interaction

NMR Nuclear magnetic resonance

PBS Phosphate buffered saline

PR Aldol reaction product

proDOX Doxorubicin prodrug

rpm revolutions per minute

STEM Scanning transmission electron microscopy

TAP Triamino-nanoparticles

TEM Transmission electron microscopy

TMA *N,N,N*-trimethyl-1-adamantylammonium hydroxide

U-251MG human malignant glioblastoma multiforme cell line

VMD Visual molecular dynamics

*η* Viscosity

θ Scattering angle

## Instruments

Attenuated total reflectance Fourier transform infrared spectroscopy. IR spectra were recorded on a Thermo Scientific Nicolet iS50 spectrometer. Samples were measured in the wavelength range from 4000 cm^−1^ to 500 cm^−1^ upon direct deposition of the pure compounds.

Dynamic light scattering. Measurements were performed using a Malvern ZetaSizer Nano instrument. The intensity of the scattered light was measured at a fixed angle (173°). The wavelength of the laser light used for the light scattering experiments was 633 nm. Data analysis was performed according to standard procedures using the Malvern software. Briefly, the decay rates were determined by the following relationship, where *η* is the viscosity of the medium, *D*_h_ is the hydrodynamic diameter, and θ is the scattering angle.

$$\Gamma=D_{h}\cdot\left( \frac{4\pi\eta}{\lambda} \cdot\sin\left( \frac{\theta}{2} \right) \right)^{2}$$

The method of cumulants was used to fit the autocorrelation function, which in turn allows the determination of the diffusion coefficient (*d*), from which the hydrodynamic diameter (*D*_h_) of the aggregates is calculated using the Stokes-Einstein equation (see below), where k_b_ is the Boltzmann constant, *T* is the temperature, and *η* is the viscosity of the medium.

$$D_{h}=\frac{k_{b}\cdot T}{3\cdot\pi\cdot\eta\cdot d}$$

Electrospray ionization mass spectrometry. Electrospray-ionization (ESI) and high-resolution (HR) ESI mass spectrum were recorded on an LTQ-FT Ultra mass spectrometer (Thermo Fischer Scientific). Spray voltage: 3.6 kV; capillary temperature: 300 °C; capillary voltage: –12; tube lens voltage: –86.7; sheath gas: 45; sweep gas: 0; auxiliary gas: 40. For organic molecules, acetonitrile (ACN) was used as the solvent, and a diluted solution of MAPs, DAPs, or TAPs in Milli-Q water was used for their analysis.

Luminescence spectroscopy. To record emission spectra a Horiba Jobin−Yvon IBH FL-322 Fluorolog 3 spectrometer equipped with a 450 W xenon arc lamp, double grating excitation, emission monochromators (2.1 nm/mm of dispersion; 1200 grooves/mm) and a TBX-04 single photocounting detector was used. Quartz cuvettes with a light path of 10 mm and dimensions of 10 × 10 mm with a spectroscopic cut-off at 220 nm were utilized.

Nuclear magnetic resonance (NMR) spectroscopy. ^1^H and ^13^C NMR spectra were recorded in CDCl_3_ or D_2_O on a Bruker Advance 500 spectrometer at 25 °C. The chemical shifts (*δ*) are given in ppm and refer to residual protons on the corresponding deuterated solvent. The Mestrelab Research analytical chemistry software was used to analyze the NMR data.

pH measurements. The pH of the solutions, waere measured using a Mettler Toledo FiveEasy Plus pH meter when preparing TAPs solutions or dilutions of water-soluble reagents such as CB7 or TMA. In cases where catalysis was performed in which suspensions were present, the pH was monitored using universal indicator pH paper strips.

UV-Vis absorption spectroscopy. Absorbance spectra were measured at 25 °C in Milli-Q water on a Agilent Carry 5000 UV-Vis/NIR spectrophotometer. Quartz cuvettes with a light path of 10 mm and dimensions of 10 × 10 mm with a spectroscopic cut-off at 220 nm were utilized for UV-Vis absorption experiments.

**Confocal microscopy**. TAPs internalization was assessed by confocal microscopy (Spectral Confocal Microscope Zeiss LSM 880 with Airyscan) with a 63x oil immersed N.A. 1.40 objective. Z-stack orthogonal projections were performed to determine the presence of NPs inside the cytosol.

**TEM microscopy**. Imaging and elemental analysis of TAPs was assessed with a Tecnai F30 (Thermo Scientific, formerly FEI) operated at 300 kV in STEM (Scanning-Transmission Electron Microscopy) mode: High Angle Annular Dark Field (STEM-HAADF) images were obtained with a HAADF detector (Fischione) and, in order to analyze the chemical composition of the materials, X-ray Energy Dispersive Spectra (EDS) were obtained with an EDAX detector. A suspension of TAPs was pipetted onto a TEM copper grid with a holey carbon film. Electron microscopy analysis was carried out in the Advanced Microscopy Laboratory (LMA).

**Data Availability**. The primary research data underlying this study are openly available in Radar4Chem at doi: 10.35097/7x2kkpq9a6qtxg98

## Experimental details

General information. Water at Milli-Q purity was used for all experiments (0.055 µS/cm at 25°C; provided by the arium®pro purification system). All organic solvents were from commercial sources and used without further purification. (3-Aminopropyl)triethoxysilane (≥98%), (3-aminopropyl)trimethoxysilane (97%), vinyltrimethoxysilane (98%), (3-chloropropyl)trimethoxysilane, carboxyethylsilanetriol sodium salt (25% in water), 4-nitrobenzaldehye (≥98%), lithium diisopropylamide solution (1.0 M in THF/hexane), triethylamine (≥99% GC), anhydrous sodium sulfate, phosphate buffered saline (tablet, pH 7.2-7.6) and deuterated solvents were bought from Merck. 2-(4-Pyridylethyl)triethoxysilane (97%) was purchased by Gelest. Cyclopentanone (99%) was purchased by Alfa Aesar. Cucurbit[7]uril (95%), 6-(dimethylamino)-2-naphthaldehyde (95%), doxorubicin hydrochloride (99%) and *N,N,N*-trimethyl-1-adamantylammonium hydroxide (25% in water) were purchased by BLDpharm). 3-[2-(2-Aminoethylamino)-ethylamino]-propyltrimethoxysilane (85%), N-(2-aminoethyl)-3-aminopropyltrimethoxysilane (97%), (3-aminopropyl)trimethoxysilane (96%) and 6-(dimethylamino)-2-naphthaldehyde (95%) were purchased from ABCR Chemie. Levulinic acid (≥97%) was purchased from TCI. Cyanine5 NHS ester was purchased from Lumiprobe. Hydrochloric acid (37% in water) was purchased by from VWR. Dialysis membrane Spectra/Por® Biotech CE MWCO 100–500, 16 mm was purchased from Carl Roth.

The PBS buffer was prepared by dissolving one tablet in the specified amount of MilliQ water, yielding a 0.01 M phosphate buffer containing 0.0027 M potassium chloride and 0.137 M sodium chloride, pH 7.4, at 25 °C. The ammonium buffer (10 mM) was prepared by dissolving the appropriate amount of ammonium chloride in Milli-Q water, followed by pH adjustment to 9.3 using a diluted ammonia solution in Milli-Q water.

For reagents used in biology-related experiments, see section “Cell cultures.”

General synthesis of MAPs, DAPS and TAPs. In a 25 mL round-bottom flask equipped with a Teflon coated magnetic stirring bar, ammonium buffer (10 mL, 10 mM, pH 9.3) was added and heated under stirring until the solution reached 60 °C. The stirring speed was then increased to 900 rpm, and a solution of the organotrialkoxysilanes (10.9 mmol) in cyclohexane (1.43 mL) was rapidly injected into the stirring buffer solution using a cannula. It should be noted that vigorous stirring is essential to form a turbid, opaque oil-in-water emulsion.

Following the injection, the reaction mixture was sealed and allowed to stir at 60 °C for 12 h. Subsequently, the mixture was cooled to room temperature, and phase separation was achieved by gravity. The aqueous layer, containing the final particles, was collected using a syringe.

Yields were determined by lyophilizing the resulting aqueous solutions and weighing the recovered material. An equal volume of ammonium buffer was lyophilized separately to account for the minimal contribution of salt from the buffer. The yield was corrected accordingly.

To adjust the pH of the particle dispersions, diluted hydrochloric acid was added until the desired pH (6.0 or 7.0) was achieved. For dilution to the desired particle concentration during measurements or reactions, Milli-Q grade water was used, resulting in a change in the buffer concentration.

Synthesis of cyanine5-labelled TAPs. To a solution of TAPs in Milli-Q water (61.0 µmol) at pH 7.0 (*V*_tot_ = 3.2 mL), Cy5-NHS ester (0.18 mg, 0.17 µmol) was added and allowed to stir overnight. The reaction mixture was subsequently dialyzed against water (MWCO = 500 Da) for 12 h.

pH-dependent DLS analysis. A particle concentration of 100 mg·mL⁻¹ was used to measure the pH-dependent size distribution. To prepare the samples for DLS analysis, the as-synthesized nanoparticles were diluted in Milli-Q water to achieve the desired concentrations. The pH was then adjusted using diluted HCl (in Milli-Q water) to reach either pH 9.0 or pH 6.0.

pH titrations. Potentiometric acid–base titrations were carried out at 25 °C using a combined glass electrode connected to an automatic titrator. Samples were dispersed in water containing NaCl (0.10 M) to ensure constant ionic strength.

CB7- and TMA-dependent aggregation of TAP. Three separate solutions were prepared: 1) TAPs (6.1 µmol), 2) TAPs (6.1 µmol) with CB7 (1 equivalent), and 3) TAPs (6.1 µmol) with CB7 and a subsequent addition of TMA, all at equimolar ratios. Each solution was prepared to a final volume of 1 mL.

General procedure for performing TAPs-catalyzed aldol reactions in the absence of CB7 or TMA. In a glass vial (6 mL total volume, diameter 1.5 cm) equipped with a stirring bar, NBA (15.0 mg, 0.1 mmol) was added. Subsequently, Milli-Q water (500 µL, pH 7) was added to the vial containing NBA, followed by the addition of a colloidal suspension of TAPs (previously adjusted to pH 7; 6.1 µmol), CP (51.0 µL, 0.5 mmol), and finally, water to adjust the total volume to 1 mL. The resulting suspension was stirred at 300 rpm at room temperature for 12 hours in the dark. Thereafter, EtOAc (6 mL) was added, and the stirring speed was increased to 1000 rpm to extract the organic material from the aqueous phase over a period of 30 minutes. Stirring was then stopped, and the two phases were allowed to separate. Five milliliters of the organic phase were collected in a separate vial and dried over a minimal amount of Na₂SO₄. After the addition of EtOAc (1 mL), 5 mL of the organic phase were collected again, and the solvent was evaporated under reduced pressure to obtain the crude reaction mixture. The reaction product was isolated by column chromatography (silica gel, EtOAc/hexane = 2:8). After solvent evaporation, the aldol product was obtained as a white solid.

Regarding the amount of TAPs used for the isolation of the aldol product, 6.1 µmol was employed. While lower amounts of TAPs led to detectable product formation, only the use of 6.1 µmol of TAPs over 12 hours resulted in quantities sufficient for isolation. Under these conditions, the aldol product PR was obtained in a yield of 20%.

General procedure for performing aggregation-dependent TAPs catalysis. To study the aggregation-dependent catalytic behavior of TAPs, either a colloidal mixture of TAP (3.0 µmol) and CB7 (1:1 molar ratio), or alternatively, a mixture containing TAPs, CB7, and TMA (1:1:1 molar ratio), was prepared by adding CB7 or both CB7 and TMA to a colloidal dispersion of TAPs at pH 7. The resulting solutions or suspensions (in the case where TAPs were mixed with CB7) were allowed to stand under static conditions for at least 15 minutes prior to their addition to the NBA–water suspension, as described above. Note that in the case where TMA was included, the solution was likewise used at pH 7. The subsequent work-up was carried out as previously described. The residual was redissolved in CDCl_3_ (0.5 mL) to which 1,4-dioxane (0.5 µL) was added as an internal standard.

The catalytic up- or downregulation was evaluated by integrating one pair of aromatic signals from either NBA (the proton signal in the ortho position relative to the nitro group) or the aldol product PR (the proton signal in the meta position relative to the nitro group), and normalizing them to the integral value of the 1,4-dioxane signal. The intensities used for calculations are reported in Table S2. Mestrelab Research analytical chemistry software was used for this purpose. To obtain a more comparable quantitative measure between these calculated integrals, the following equation was applied:

$$r_{NBA/PR}=\left( \frac{I_{norm}\left( NBA \right)}{I_{norm}(PR)} \right)$$

General procedure for the TAPs-catalyzed retro-aldol reaction. In a plastic cuvette equipped with a teflon-coated stirring bar, DANO (3.13 µmol, in 10mM PBS at pH 7.0, *V*_tot_ = 2.5 mL) and TAPs (5 eq.) were added from diluted aqueous (Milli-Q water grade) stock solutions at pH 7.0. The reaction mixture was then stirred in the dark. Over time, luminescence spectra were recorded to monitor the formation of the aldol product, which exhibits increased emissivity, by measuring the emission from the cuvette (*λ*_ex_ = 308 nm). The data were normalized by using:

$$\Delta F/F_{0}(t)=\frac{F(t)-F_{0}}{F_{0}}$$

where *F*_0_ is the fluorescence at *t* = 0 and Δ*F* = *F*(t) − F_0_ represents the absolute fluorescence change relative to baseline.

The resulting data were fitted with an exponential growth function in Origin to provide a visual guide.

Synthesis of DANO. 4-(6-(dimethylamino)naphthalen-2-yl)-4-hydroxybutan-2-one (DANO) was synthesized by adapting a previously reported procedure.^1^ Briefly, under an argon atmosphere, to a solution of LDA (1.2 mmol in 5 mL of dry THF) at –78 °C, acetone (1.2 mmol) was added dropwise. The resulting mixture was stirred for 30 minutes. Subsequently, a solution of 6-(dimethylamino)-2-naphthaldehyde (0.5 mmol in 1 mL of dry THF) was added dropwise at –78 °C. The reaction mixture was stirred for an additional 15 minutes, followed by the dropwise addition of a saturated aqueous solution of ammonium chloride (500 µL). The reaction was then allowed to warm to room temperature under vigorous stirring. The mixture was extracted with diethyl ether (3 × 5 mL), and the combined organic phases were dried over anhydrous Na₂SO₄, filtered, and the organic solvent was evaporated under reduced pressure. The crude product was further purified by column chromatography (silica gel, EtOAc/hexane = 1:1), giving DANO as a yellow solid (20 mg, 15%). ^1^H NMR (CDCl_3_, 400 MHz): *δ* 7.70-7.64 (m, 3H), 7.36 (m, 1H), 7.18 (m, 1H), 6.91 (m, 1H), 5.25 (m, 1H), 3.28 (br s, 1H), 3.04 (s, 6H), 2.93-2.84 (m, 2H), 2.20 (s, 3H) ppm. The ¹H NMR spectrum matches the one reported in the literature (see Fig. S29).^2^

Synthesis of proDOX. The synthesis of a functionalized doxorubicin with a β-hydroxyketone group was performed as reported in the literature. For the functionalization**,** 3-hydroxy-3-methyl-5-oxohexyl (4-nitrophenyl) carbonate was prepared and reacted with Doxorubicin hydrochloride. This linker was obtained by the same synthetic route described in the literature with identical characterization.^3, 4^ Doxorubicin hydrochloride (15 mg, 0.026 mmol) was dissolved into 1.5 ml of dimethylformamide and triethylamine (5.25 ml, 0.038 mmol). Subsequently, 11.7 mg (0.038 mmol) of 3-hydroxy-3-methyl-5-oxohexyl (4-nitrophenyl) carbonate were dissolved in a minimal amount of dimethylformamide and added to the solution. The mixture was stirred at room temperature for 16 h and afterwards, the solvent was removed under reduced pressure. The residue was dissolved in ethyl acetate, filtered and purified by silica column chromatography using ethyl acetate:methanol (9:1) as an eluent to give proDOX as a red solid (15.8 mg, 86%). ^1^H NMR (CDCl_3_, 400 MHz): δ 13.97 (s, 1H), 13.24 (s, 1H), 8.03 (d, J = 7.5 Hz, 1H), 7.79 (t, J = 8.1 Hz, 1H), 7.39 (d, J = 8.4 Hz, 1H), 5.50 (d, J = 3.4 Hz, 1H), 5.28 (s, 1H), 5.05 (d, J = 8.5 Hz, 1H), 4.76 (s, 2H), 4.55 (s, 1H), 4.20 – 4.10 (m, 3H), 4.08 (s, 3H), 3.84 (s, 1H), 3.67 (s, 1H), 3.27 (d, J = 18.9 Hz, 1H), 3.07 – 2.87 (m, 2H), 2.62 (q, J = 17.4 Hz, 2H), 2.33 (d, J = 14.7 Hz, 1H), 2.22 – 2.11 (m, 5H), 1.90 – 1.71 (m, 5H), 1.29 (d, J = 6.6 Hz, 3H), 1.21 (s, 3H). ^13^C NMR (101 MHz, CDCl^3^) *δ* 213.98, 210.79, 187.28, 186.86, 161.21, 156.35, 155.80, 135.95, 135.66, 133.73, 121.03, 120.02, 118.60, 111.75, 111.58, 100.84, 70.69, 69.65, 67.41, 65.68, 61.50, 56.83, 52.57, 47.08, 40.48, 35.79, 34.15, 31.92, 30.27, 27.28, 16.99. ESI-MS(+): [M+Na]^+^, 738.2300 (calcd. for C_35_H_41_NO_15_Na,738.2400). The ¹H and ¹³C NMR spectra are shown in Fig. S30‒S31, and the mass spectrum is shown in Fig. S32.

Cell cultures. Glioblastoma cells (U251-MG), melanoma cells (B16-F10), human dermal fibroblasts, murine fibroblasts (NIH-3T3 cells) and murine mesenchymal stem cells derived from bone marrow (mMSCs) were provided by cell services from Cancer Research-UK. They were grown in Dulbecco’s modified Eagle’s medium (DMEM, Teknovas) with 10 % Fetal Bovine Serum (FBS, Thermo Fisher Scientific), supplemented with 1 % penicillin/streptomycin and 1 % amphotericin (Biowest) and 1 % glutamine (Gibco) and maintained at 37 ºC in humidified atmosphere under normoxic conditions. Human placental mesenchymal stem cells (hpMSCs), were obtained from Cellular engineering Technologies (CET (Coraville, IA, USA) and were grown DMEM (Biowest, France) supplemented with 5 μg ml^−1^ of FGF-2 growth factor (PeproTech, USA), with 10% of foetal bovine serum (FBS, GIBCO, USA), 1% penicillin/streptomycin and 1% amphotericin (Biowest, France) and maintained at 37 °C in humidified atmosphere under normoxic conditions. The different biological characterization experiments of TAPs were carried out by the Synthesis of Nanoparticles Unit (UNIT 9) of the ICTS “NANBIOSIS” at the Institute of Nanoscience and Materials of Aragon (INMA)-Universidad de Zaragoza.

Biocompatibility studies. To determine the biocompatibility properties of TAPs, the Blue Cell Viability Assay Kit (Abnova) was employed following the manufacturer recommendations. To perform the assay, U251-MG and B16-F10 cells were seeded at a density of 4 x 10^3^ cells per well (into a 96-well plate) in 100 μL of the previously described cell culture media. After 24 h, the medium was replaced by 100 μL of freshly prepared medium containing the TAPs (0.015, 0.03, 0.06, 0.12, 0.25, 0.5 and 1 mg·mL^−1^) and incubated for another additional 1, 2, 3 and 7 days. At these different time-points cells were washed twice with PBS and treated with the with the Blue Cell Viability Assay Kit (Abnova, 10 % v/v in cell culture media). It is a non-fluorescent resazurin-based chemical that upon reduction by metabolically active and live cells is converted to a highly fluorescent product (resofurin). 100 % of cell viability was attributed to non-treated cells. Fluorescence emission was detected using a plate reader (Ex/Em: 540/590 nm). Experiments were performed in triplicates.

The cellular uptake and internalization of TAPs in U251-MG and B16-F10 cells was evaluated by confocal microscopy. They were seeded at a density of 2.5 x 10^4^ onto 20 mm cover slips (located in a 24-well plate) and incubated under standard culture conditions during 24 h. Then, 1 mg·mL^−1^ of Cy5-TAPs was added and incubated for 24 and 48 h. Afterwards, cells were fixed with 4 % paraformaldehyde for 30 min at RT and stained with Phalloidin-Alexa Fluor 488 (Invitrogen) and ProLong Glass Antifade Mountant with NucBlue Stain (Thermo Fisher Scientific). The cells were imaged by confocal microscopy carrying out Z-stack acquisitions.

**Dose-response study in U251-MG and B16-F10 cells: proDOX *vs* DOX**. Both cell lines were plated as indicated above. The corresponding wells were then replaced with a suspension of proDOX and DOX diluted in cell culture media at 100, 50, 10, 5, 1, 0.5 and 0.1 μM and incubated for 24 hours. Cells were finally washed, and the Blue Cell Viability Assay Kit (Abnova) was carried out as previously mentioned.

Intracellular proDOX activation **by TAPS**. U251-MG and B16-F10 cells were seeded as described before in 96 multi-well plates. Then, cells were treated with TAPs (0.3 mg·mL^−1^) for 24 h allowing their internalization within the cells. Afterwards, they were washed with PBS to remove the non-internalized particles and treated with freshly media containing proDOX (5 μM) for another 24 h. Non-treated cells, cells treated with only TAPs (0.3 mg·mL^−1^) and cells incubated with only proDOX (5 μM) were used as negative control. Also, DOX-treated cells (5 μM) were employed as positive control. Finally, cell viability was determined as described above.

Statistical analysis. Biological experiments were carried out in triplicate. All the data are indicated as mean ± SD. Statistical analysis of the significant differences among the means were analyzed by one-way or two-way analysis of variance (ANOVA) for multiple comparisons (GraphPad Prism Software version 8.0.1). Statistically significant differences are expressed as follows: *p < 0.05; **p < 0.01; ***p < 0.001 and ****p < 0.0001.

Computational details. All calculations were performed using ORCA 6.1.1.^5^ For the DFT calculations, a model system was constructed in which the silsesquioxane core was replaced with a 2,6,7-trioxa-1-silabicyclo[2.2.2]octane moiety bearing a single triamine chain, which serve as a truncated minimal representation of TAPs/NBA/CP reactive intermediary structure. For this model, global conformational searches were conducted with the GOAT^6^ algorithm employing the fast GFN2-xTB^7^ semiempirical method. The lowest-energy conformer from the GOAT ensemble (Δ*G* < 3 kcal/mol cutoff) was subsequently refined via geometry optimization at the dispersion-corrected hybrid PBE0-D4/def2-TZVPP^8, 9^ level within the CPCM continuum model of water (*ε* = 78.39). To explicitly account for microsolvation effects on hydrogen bonding, needed to accurately modelling solvent contributions, water molecules were manually positioned ~1.9–2.2 Å from key N–H···O donor–acceptor sites.^10^ The resulting system was reoptimized under identical methods (PBE0-D4/def2-TZVPP/CPCM. This hybrid explicit–implicit approach captures short-range H-bonding while incorporating bulk dielectric screening, outperforming pure continuum models for flexible H-bond networks. The optimized structures are displayed in Fig. S15 and the atomic coordinates given in Tables S3 and S4. Non-covalent interactions were visualized via NCI web (see Fig. S16),^11, 12^ using the structure’s promolecular densities and visualized in VMD (isosurface RDG = 0.25 au, color scale –0.06 to 0.06 au). This NCI protocol has been extensively validated for H-bonded supramolecular systems.

## Supporting figures


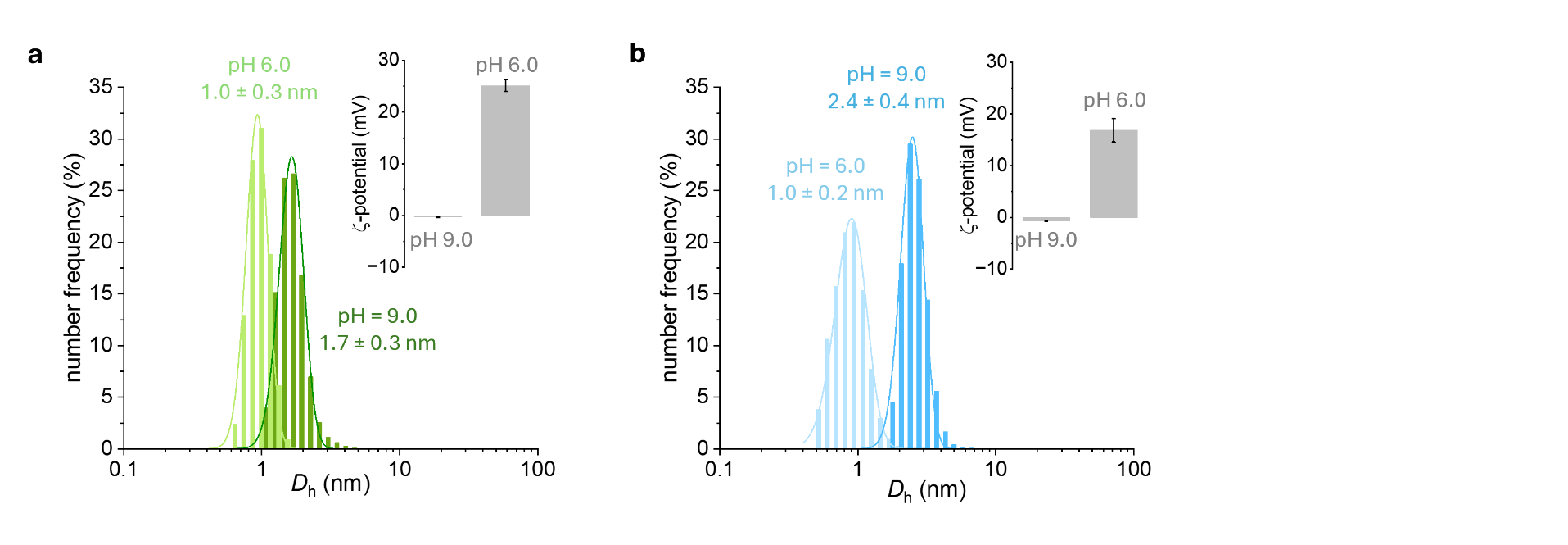


Fig. S 1*.* DLS and ϛ-potential (insets) analysis of (a) MAPs and (b) DAPs at basic and acidic pH.


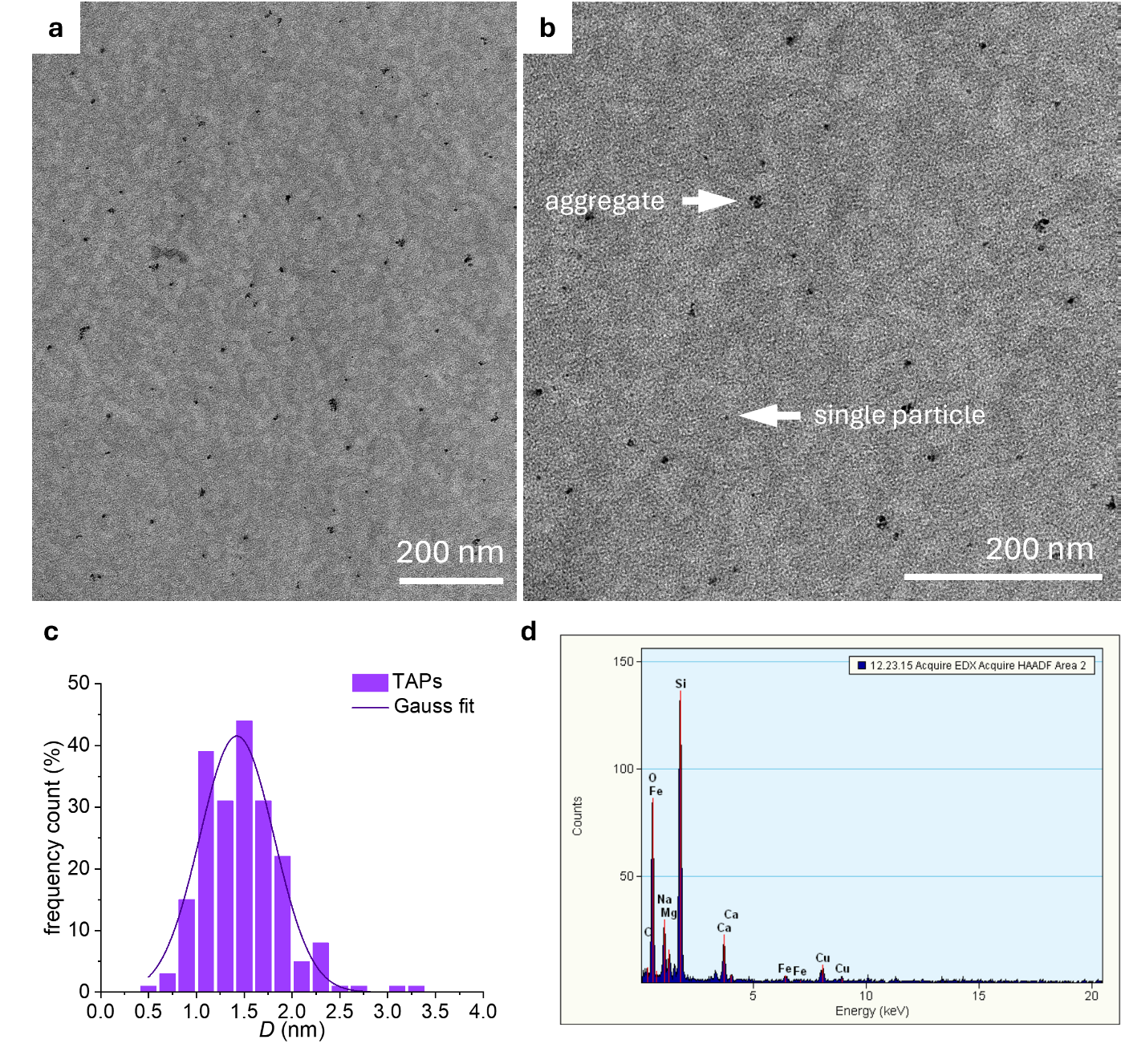


Fig. S 2. (a) and (b)TEM images of TAPs. (c) Size distribution analysis of TAPs by TEM analysis. N_particles_ = 100. (d) EDX-analysis on the power of TAPs.


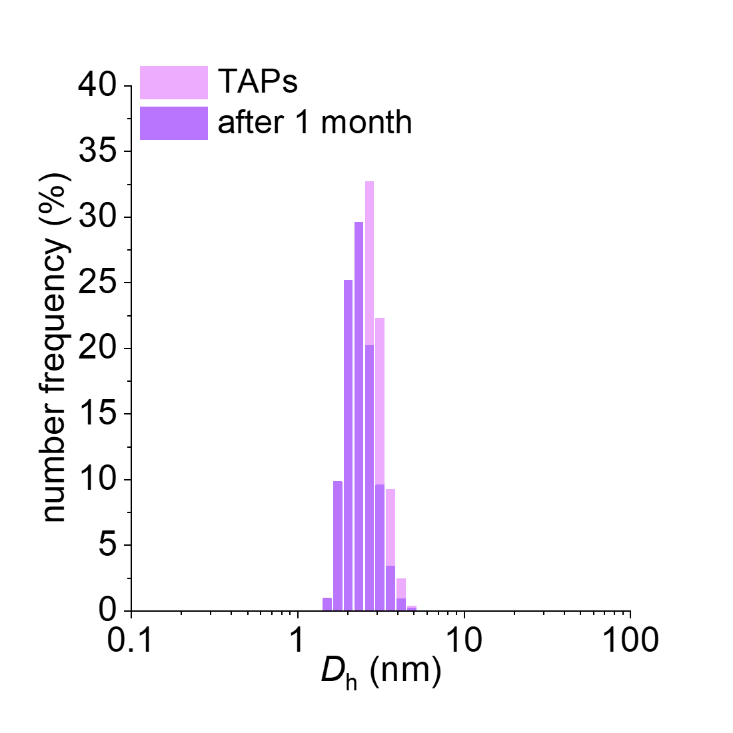


Fig. S 3. DLS-based long term stability study of TAPs.


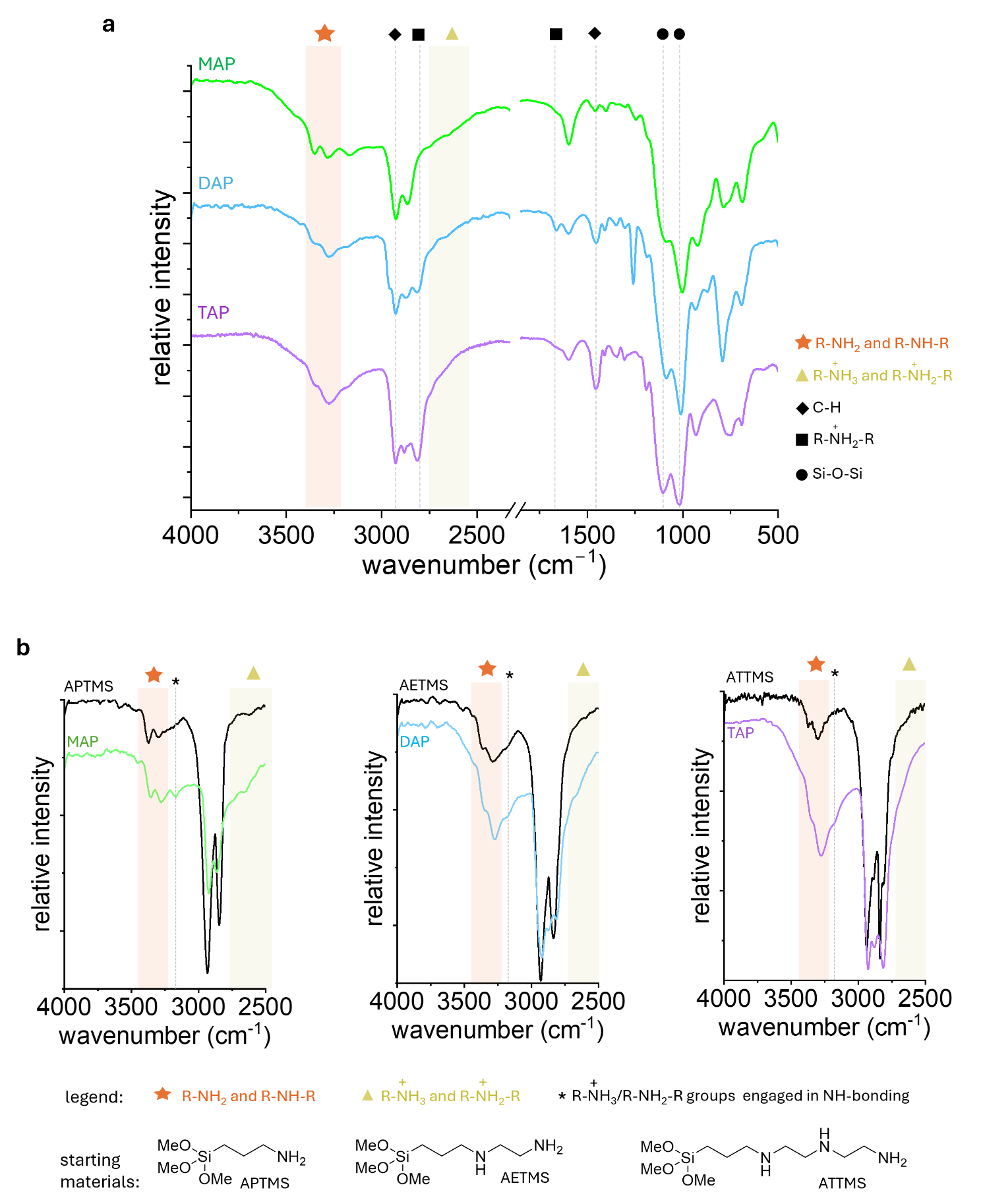


Fig. S 4. ATR-FTIR spectroscopy analysis of MAP, DAP, and TAP powders. (a) ATR-FTIR spectra of the starting materials APTMS, AETMS, and ATTMS overlaid with their corresponding silsesquioxanes, MAP, DAP, and TAP, respectively. The presence of non‑protonated and protonated amino groups is highlighted.


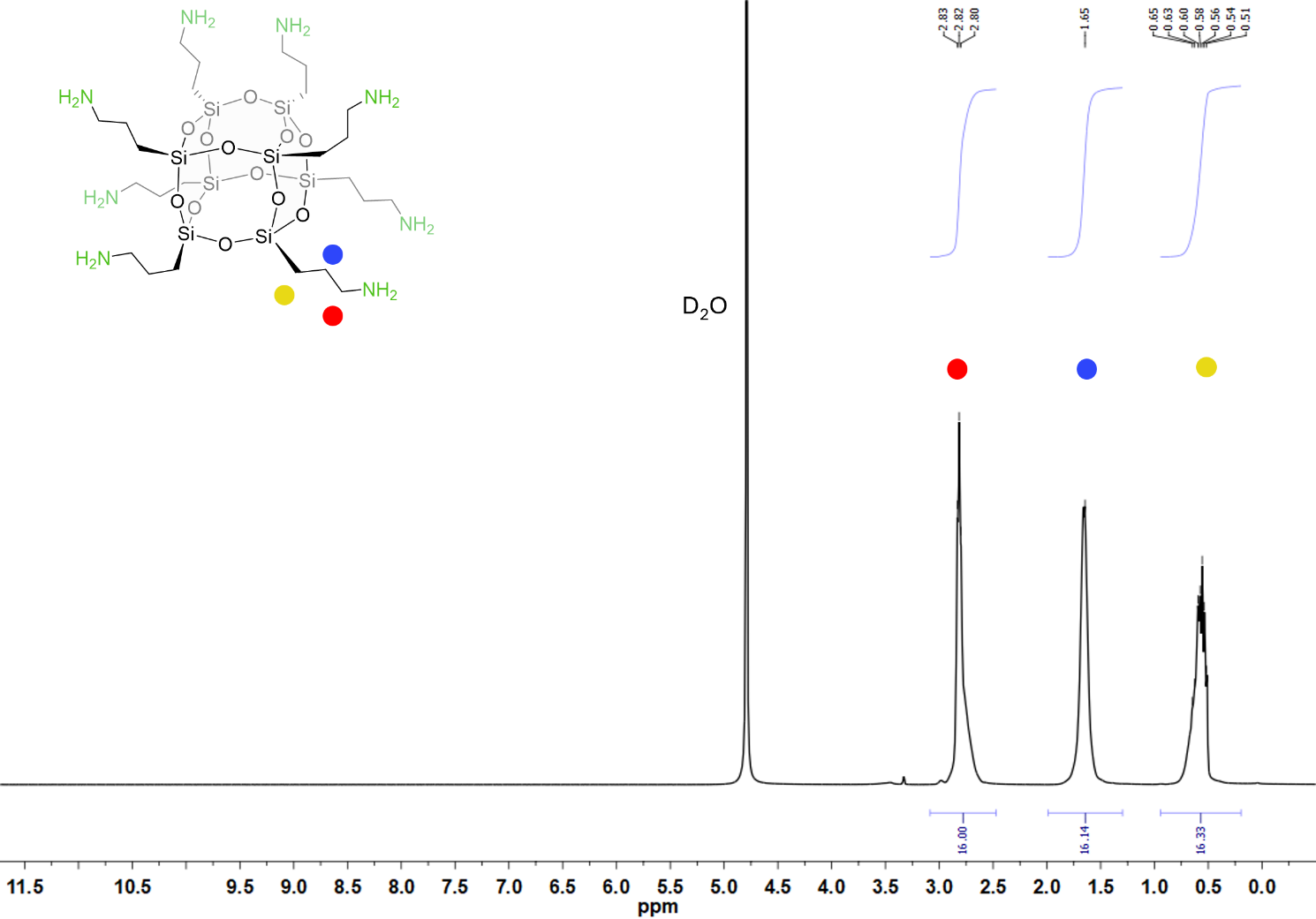


Fig. S 5. ^1^H NMR spectrum (400 MHz in D_2_O) of MAPs.


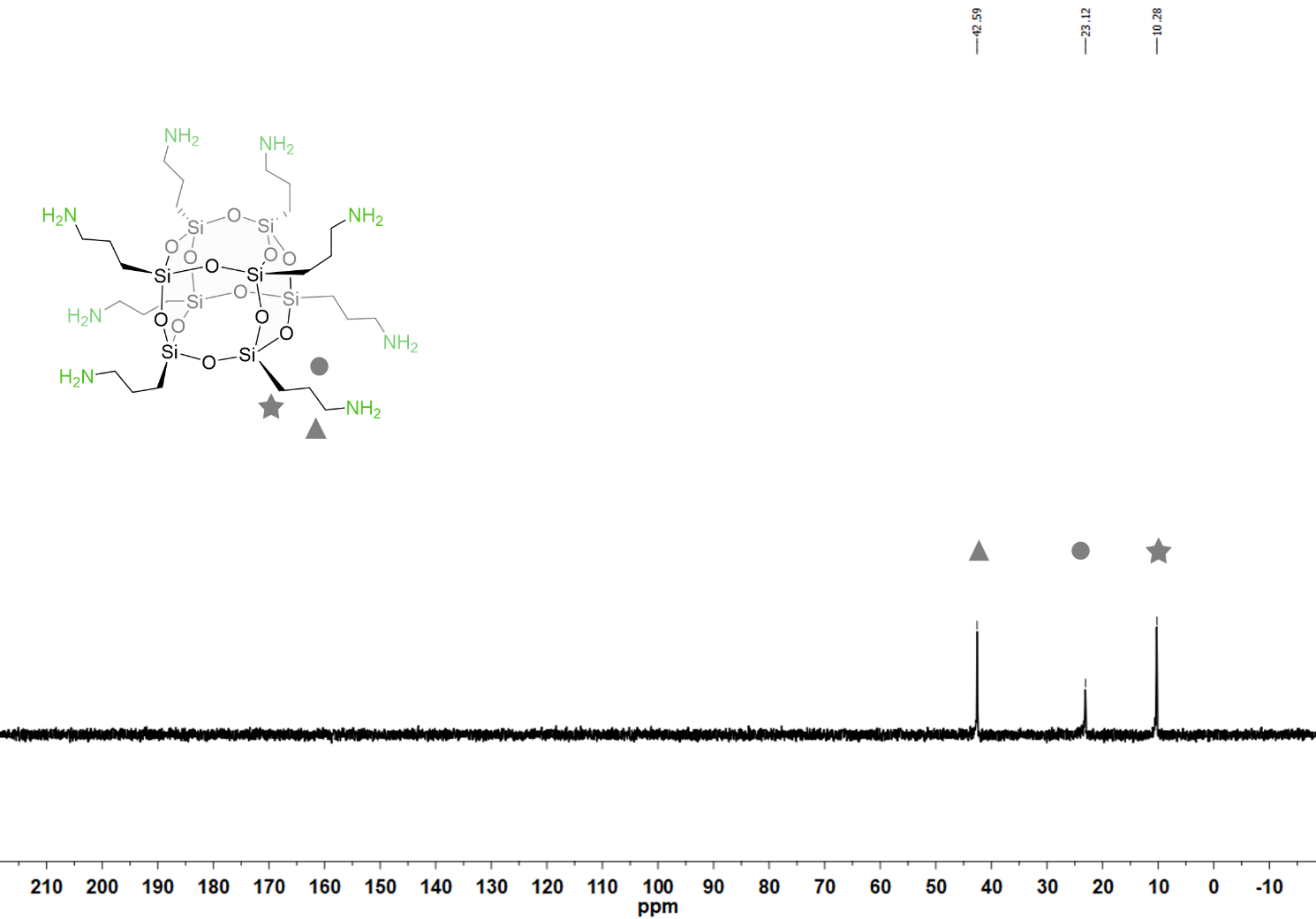


Fig. S 6. ^13^C NMR spectrum (100 MHz in D_2_O) of MAPs in D_2_O.


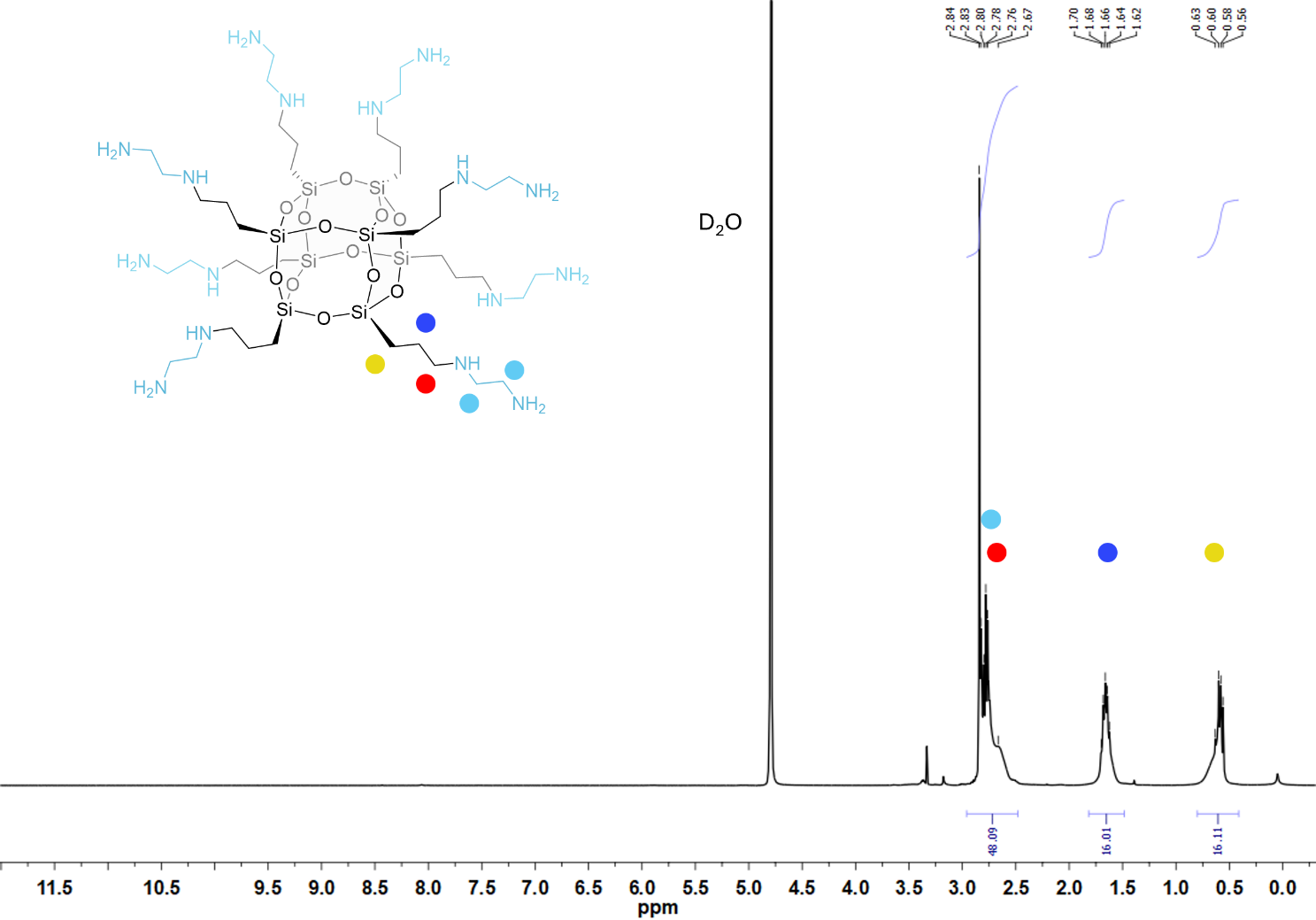


Fig. S 7. ^1^H NMR spectrum (400 MHz in D_2_O) of DAPs.


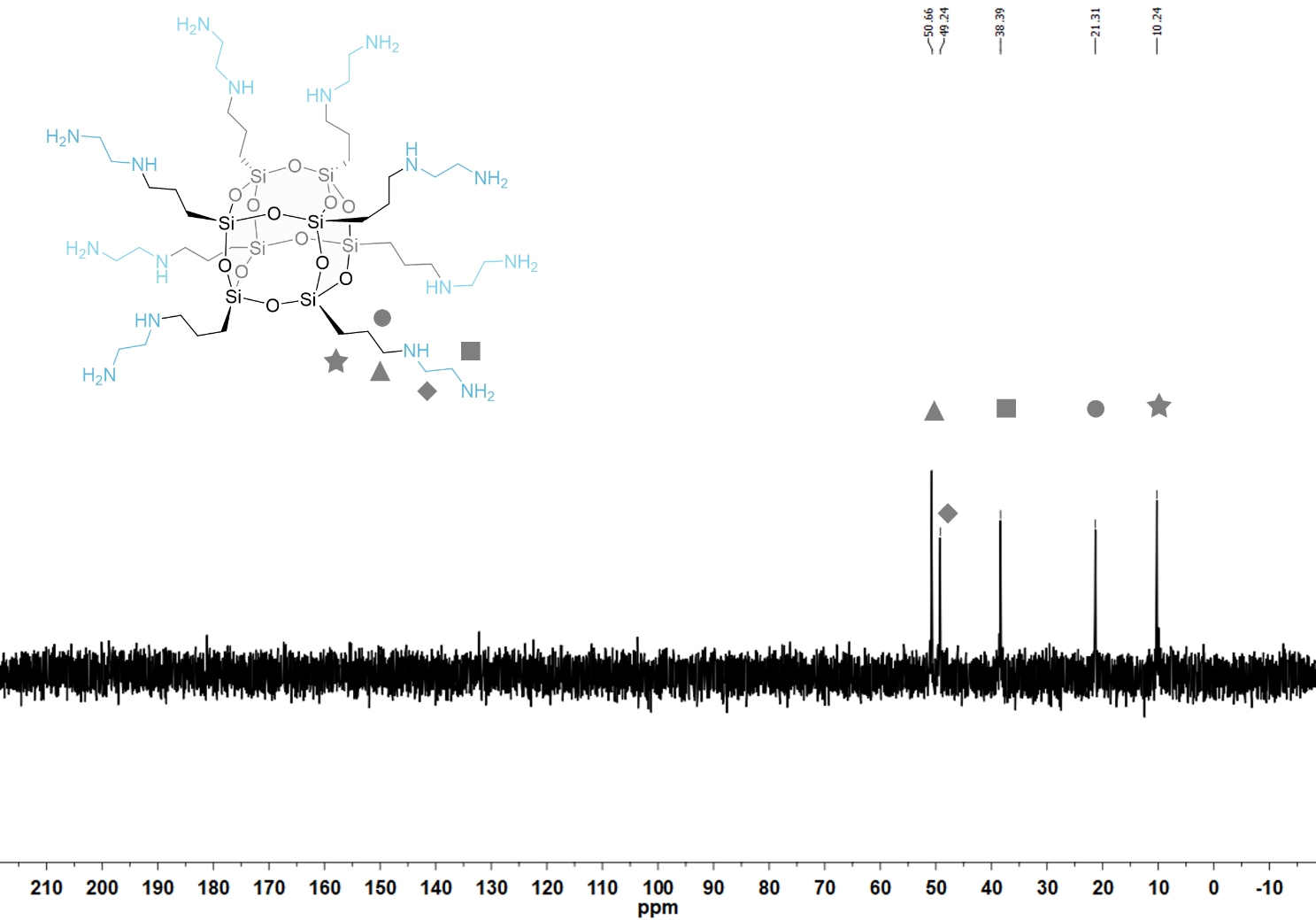


Fig. S 8. ^13^C NMR spectrum (100 MHz in D_2_O) of DAPs in D_2_O.


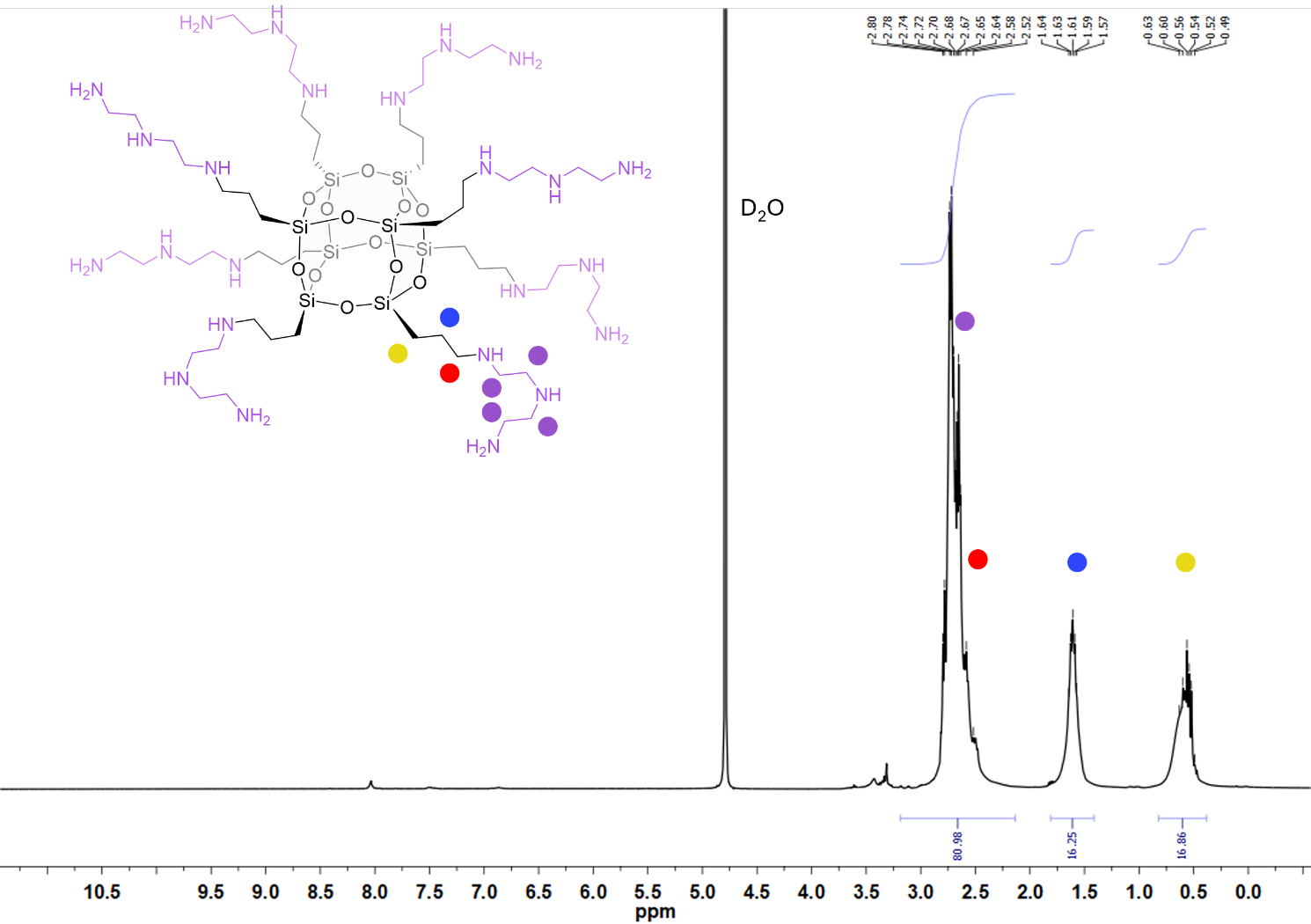


Fig. S 9. ^1^H NMR spectrum (400 MHz in D_2_O) of TAPs.


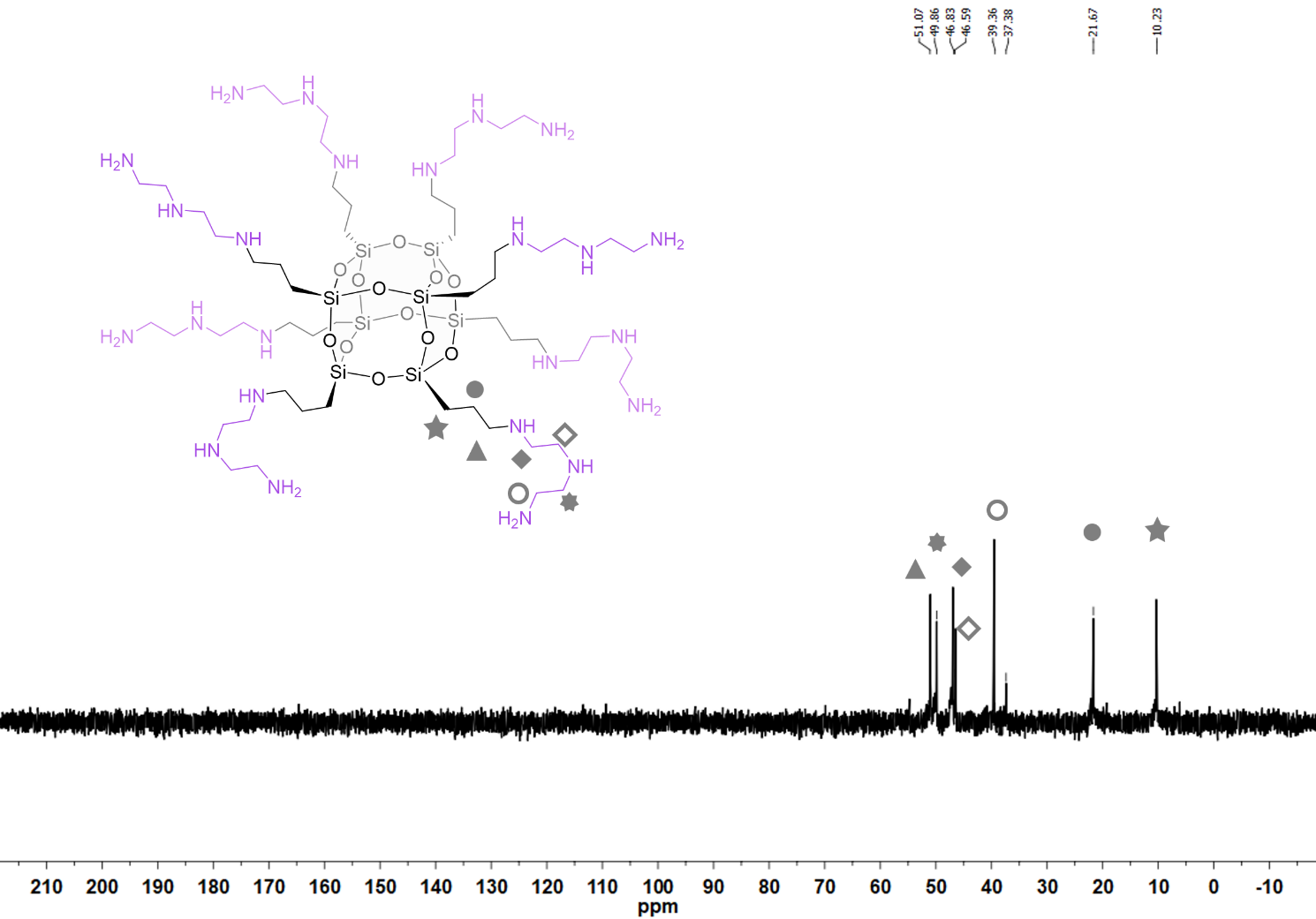


Fig. S 10. ^13^C NMR (100 MHz in D_2_O) spectrum of TAPs.


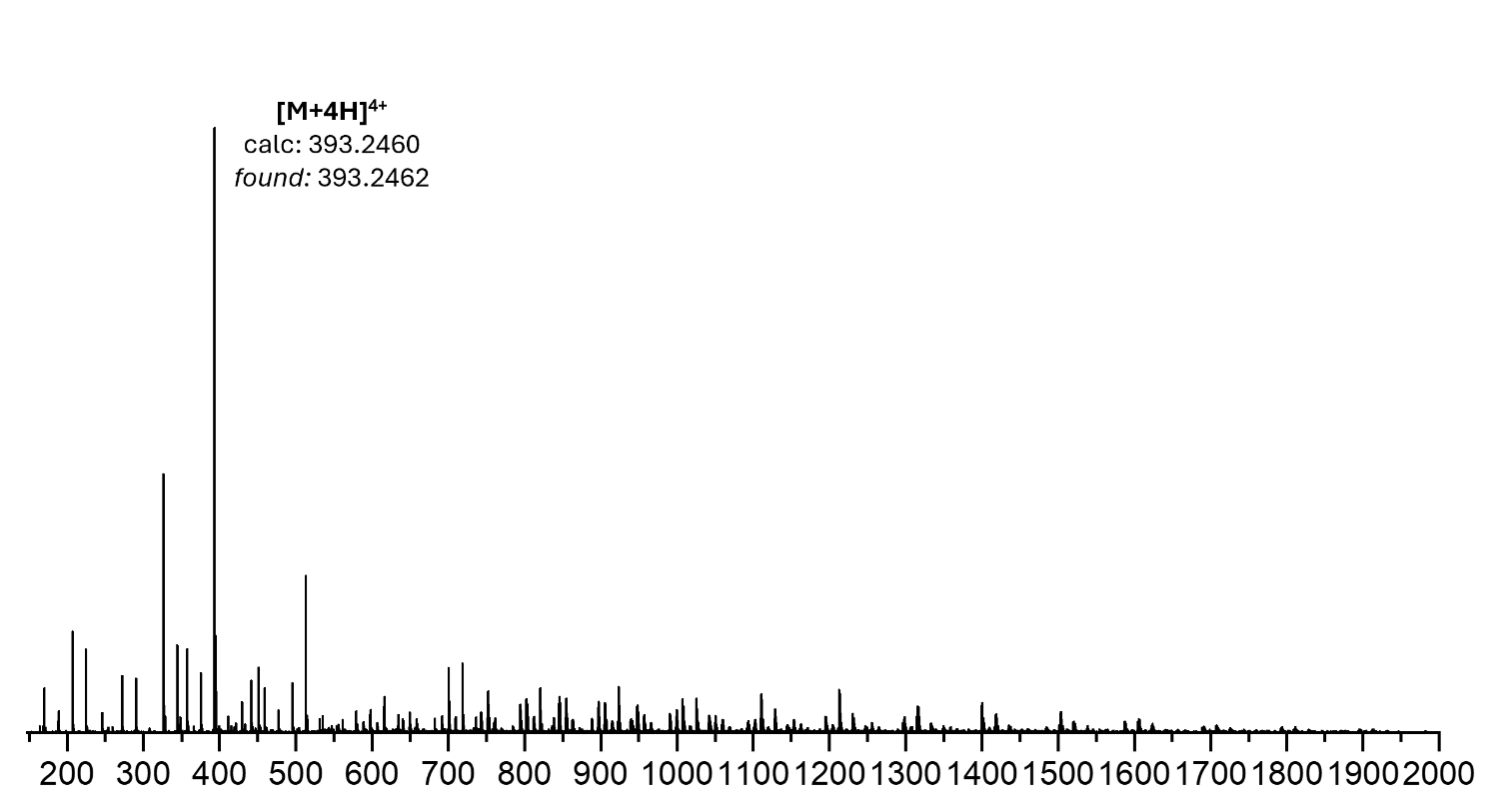


Fig. S 11. MS analysis of TAPs shows a major peak corresponding to the intact parent ion, which is neither open nor fragmented, indicating that the cage-like structure remains stable in solution and even under ionizing ESI conditions.


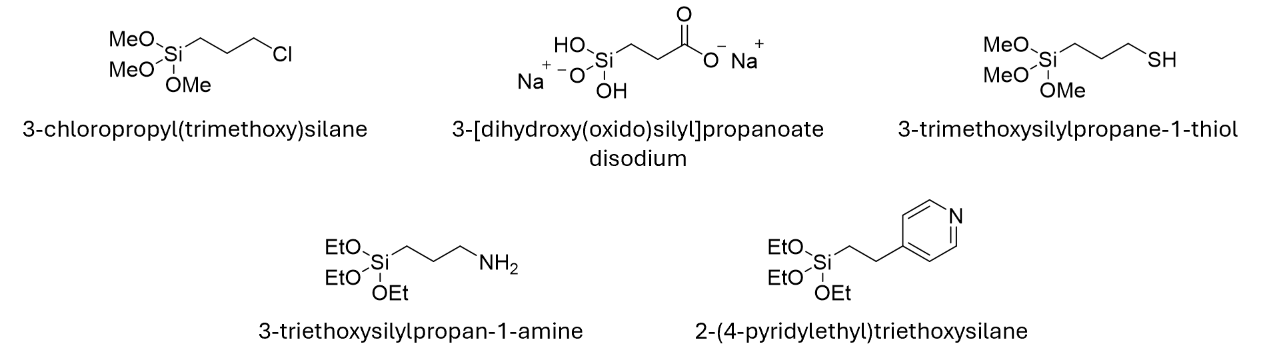


Fig. S 12. Organotrialkxysilanes that yielded no octahedral silsesquioxanes. The use of 3-chloropropyl(trimethoxy)silane, 3-[dihydroxy(oxido)silyl]propanoate disodium, 3-trimethoxysilylpropane-1-thiol, and 2-(4-pyridylethyl)triethoxysilane resulted only in the formation of large, aggregated amorphous precipitates, indicating the necessity of sufficiently basic amino groups under the applied synthesis conditions. In contrast, when 3-triethoxysilylpropan-1-amine was employed, the formation of apparent colloidal dispersion occurred. However, the resulting particles were within a DLS size range of way above 300 nm and as such not utilized for further studies.


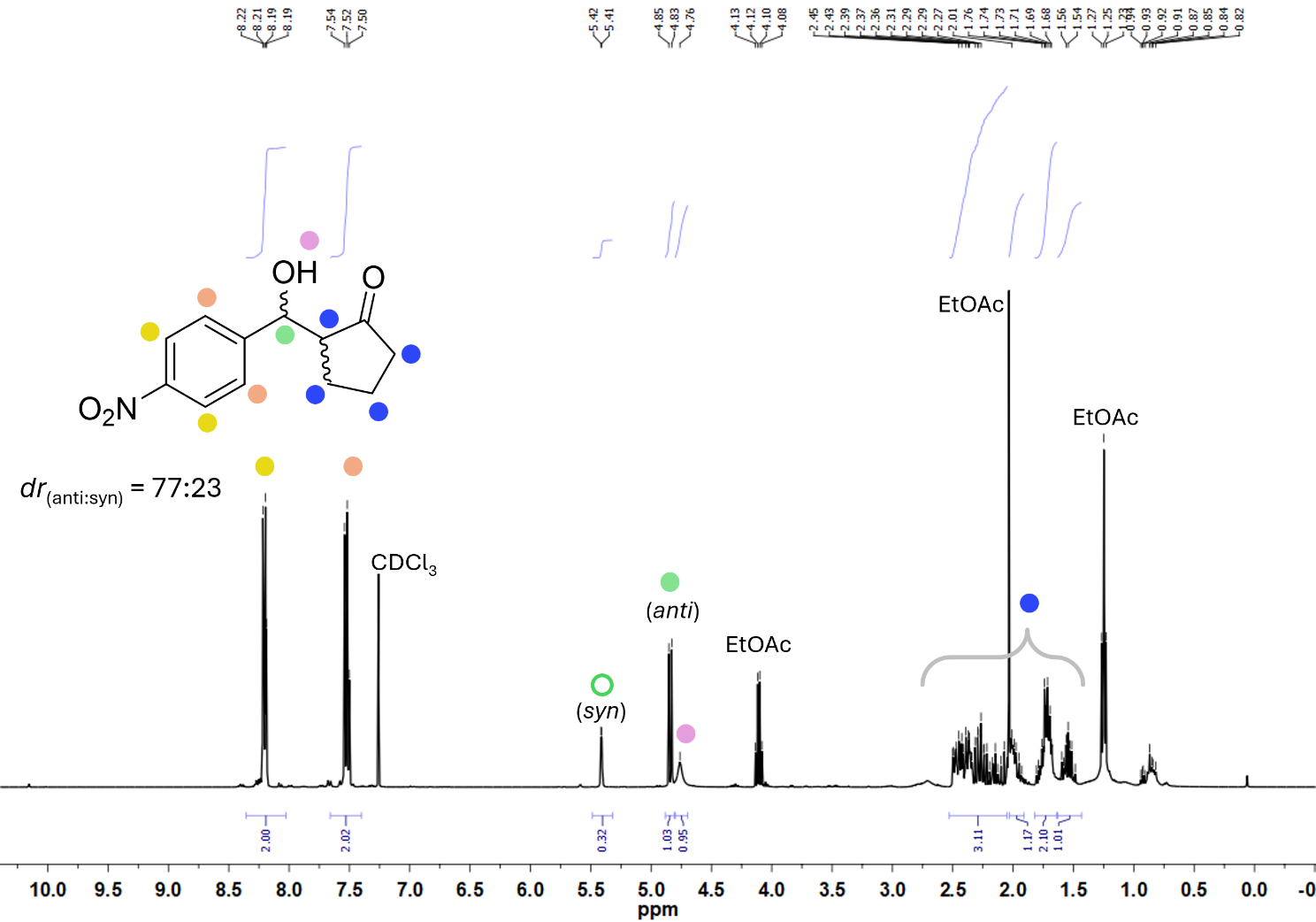


Fig. S 13. ^1^H NMR spectrum (400 MHz in CDCl_3_) of PR obtained from TAP catalyzed reaction of NBA and CP.


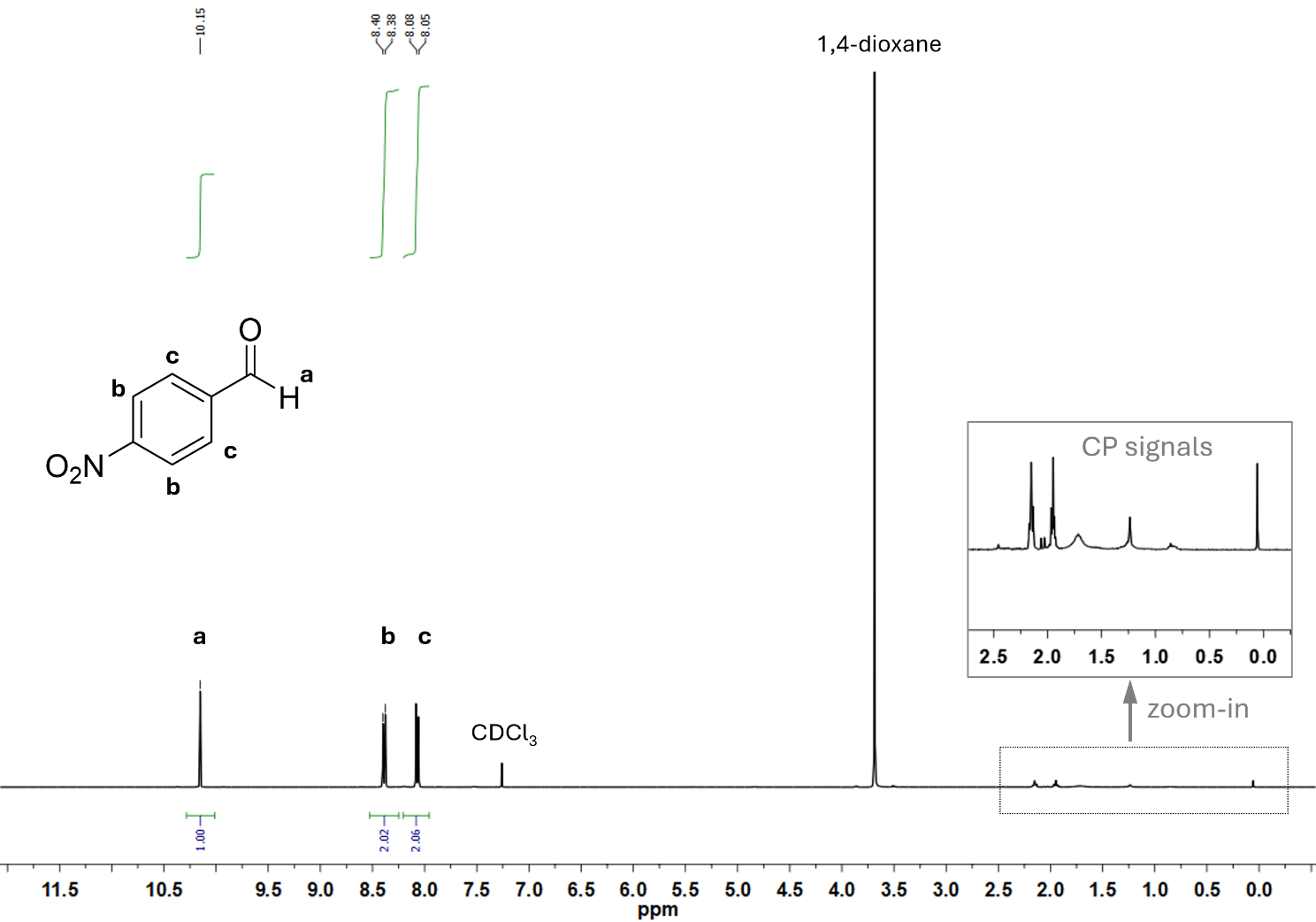


Fig. S 14. ¹H NMR spectrum (400 MHz in CDCl_3_) of the control reaction mixture extract containing NBA and CP in the absence of TAPs.


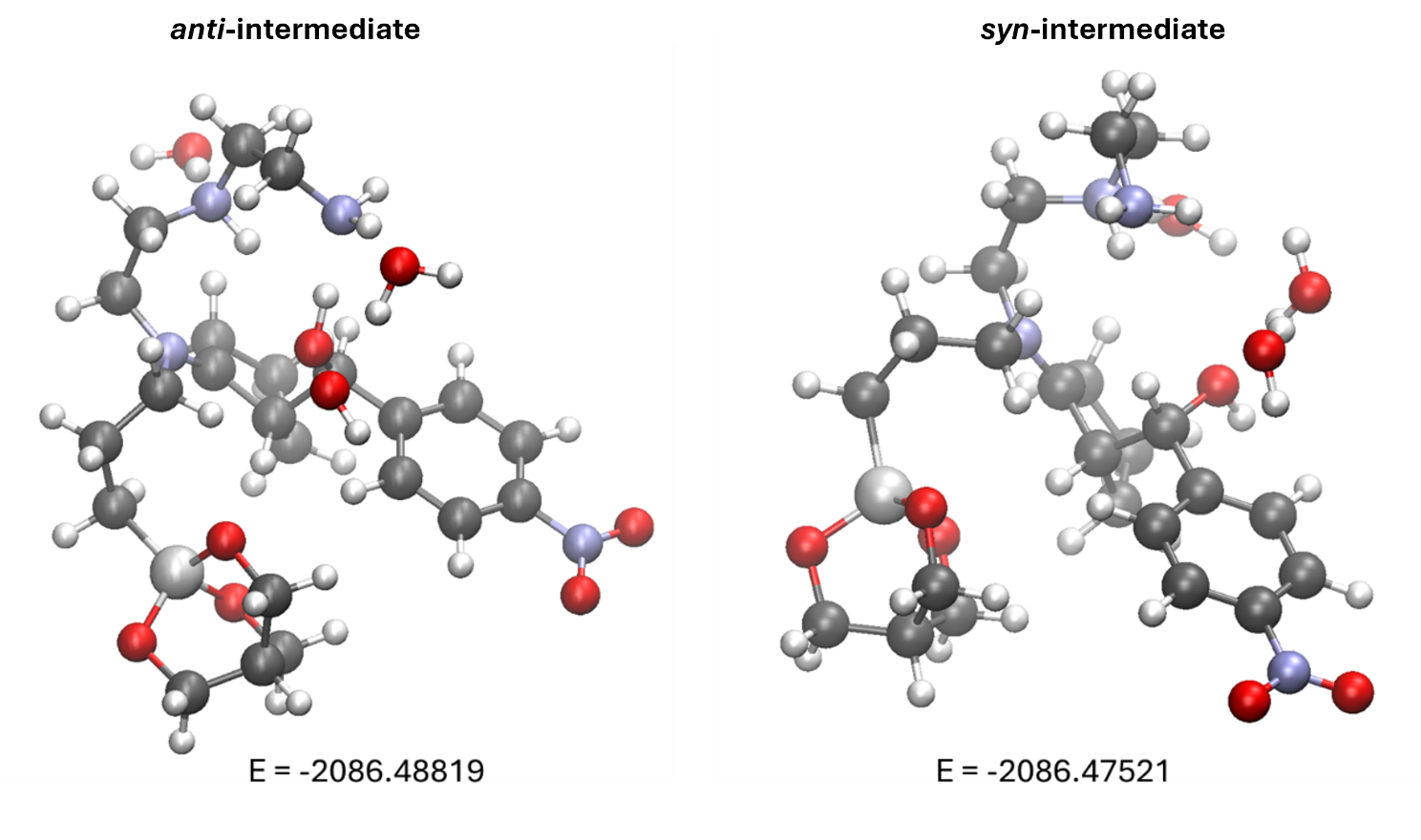


**Fig. S 15**. Optimized structures (PBE0-D4/def2-TZVPP/CPCM) of the anti and syn intermediaries of the aldol reaction, including explicit microsolvation (3H_2_O) showing the absolute computed energies (Hartree). The energetic difference accounts for a favorable stabilization of roughly 8 kcal·mol^−1^, for the anti-intermediate conformation.


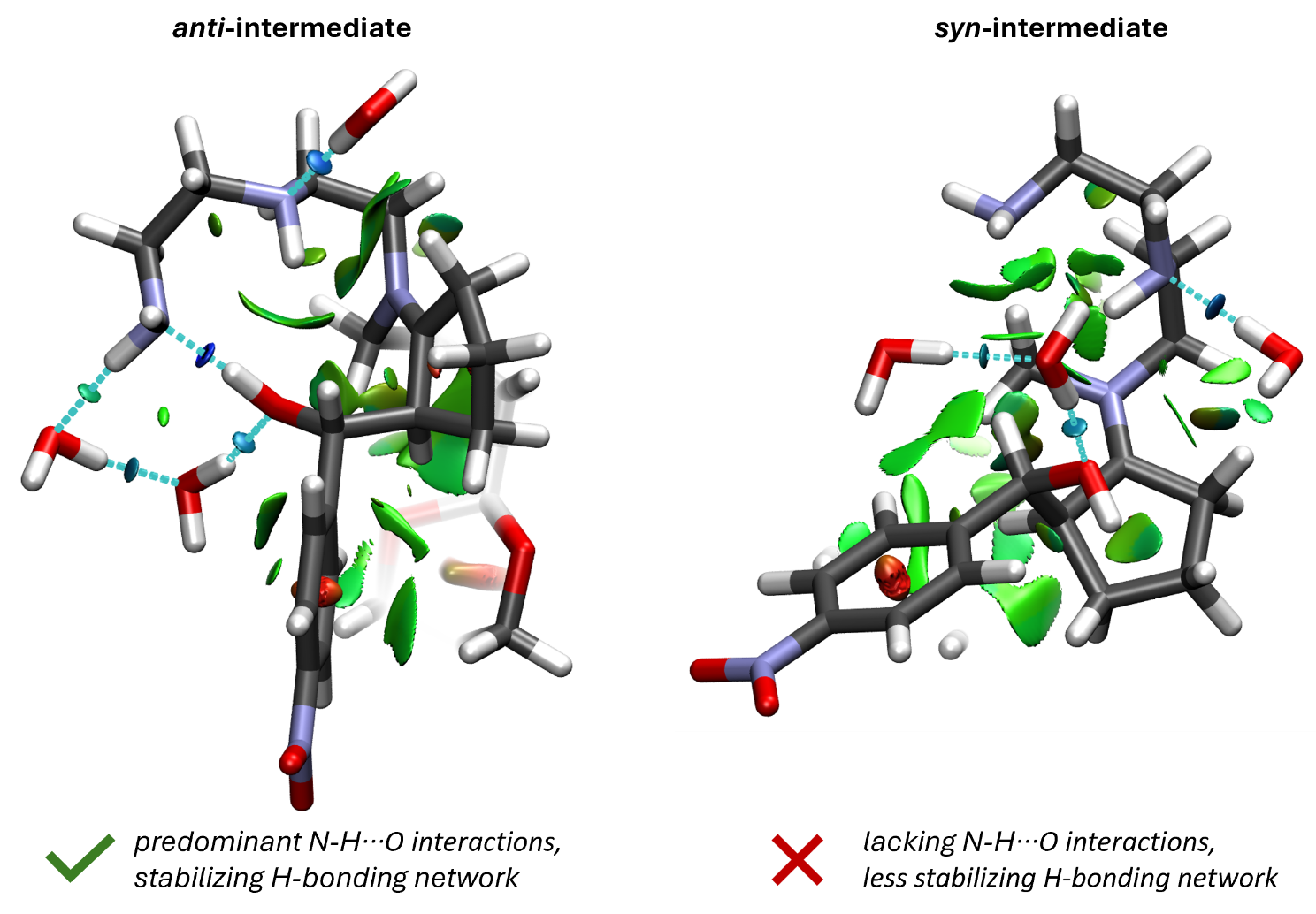


**Fig. S 16**. NCI analysis of the truncated DFT model (PBE-D4/def2-TZVPP)^5^ optimized intermediate structures, showing the stabilization of the syn conformer NCI plots visualize non-covalent interactions via isosurfaces of the second eigenvalue of the electron density Hessian (λ₂ρ), where blue/green regions (λ₂ρ < 0) indicate, respectively, strong or weak attractive interactions and red (λ₂ρ > 0) show steric repulsion. The colour code is as follows C = gray, N = blue, O = red, and Si = silver, the visualization was generated using VMD.^13^


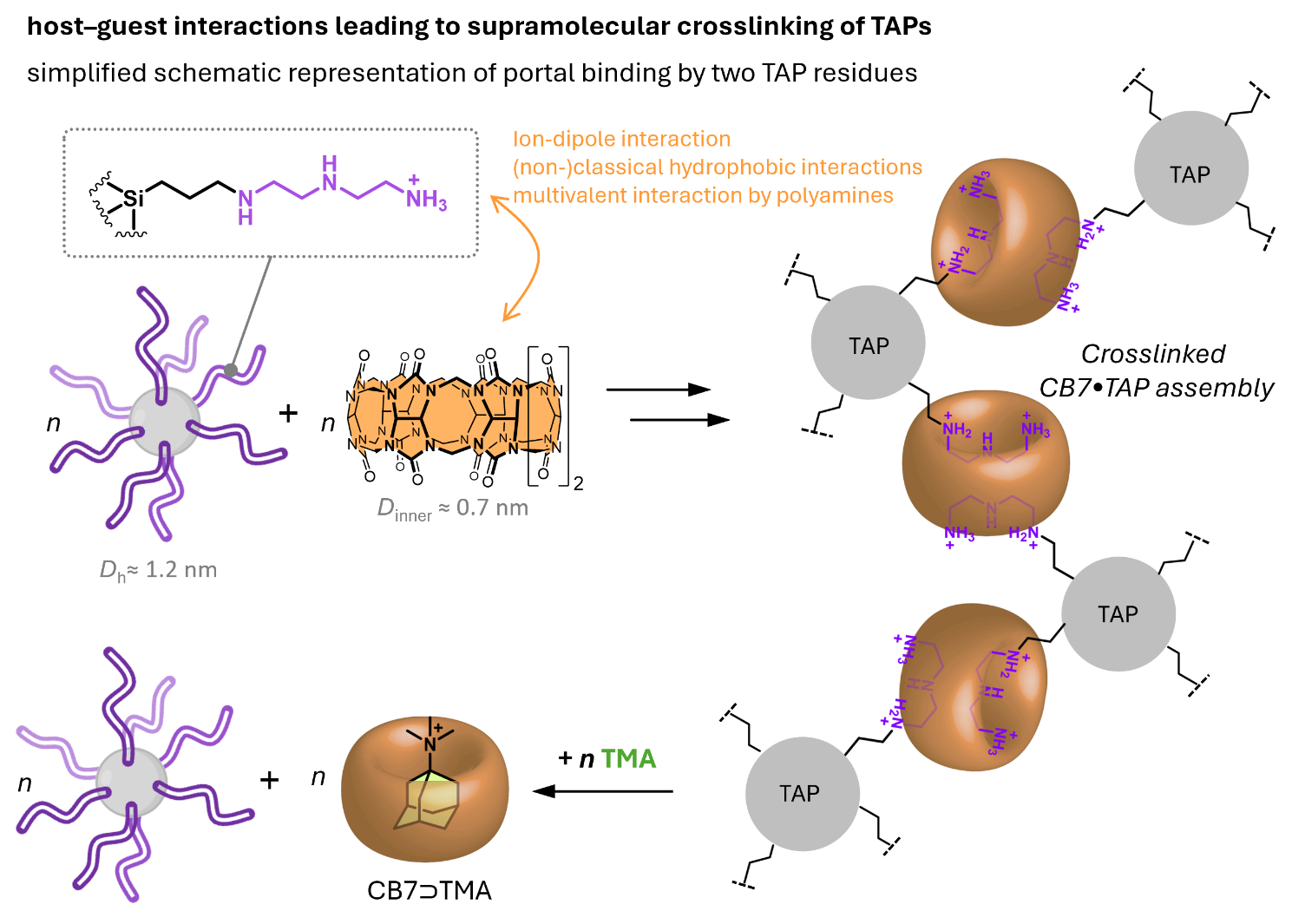


**Fig. S 17**. Schematic representation of TAP–CB7 association and competitive displacement by TMA. Protonated surface polyamines on TAPs engage the carbonyl‑lined portals of CB7 through strong ion–dipole interactions, complemented by (non‑)classical hydrophobic inclusion of adjacent alkyl segments and multivalent binding of several amine sites on the same particle. Because of steric constraints, TAPs form an association complex in which CB7 bridges surface amine residues rather than fully encapsulating a single guest, a behavior well documented for ammonium compounds with CB7 and consistent with our experimental observations and the broader host–guest literature. In the final step, addition of TMA leads to formation of the much higher‑affinity CB7⊃TMA complex, which competitively displaces the medium‑affinity polyamine chains on TAPs and thereby drives disassembly of the TAP·CB7 aggregates.


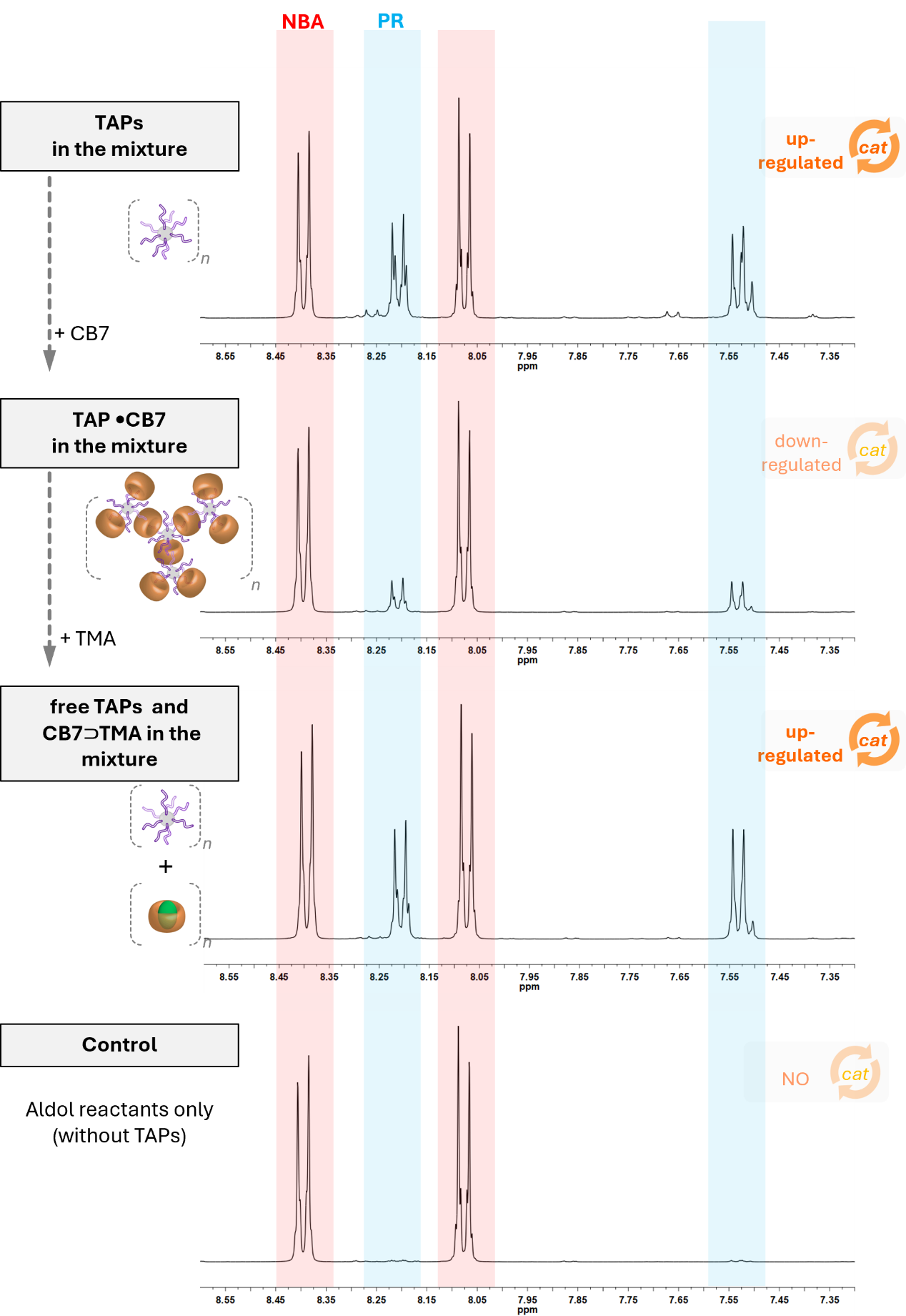


Fig. S 18. ¹H NMR spectra of the aldol reactions were recorded in the region where the indicative signals of NBA and PR (aromatic resonances) appeared and were used for integration studies. The addition of CB7 inhibited the formation of PR-indicative signals (highlighted in blue), whereas in the absence of CB7 or in the presence of TMA, their intensities were significantly increased.


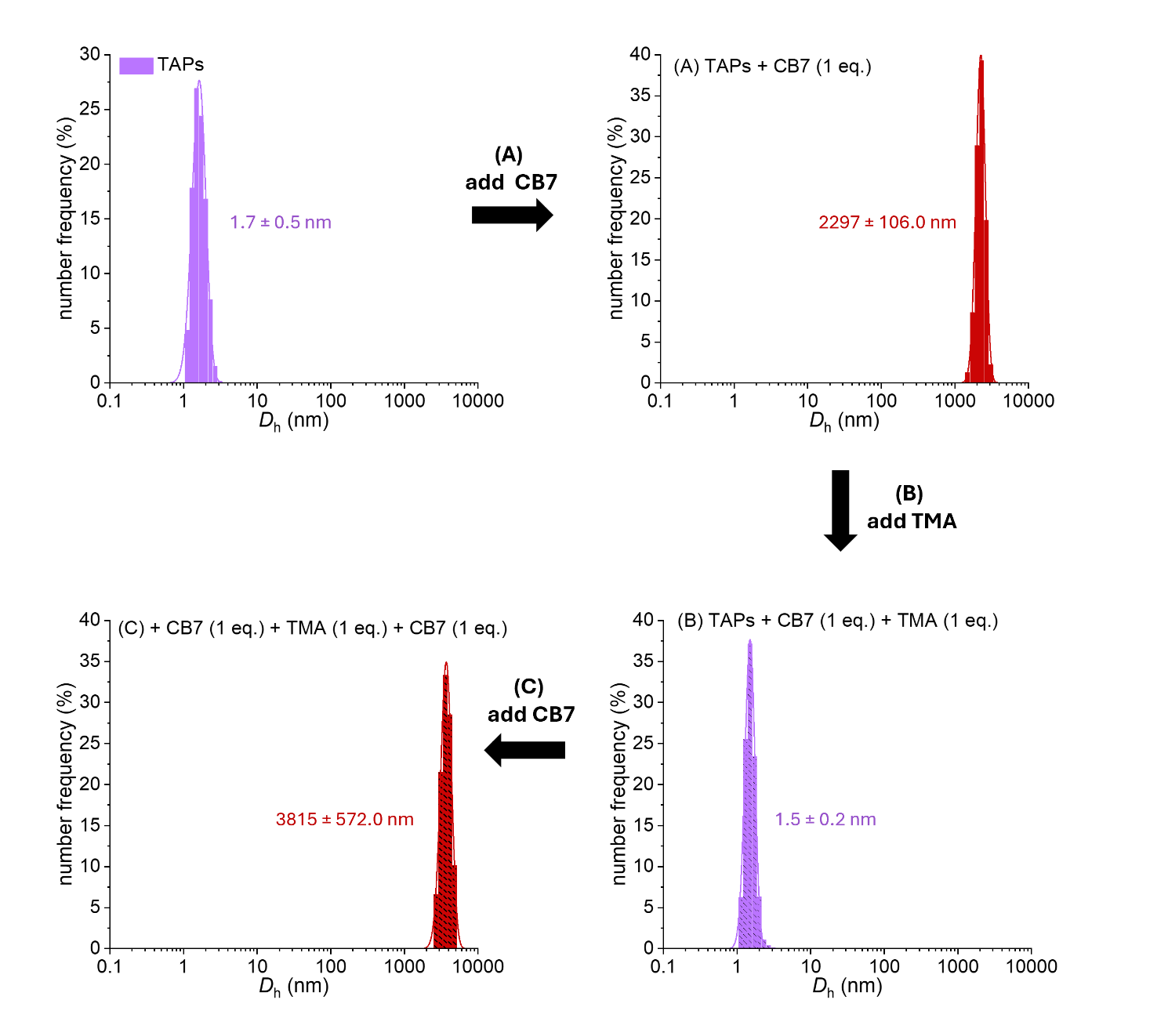


Fig. S 19. Aggregation and disaggregation of TAPs were monitored by DLS following sequential additions of CB7 (1 eq.), TMA (1 eq.), and a second addition of CB7 (1 eq.). As mentioned, the addition of CB7 enables TAPs crosslinking leading to the formation of micrometer sized TAP•CB7 aggregates (step A). By a following addition of the high affinity guest TMA for CB7, the TAP•CB7 disassemble leading to free TAPs and the formation of a CB7


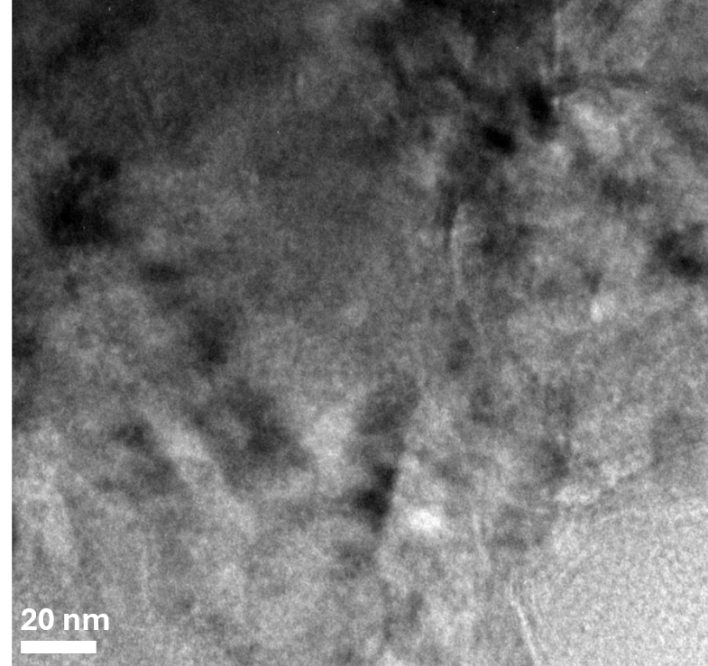


Fig. S 20. TEM image of TAPs that have been mixed with CB7 (1 eq.) to form TAP•CB7.


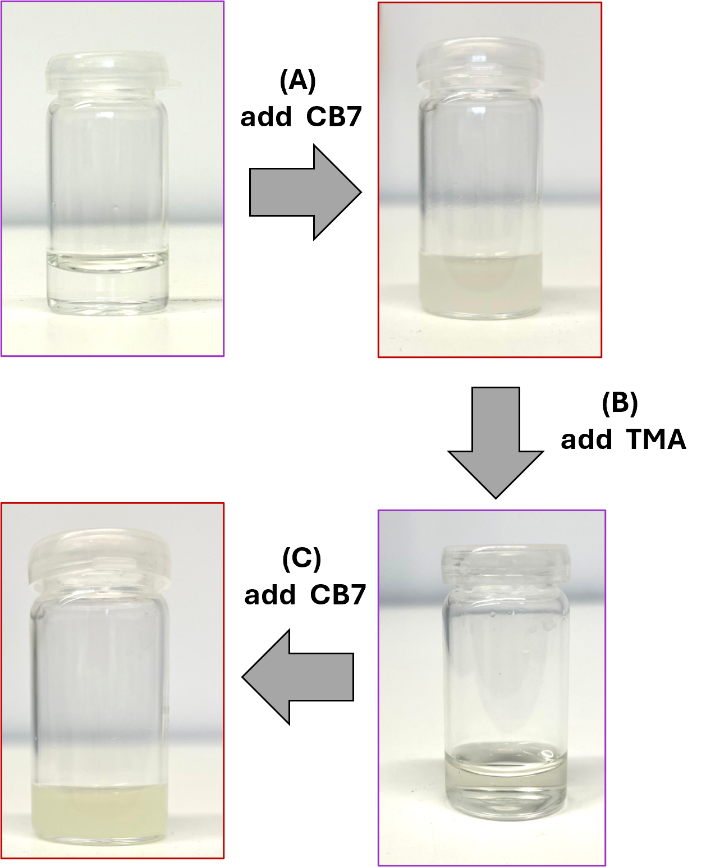


Fig. S 21. Images of TAPs colloidal dispersions upon sequential addition of CB7, followed by TMA, and finally CB7 again. The increase in turbidity after the first CB7 addition is attributed to the formation of micrometer-sized TAP•CB7 aggregates. Upon addition of TMA, these aggregates disassemble, releasing free TAPs due to the formation of the more stable and water-soluble CB7⊃TMA host–guest complex. A subsequent addition of CB7 leads again to crosslinking of TAPs and aggregate formation.


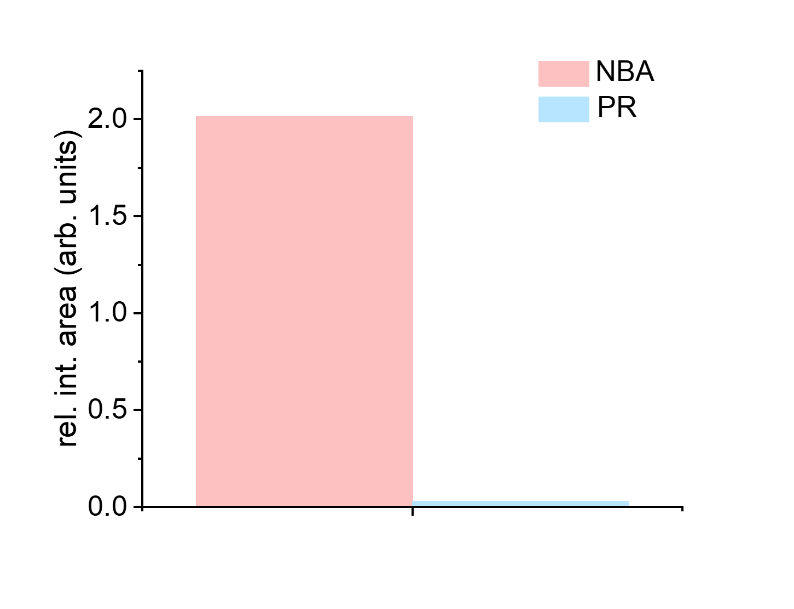


Fig. S 22. Relative NMR integration areas of NBA and PR when catalysis was performed in the absence of TAPs.


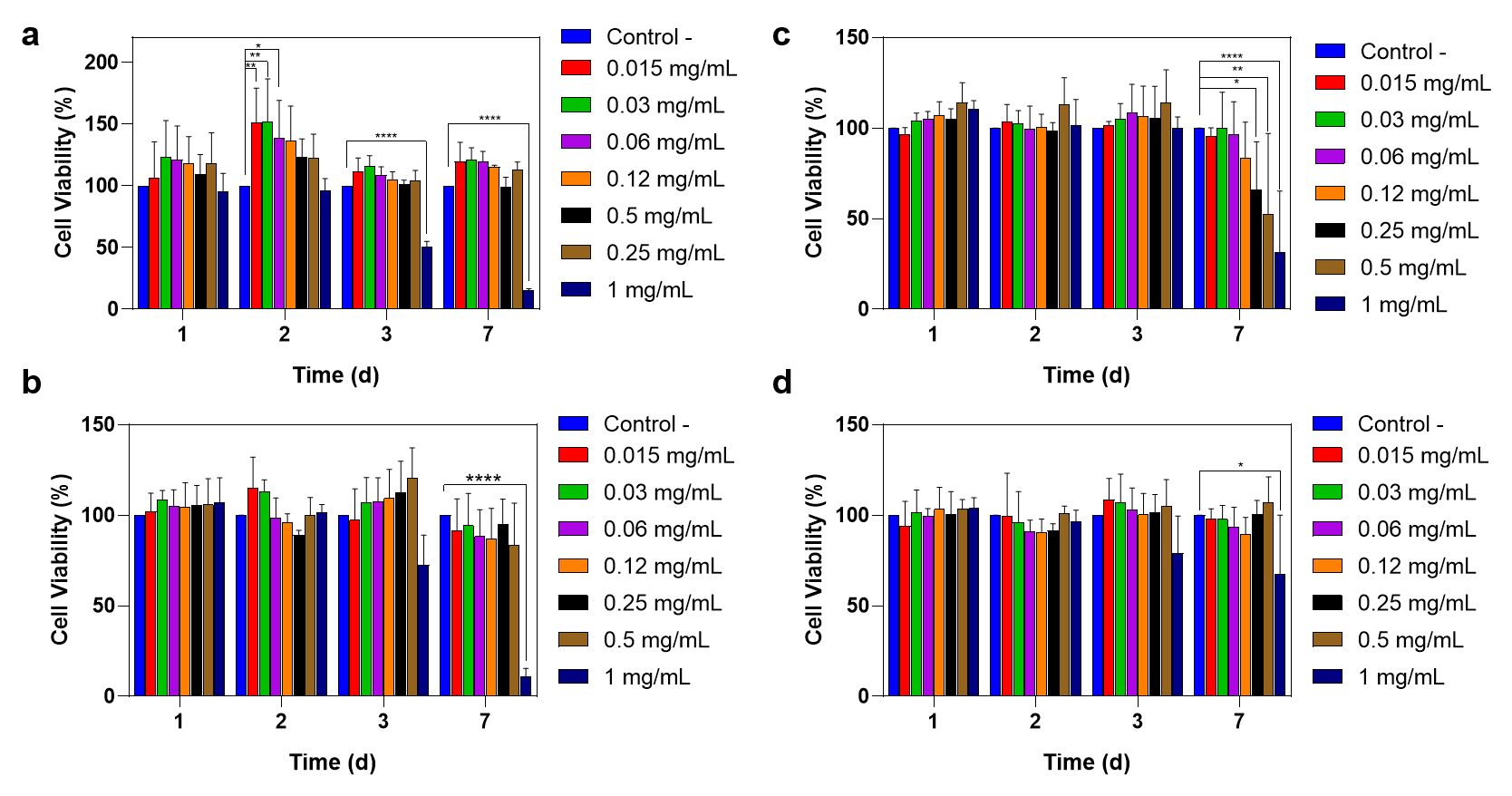


**Fig. S 23**. Biocompatibility study of TAPs on (a) human fibroblasts, (b) human mesenchymal stem cells, (c) murine fibroblasts and (b) murine mesenchymal stem cells during 1, 2, 3 and 7 days.


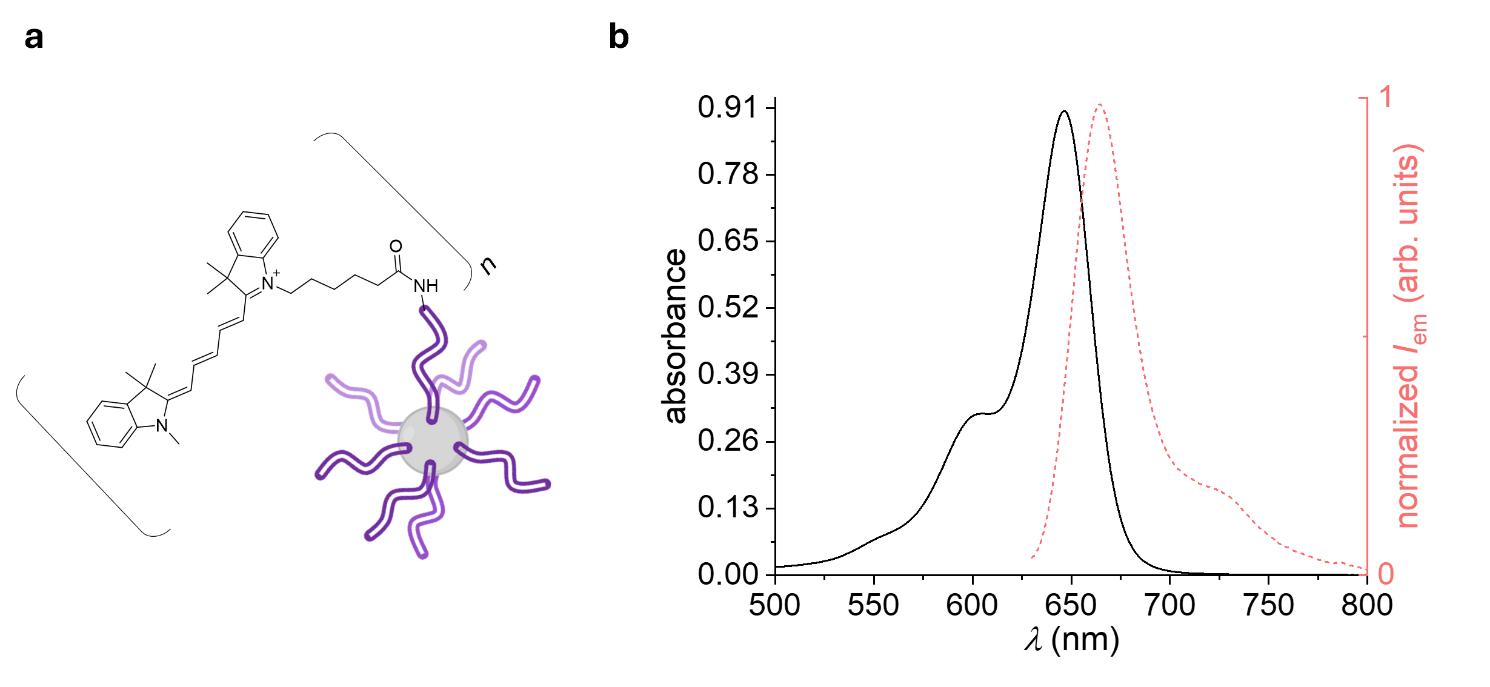


Fig. S 24. Schematic representation of Cy5-functionalized TAPs. (b) Absorbance spectrum (*c*_TAPs_ = 10 mg·mL^−1^) and normalized emission spectrum (*λ*_em_ = 620 nm) of TAPs (0.1 mg·mL^−1^) recorded in water (pH = 7.0).


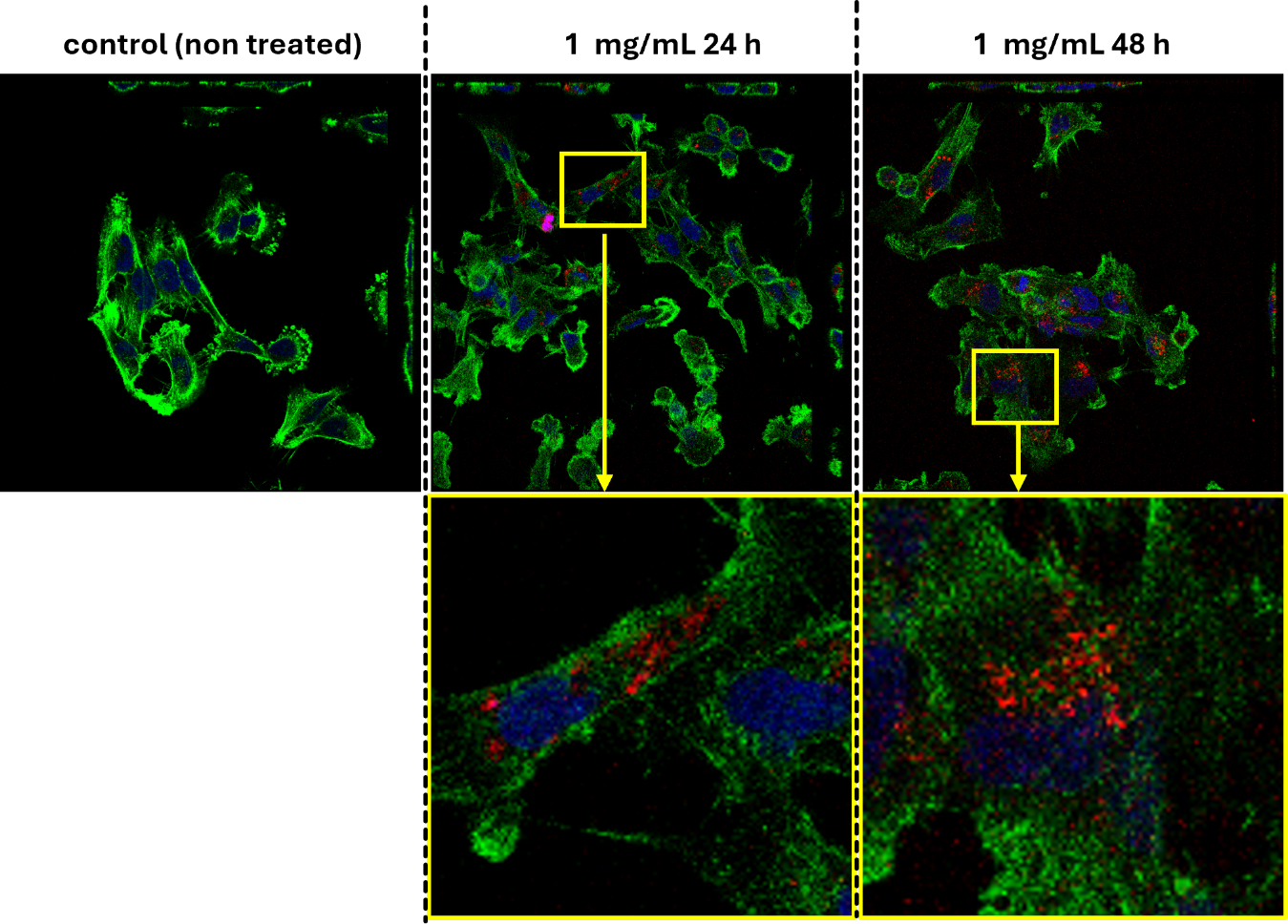


**Fig. S 25**. Confocal microscopy analysis on the cell uptake study of cyanine5-labelled TAPs in U-251MG malignant glioblastoma cells.


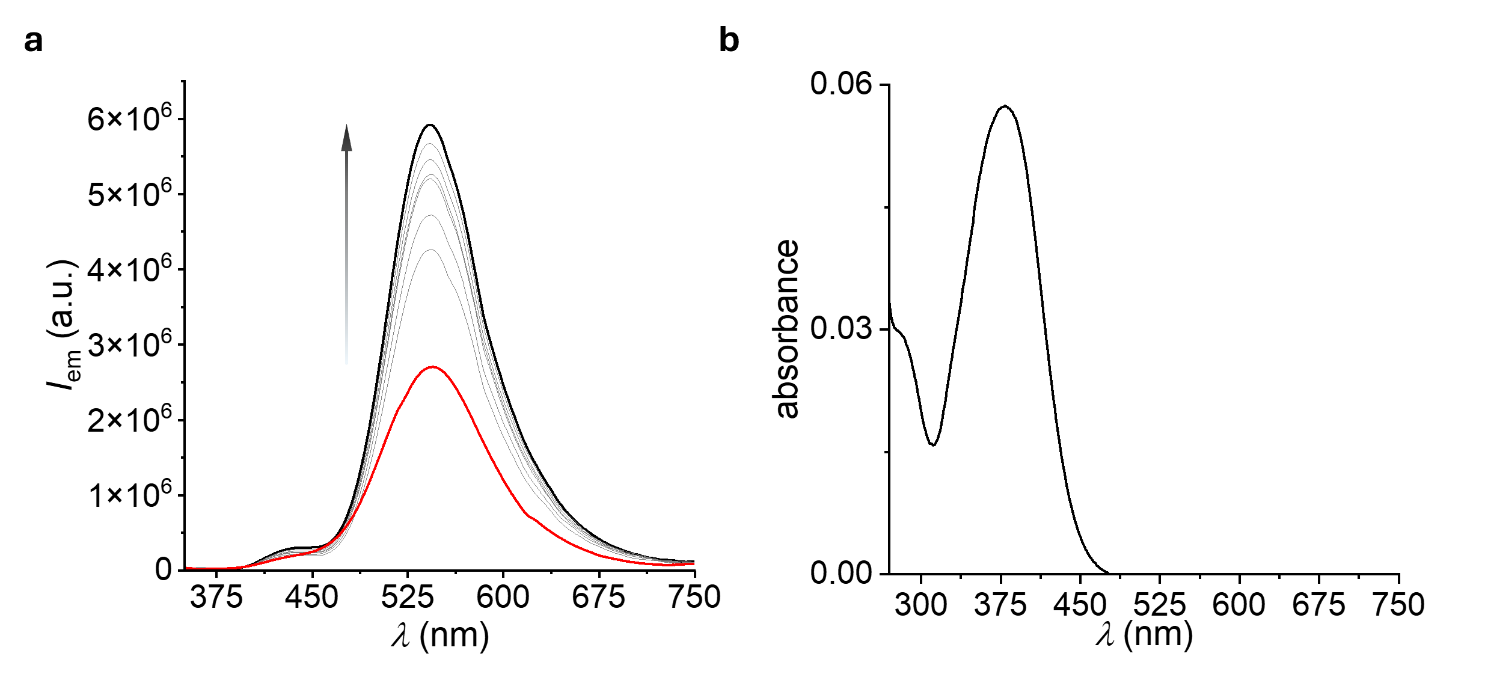
Fig. S 26. (a) Luminescence spectra recorded for the TAPs-catalyzed retro-aldol reaction of DANO (3.13 µmol, in 10 mM PBS at pH 7.0) with TAPs (5 eq.). The time points correspond to: luminescence spectrum recorded immediately after TAP addition (—); after 3.5 h of mixing (—). (b) UV–Vis absorption spectrum of the DANO solution (3.13 µmol, in10 mM PBS, pH 7.0, *V*_tot_ = 2.5 mL) recorded before the addition of TAPs.


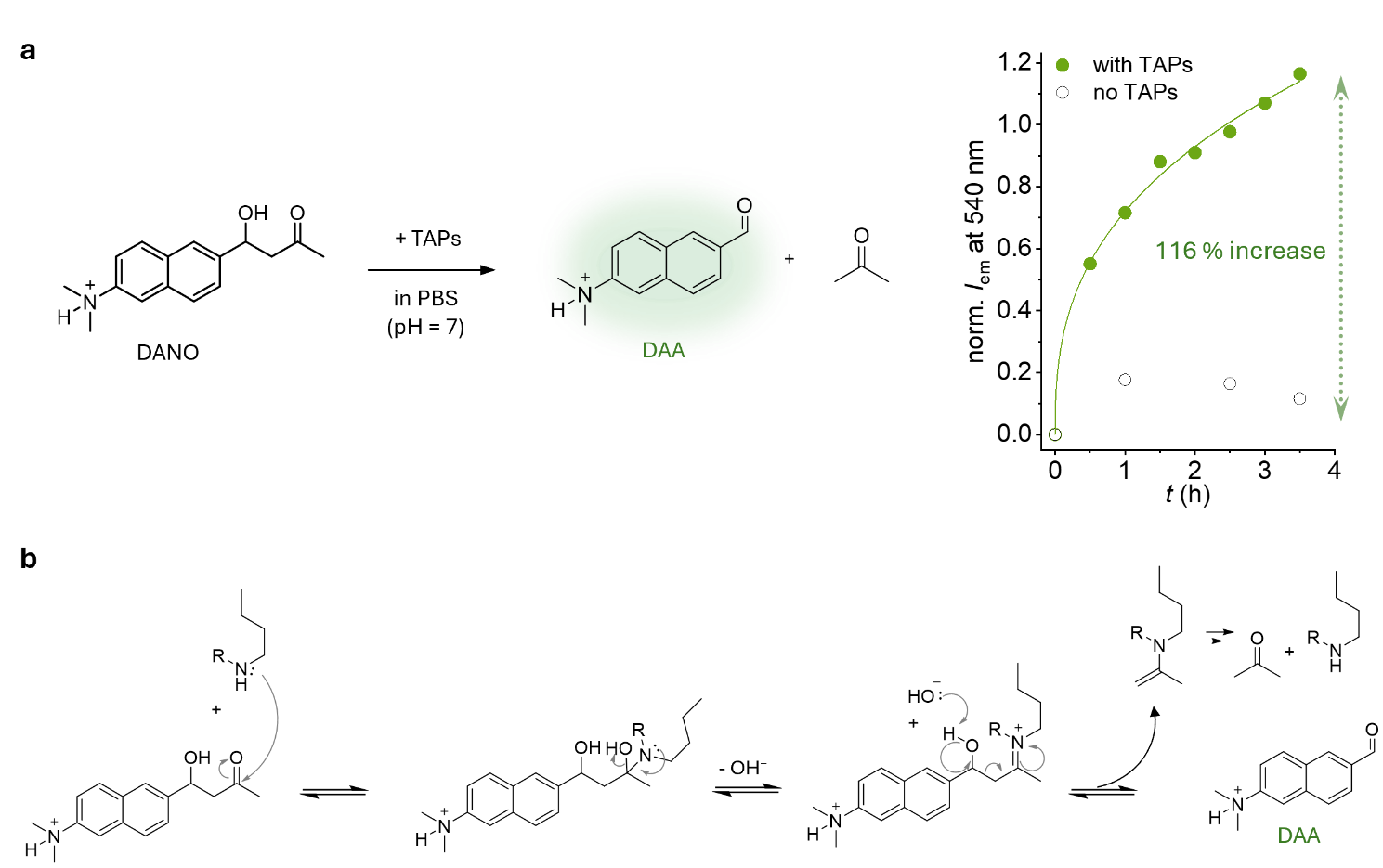


**Fig. S 27**. (a) Retro-aldol reaction of DANO promoted by TAPs. (b) Luminescence intensities (*λ*_ex_ = 305 nm) recorded upon mixing DANO with TAPs and in the absence of TAPs in PBS (10 mM, pH 7.0). (b) Generally accepted reaction mechanism for the amine-promoted retro-aldol reaction.


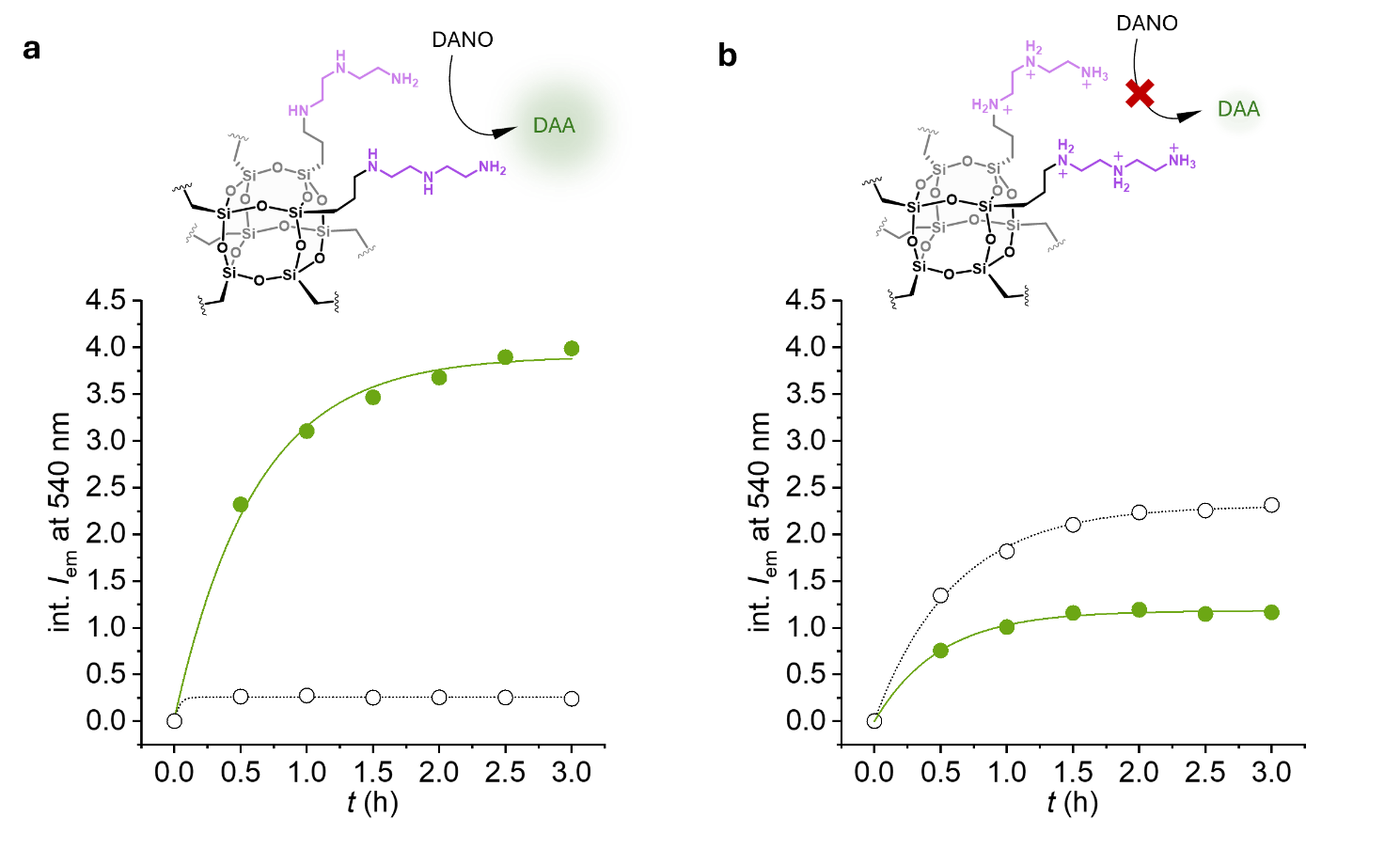


**Fig. S 28**. pH-dependent formation of DAA in the presence and absence of TAP at (a) pH 8.0 and (b) pH 5.5 in 10 mM phosphate buffer. The TAP particles bear primary and secondary polyamines that catalyze the retro-aldol cleavage of DANO via a classical amine–enamine mechanism. Catalytic activity depends strongly on pH. As seen from the luminescence data, at pH 8 the luminescence intensity reaches ~4, after 3 h, whereas at pH 7.4 the increase is ~1.2 and at pH 5.5 no catalytic enhancement is observed compared to DANO alone. This trend is consistent with the expected protonation behavior of aliphatic amines. At acidic pH, the polyamines are predominantly protonated and thus poorly nucleophilic, which suppresses formation of the imine/enamine intermediates required for retro-aldol cleavage. At basic pH, a larger fraction of neutral amine is available, leading to higher turnover. Together, these effects rationalize the pronounced maximum in catalytic signal at pH 8 and the loss of activity under acidic conditions.


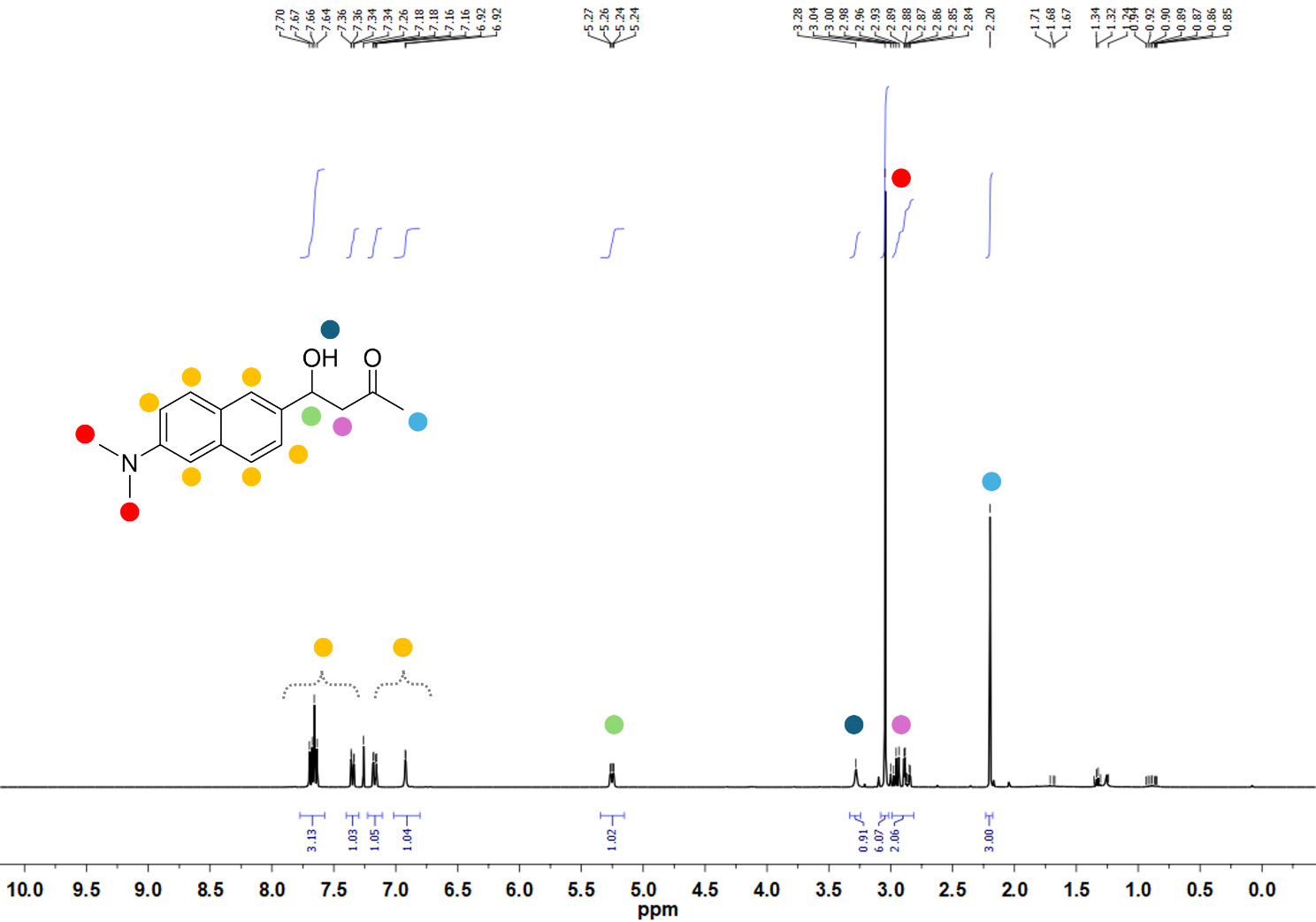


**Fig. S 29**. ^1^H NMR spectrum (400 MHz in CDCl_3_) of DANO.


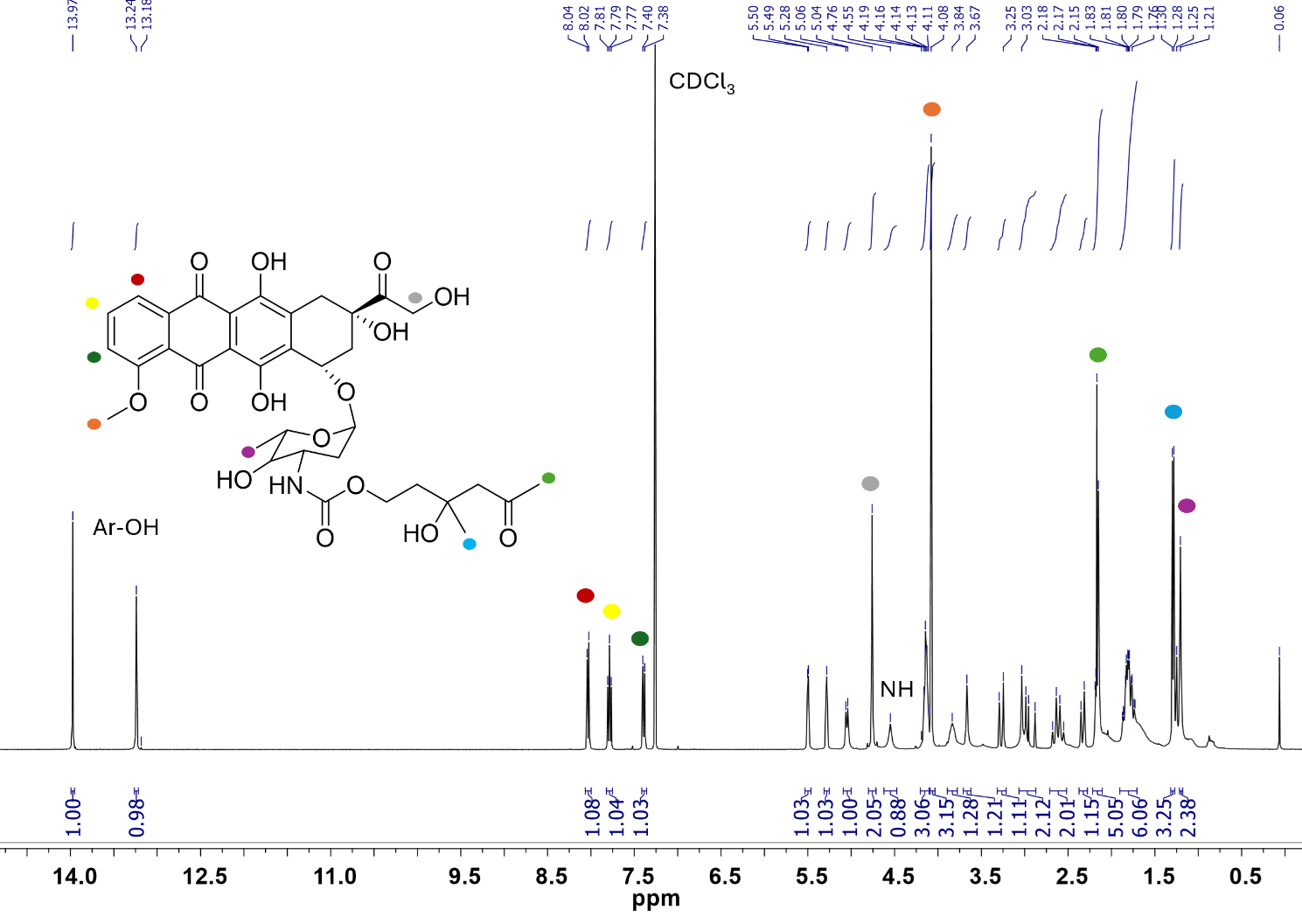


**Fig. S 30**. ^1^H NMR spectrum (400 MHz in CDCl_3_) of proDOX.


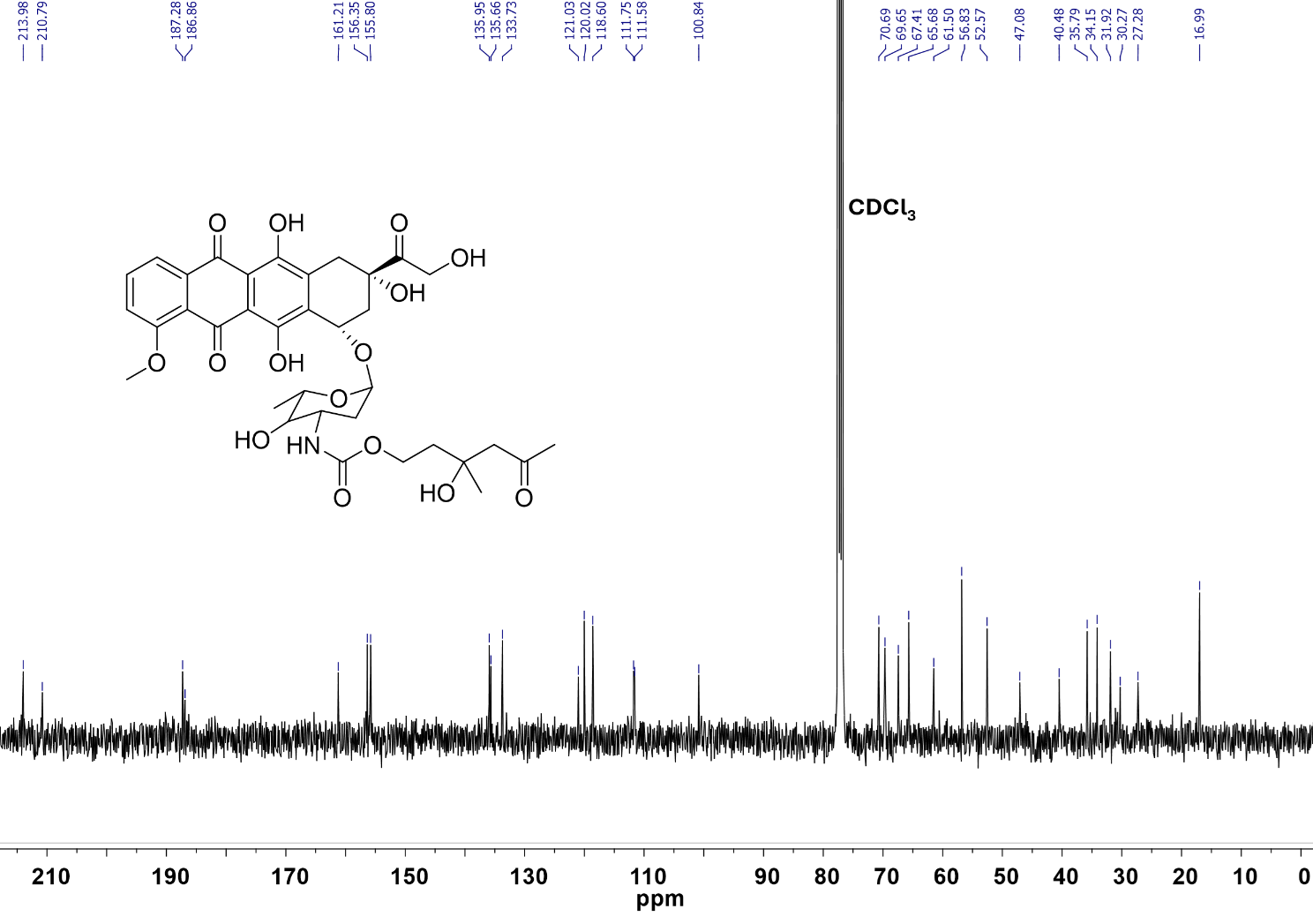


**Fig. S 31**. ^13^C NMR spectrum (100 MHz in CDCl_3_) of proDOX.


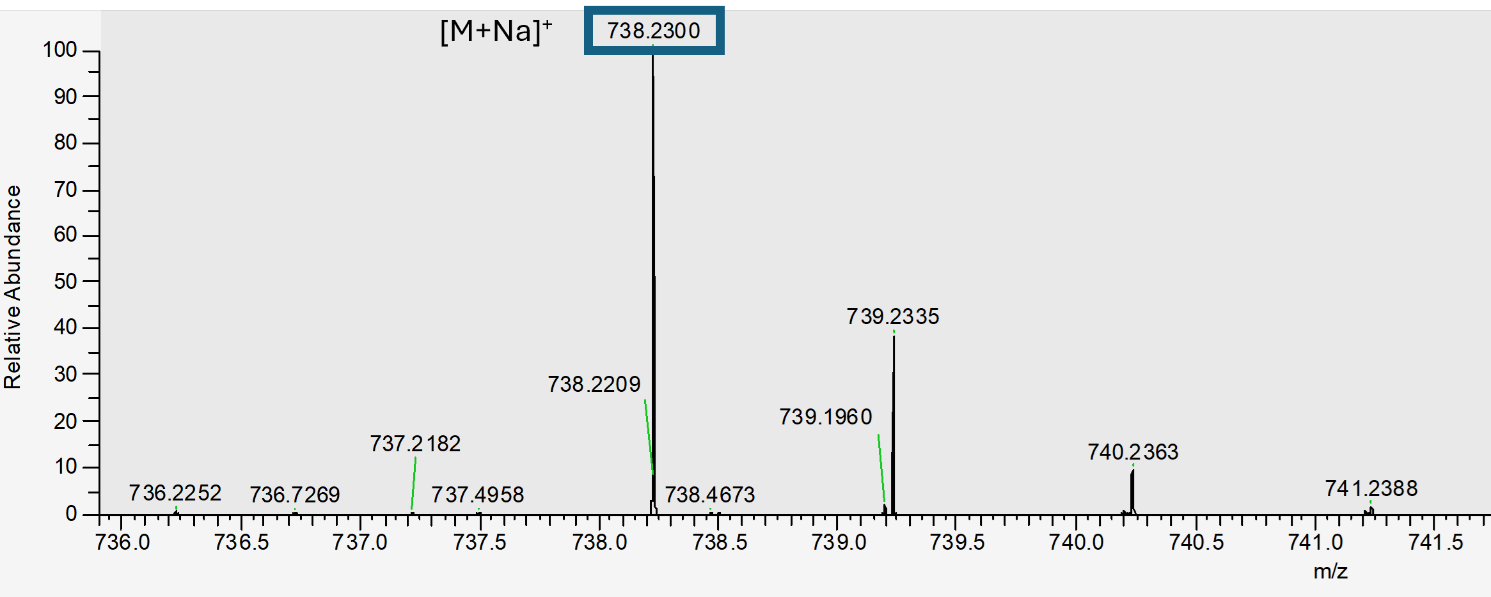


**Fig. S 32**. ESI(+)MS spectrum of proDOX.

## Supporting tables

Table S1. Comparison of examples of amine catalysis in water only, where no performance enhancers such as acids, polymers, micellization agents, or organic cosolvents have been added. What can be seen is that our TAPs perform excellently, considering that they do not require any cosolvents, micellar additives, or phase‑transfer agents. In addition, TAPs can also catalyze reactions with non‑water‑miscible ketones, which is why we have included these examples here as well.

|  | Catalyst system | Starting materials | Solvent | Loading in respect to aldehyde | *dr* ratio (anti/syn) | Yield | Ref |  |  |
| --- | --- | --- | --- | --- | --- | --- | --- | --- | --- |
| Non-water miscible ketone | | | | | | | | | |
| 1 | 1,1′-Binaphthyl-2,2′-diamine-based (S)-prolinamides^a^ | Cyclohexanone + 4-nitrobenzaldehyde | H_2_O, 35°C, 1.5 h | 10 mol% | n.a. | 15% (isolated) | ^14^ | |  |
| 2 | L-Proline^b^ | Cyclohexanone + p-nitrobenzaldehyde | H_2_O, 25°C, 96h | 10 mol% | n.a. | 0% | ^15^ | | |
| TAPs | Polyamine-silsesquioxane | Cyclopentanone + 4-nitrobenzaldehyde | H_2_O, 25°C, 12h | 6 mol% | 77:23 | 20% (isolated) | *This work* | | |
| Water miscible ketone | | | | | | | | | |
| 1 | Diamine functionalized silica particles | Acetone + p-nitrobenzaldehyde (partially water miscible system) | H_2_O, 55°C, 4h | ~2 mol% | n.a. | 20% conversion (not isolated; via HPLC) | ^16^ | | |
| 2 | Nornicotine^c^ | Acetone + p-nitrobenzaldehyde (partially water miscible system) | Phosphate buffer made with D_2_O, 25°C, 5h | 30 mol% | n.a. | 43% (not isolated; via NMR) | ^17^ | | |
| 3 | Nornicotine | Acetone + p-nitrobenzaldehyde (partially water miscible system) | Phosphate buffer containing 10% DMSO, 37°C, 10h | 30 mol% | n.a. | 81% | ^18^ | | |
| 4 | Proline-functionalized magnetic PMMA nanoparticles | Cyclohexanone + p-nitrobenzaldehyde | H_2_O, 25°C, up to 24h | 10 mol% | anti/syn >90:10 | Up to 90% (not isolated; via NMR) | ^19^ | | |
|  | Amino-group functionalized silica nanoparticles | Acetone + p-nitrobenzaldehyde | H_2_O, 60°C, 24h | 1 mol% | n.a. | Conversions up to 60% (no isolated yields reported) | ^20^ | | |

^a^Yields and *dr*, as well as *ee*, were massively improved using stearic acid as a micellization agent. ^b^When L-proline was used in DMSO, or functionalized with side chains that promote micellization, such as aliphatic chains, the yields improved drastically. ^c^The authors reported aldol catalysis promoted by the [Ru(bpy)_2_(nornicotine)_2_]^2+^ complex, where metal coordination to the pyridyl unit of nornicotine enhances its organocatalytic potential.

Table S2. Integration values normalized against the dioxetane area.


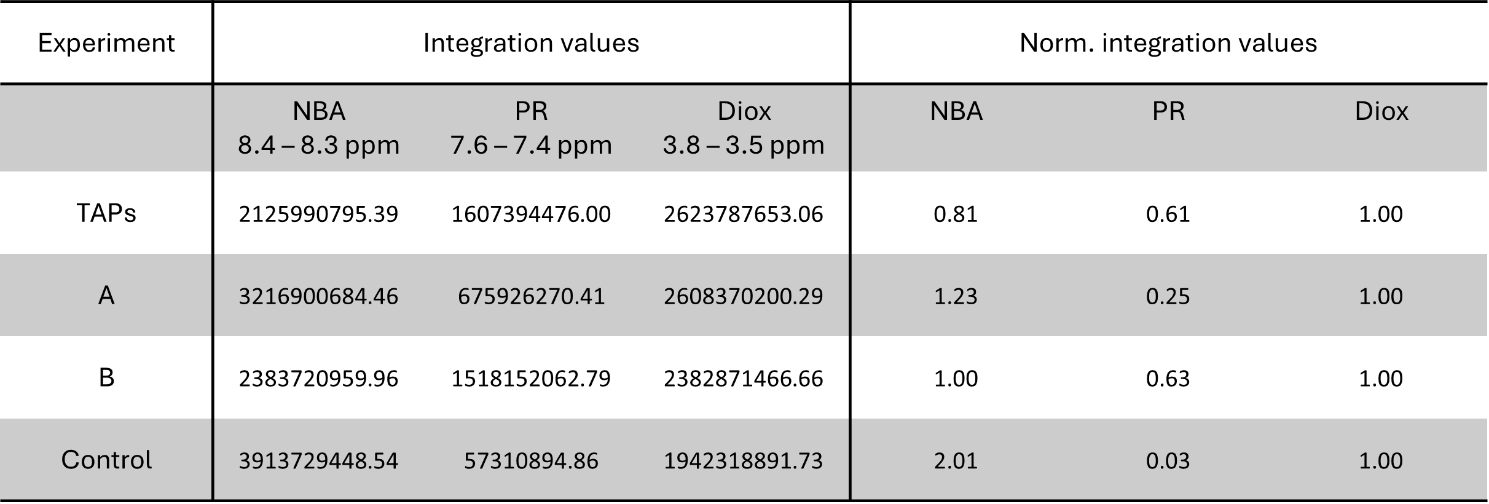


Table S3. Atomic coordinates of the optimized anti-intermediate truncated model in the TAPs catalyzed aldol reaction.

| Element | x | y | z |
| --- | --- | --- | --- |
| N | -4.89874100630914 | -0.70658915393470 | -2.96198458681408 |
| C | -5.91080724121670 | -1.49443425382098 | -2.25382961698010 |
| C | -5.33145359063069 | -2.79641644492705 | -1.72891288583638 |
| N | -4.26772080405667 | -2.64594940473761 | -0.73901275073515 |
| C | -4.74428165270080 | -2.29464771047389 | 0.58876645743953 |
| C | -3.59909434909779 | -1.97286094881369 | 1.53645611289988 |
| N | -2.89879289777470 | -0.76021432489818 | 1.09754815235993 |
| C | -1.79732749876849 | -0.82226930984306 | 0.43398013409382 |
| C | -1.15110452292174 | 0.31775924622640 | -0.27633116961788 |
| C | 0.30831538694348 | -0.12362543926418 | -0.36734982586880 |
| C | 0.22251694465067 | -1.63304180179895 | -0.58165727607957 |
| C | -0.97889139253065 | -2.06244263345325 | 0.26071985731505 |
| C | -3.56054507401048 | 0.49806836865103 | 1.47410016930704 |
| C | -3.24123889669292 | 0.87753835419109 | 2.91301299694876 |
| C | -1.79842048748558 | 1.33214386285146 | 3.14226473490971 |
| Si | -1.38053526850668 | 3.01245093921264 | 2.50506918549723 |
| O | -1.31859855345061 | 4.14400523026904 | 3.70182478231530 |
| C | -0.96044893176703 | 5.43144810455696 | 3.16988065809621 |
| O | -2.47078643077399 | 3.58664184777210 | 1.40396339404275 |
| C | -2.13218576618624 | 4.92854516439010 | 1.00983134536912 |
| C | -0.81436504928927 | 5.37282909602019 | 1.64829615745979 |
| C | 0.32229942272073 | 4.42377134339830 | 1.25738640862490 |
| O | 0.07880487861369 | 3.08945462864271 | 1.74021934170680 |
| H | -5.34196635603809 | 0.11717032565545 | -3.37042826001967 |
| H | -4.51712336586810 | -1.24613020258496 | -3.73123053209825 |
| H | -6.29620405460706 | -0.88687746626911 | -1.43130568168724 |
| H | -6.76180792434080 | -1.73663783089850 | -2.90025103762053 |
| H | -4.92845148453879 | -3.36614060037320 | -2.57236896469458 |
| H | -6.12827938805498 | -3.40008499207867 | -1.28942201817543 |
| H | -5.45583654239075 | -1.46016561470551 | 0.57818519678713 |
| H | -5.27132824190915 | -3.15528358167834 | 1.00760373691242 |
| H | -2.89431621920093 | -2.79799975349860 | 1.59239834692717 |
| H | -3.99547658502803 | -1.79179122101950 | 2.53381153094982 |
| H | 0.80355751579243 | 0.09906787033250 | 0.58031725250683 |
| H | 0.85017004630712 | 0.39750683018399 | -1.15545739444253 |
| H | 1.13130060956748 | -2.15141136433155 | -0.28120339558160 |
| H | 0.04609159645282 | -1.86347320672818 | -1.63335866093285 |
| H | -0.66780277585132 | -2.36774614146716 | 1.26721466775476 |
| H | -1.53924588399248 | -2.89956159707928 | -0.15660841479257 |
| H | -4.63146026879561 | 0.33980679766707 | 1.35782585998604 |
| H | -3.27225080363506 | 1.27604999263032 | 0.77805243344096 |
| H | -3.46822023018041 | 0.03843345547644 | 3.57433446332914 |
| H | -3.94196128311380 | 1.67048492246921 | 3.18446812687975 |
| H | -1.08618609412048 | 0.60449383044674 | 2.74131188703171 |
| H | -1.59805979939799 | 1.36281202165799 | 4.21773358095406 |
| H | -0.01946150900842 | 5.74733444945955 | 3.62801912175998 |
| H | -1.73987097753953 | 6.14639186037622 | 3.44505951981662 |
| H | -2.94249718279764 | 5.59512114401287 | 1.31636381359440 |
| H | -2.05413826517260 | 4.96128291503349 | -0.07951102990746 |
| H | -0.57116942822114 | 6.37348582651504 | 1.28215440933042 |
| H | 1.26505179544274 | 4.77551229319038 | 1.68340634149642 |
| H | 0.42447368376911 | 4.38463908705558 | 0.16985148284631 |
| H | -1.27650172923039 | 1.27776995558105 | 0.21882303499186 |
| C | -1.83457478091237 | 0.40877254898545 | -1.67535931466462 |
| O | -3.19651709033342 | 0.68704549041482 | -1.50951964500497 |
| C | -1.15100790369900 | 1.46972672199246 | -2.50130576623286 |
| C | -0.48657559128038 | 1.13195169595448 | -3.67412288121089 |
| C | 0.13301326816009 | 2.10531453180936 | -4.43901750082276 |
| C | 0.07409467258254 | 3.41931557822710 | -4.00903970991932 |
| C | -0.58217329029640 | 3.78616712199438 | -2.84375627743657 |
| C | -1.19219259402518 | 2.80076607342896 | -2.09469446127018 |
| N | 0.72475944976690 | 4.45190343056599 | -4.80560188691073 |
| O | 0.66496770620713 | 5.59995306586978 | -4.41199396050695 |
| O | 1.29585523808836 | 4.11515406380517 | -5.82373792355123 |
| H | -0.45290254491222 | 0.09754504845468 | -3.99481718980802 |
| H | 0.65229279066462 | 1.85393163027147 | -5.35262989213522 |
| H | -0.60726827181134 | 4.82122169559718 | -2.53420968991484 |
| H | -1.70507507292560 | 3.07023805995225 | -1.18004832823729 |
| H | -1.71380473666329 | -0.55074667823615 | -2.18939179116986 |
| H | -3.64160674898351 | -1.92667204998474 | -1.08173351212880 |
| H | -3.78086039156047 | 0.08722317056209 | -2.09708427609442 |
| O | -4.76107953405985 | 2.94116490130889 | -1.52686475599847 |
| H | -4.08202539047995 | 2.24002461408981 | -1.49003598774072 |
| H | -4.28461994718221 | 3.75344233281255 | -1.71351107845024 |
| O | -2.80858255714988 | -5.01754552211262 | -0.71914199880743 |
| H | -3.34176405060338 | -4.18573314545106 | -0.69375224207646 |
| H | -2.82106569654869 | -5.34466196430872 | 0.18253828500846 |
| O | -6.21331207602282 | 1.94512657664696 | -3.65472996458674 |
| H | -5.88169991846066 | 2.33690552468739 | -4.46546743937418 |
| H | -5.71377101061449 | 2.38481071741386 | -2.93848200475140 |

Table S4. Atomic coordinates of the optimized anti-intermediate truncated model in the TAPs catalyzed aldol reaction.

| Element | x | y | z |
| --- | --- | --- | --- |
| N | -5.91254686966451 | -0.70397442125442 | -1.83820026442712 |
| C | -6.53002768891175 | -1.87761345389037 | -1.23334114570025 |
| C | -5.49102605463504 | -2.97375784685108 | -1.03125264730661 |
| N | -4.29160530467809 | -2.53563622865066 | -0.32261552765232 |
| C | -4.54130126456208 | -2.15354632845516 | 1.05762115190708 |
| C | -3.27721885568368 | -1.64781005731843 | 1.72901136043453 |
| N | -2.70061222317098 | -0.51581038012985 | 0.99046178857302 |
| C | -1.51650774407752 | -0.57791775699769 | 0.48625642627026 |
| C | -0.91623860456914 | 0.45819405085370 | -0.41647378779662 |
| C | 0.58357294377625 | 0.15826669475410 | -0.36108277203403 |
| C | 0.70266714065210 | -1.33765899795439 | -0.06128826945887 |
| C | -0.55921863289873 | -1.69374877055611 | 0.72533737595885 |
| C | -3.58883530924161 | 0.64638154097780 | 0.81994909770092 |
| C | -4.14669533191496 | 1.16826007922623 | 2.13286770340325 |
| C | -3.09508734409762 | 1.60132218388203 | 3.15456786801162 |
| Si | -1.95741173800577 | 2.95118558607441 | 2.62487477652250 |
| O | -1.60138034228569 | 4.02196150216481 | 3.82446020159140 |
| C | -0.66405639477674 | 5.00904069185126 | 3.35493088513104 |
| O | -2.55823206980171 | 3.85115996973340 | 1.37849813647756 |
| C | -1.65245939502385 | 4.91854669159795 | 1.04459321199585 |
| C | -0.36439788826284 | 4.82329390384161 | 1.86509618869915 |
| C | 0.32086118499446 | 3.47656526876489 | 1.62312600413295 |
| O | -0.49986865684819 | 2.39020140259497 | 2.08806419213832 |
| H | -6.54803156035078 | 0.08227632411656 | -1.81127220750034 |
| H | -5.71103806029398 | -0.87573208007864 | -2.81587209965725 |
| H | -6.95400758726661 | -1.57840382508506 | -0.27232602869525 |
| H | -7.35600110807679 | -2.29575506125656 | -1.82344291712206 |
| H | -5.17146252689150 | -3.34737283747939 | -2.00889467756600 |
| H | -5.93580533732854 | -3.81421267387759 | -0.49304692338835 |
| H | -5.33756211540400 | -1.40756762177028 | 1.15684404871584 |
| H | -4.86592174940225 | -3.03513197881957 | 1.61518138129196 |
| H | -2.53563249977894 | -2.43668130576757 | 1.78289151476018 |
| H | -3.50100038481623 | -1.32066549528902 | 2.74392235010241 |
| H | 1.01551225882962 | 0.73835326192729 | 0.45493617556178 |
| H | 1.09589879088300 | 0.45424847869484 | -1.27734067569892 |
| H | 1.60266258422900 | -1.56517265175214 | 0.50767799753764 |
| H | 0.75137488836954 | -1.92829990113031 | -0.97651390046669 |
| H | -0.36942297593068 | -1.74263869139268 | 1.80300578146725 |
| H | -0.99043165601395 | -2.65556754462923 | 0.43405606613719 |
| H | -4.40187716823076 | 0.34843529040798 | 0.15299879210054 |
| H | -3.03134869645429 | 1.43217974861019 | 0.32005113615582 |
| H | -4.81413217262894 | 0.43080718791962 | 2.58254475489766 |
| H | -4.77856497369642 | 2.01711048886651 | 1.86295907711985 |
| H | -2.47788991286281 | 0.75307225201692 | 3.46897459271654 |
| H | -3.60171751117860 | 1.94878980153314 | 4.05957264183976 |
| H | 0.25670396611569 | 4.91901926605954 | 3.93728067849936 |
| H | -1.09364305841737 | 5.99802124076275 | 3.53131774597196 |
| H | -2.15275325393557 | 5.87182694935663 | 1.23457984581937 |
| H | -1.42277708281092 | 4.85299767856134 | -0.02142605904270 |
| H | 0.31154985354666 | 5.62037757347726 | 1.54525448771516 |
| H | 1.27380734863975 | 3.43679015999319 | 2.15589126392332 |
| H | 0.51170807705057 | 3.33677331776896 | 0.55478278919177 |
| H | -1.12407872846400 | 1.46298796174506 | -0.05572033538892 |
| C | -1.55101649859217 | 0.32946150949426 | -1.82481153291938 |
| H | -2.63242223873841 | 0.45105273382457 | -1.72234869911066 |
| C | -1.06123415470846 | 1.43151442010711 | -2.73067807331215 |
| C | -0.31165358659365 | 1.16882944893156 | -3.87028769751967 |
| C | 0.12267385412957 | 2.20393609990307 | -4.68106858605317 |
| C | -0.21024744823346 | 3.50107090141690 | -4.33789403904568 |
| C | -0.96925128203515 | 3.79273853393656 | -3.21475446127374 |
| C | -1.39145491923967 | 2.74973175278525 | -2.41725280108155 |
| N | 0.24386677175245 | 4.59498833991219 | -5.18657214783129 |
| O | -0.06717563186324 | 5.72725491048319 | -4.87431592435787 |
| O | 0.91163125264018 | 4.32195723686940 | -6.16411121561366 |
| H | -0.06504220298738 | 0.15282373842584 | -4.14857977798859 |
| H | 0.70890050228994 | 2.00822717385596 | -5.56732802669848 |
| H | -1.22338755595769 | 4.81599840935134 | -2.97854995106971 |
| H | -1.99654193685846 | 2.96474192246965 | -1.54468963094239 |
| O | -1.39127183485807 | -0.96605280553546 | -2.35674786926190 |
| H | -3.95895468349580 | -1.72271885379138 | -0.83236870218655 |
| H | -0.46489141948015 | -1.15389146496686 | -2.53453026032707 |
| O | -3.23380950661169 | -1.56523092321295 | -4.34595053223913 |
| H | -2.57230455970654 | -1.45660201313297 | -3.64112974033335 |
| H | -3.85522498711584 | -2.21732762412792 | -4.01306303503458 |
| O | -4.22416441110812 | 1.03888168136287 | -4.56525795294022 |
| H | -3.88577185969958 | 0.12683306002955 | -4.50523680354747 |
| H | -3.45454009988201 | 1.57145094248773 | -4.77573518987938 |
| O | -2.24041699371894 | -4.38913327793425 | -0.63673594576661 |
| H | -1.84965335391605 | -4.18422617565420 | -1.48863870856558 |
| H | -2.97367241915245 | -3.73238031504078 | -0.53064194667136 |

## Supporting references

1. Schmidt, J.; Ehasz, C.; Epperson, M.; Klas, K.; Wyatt, J.; Hennig, M.; Forconi, M., The effect of the hydrophobic environment on the retro-aldol reaction: comparison to a computationally-designed enzyme. *Org. Biomol. Chem.* **2013,** *11* (48), 8419-8425.

2. List, B.; Barbas, C. F.; A., L. R., Aldol sensors for the rapid generation of tunable fluorescence byantibody catalysis. *Proc. Natl. Acad. Sci. U.S.A.* **1998,** *95* (26), 15351–15355.

3. Shabat, D.; Rader, C.; List, B.; Lerner, R. A.; Barbas, C. F., Multiple event activation of a generic prodrug trigger byantibody catalysis. *Proc. Natl. Acad. Sci.* **1999,** *96* (12), 6925-6930.

4. Shamis, M.; Lode, H. N.; Shabat, D., Bioactivation of Self-Immolative Dendritic Prodrugs by Catalytic Antibody 38C2. *J. Am. Chem. Soc.* **2004,** *126* (6), 1726-1731.

5. Neese, F., Software Update: The ORCA Program System—Version 6.0. *Wiley Interdiscip. Rev. Comput. Mol. Sci.* **2025,** *15* (2), e70019.

6. de Souza, B., GOAT: A Global Optimization Algorithm for Molecules and Atomic Clusters. *Angew. Chem. Int. Ed.* **2025,** *64* (18), e202500393.

7. Bannwarth, C.; Ehlert, S.; Grimme, S., GFN2-xTB-An Accurate and Broadly Parametrized Self-Consistent Tight-Binding Quantum Chemical Method with Multipole Electrostatics and Density-Dependent Dispersion Contributions. *J. Chem. Theory Comput.* **2019,** *15* (3), 1652-1671.

8. Caldeweyher, E.; Ehlert, S.; Hansen, A.; Neugebauer, H.; Spicher, S.; Bannwarth, C.; Grimme, S., A generally applicable atomic-charge dependent London dispersion correction. *J. Chem. Phys.* **2019,** *150*, 154122.

9. Weigend, F.; Ahlrichs, R., Balanced basis sets of split valence, triple zeta valence and quadruple zeta valence quality for H to Rn: Design and assessment of accuracy. *Phys. Chem. Chem. Phys.* **2005,** *7* (18), 3297-305.

10. de Andrade, K. N.; Peixoto, B. P.; Carneiro, J. W. M.; Fiorot, R. G., Exploring borderline S(N)1-S(N)2 mechanisms: the role of explicit solvation protocols in the DFT investigation of isopropyl chloride. *RSC Adv.* **2024,** *14* (7), 4692-4701.

11. Contreras-Garcia, J.; Johnson, E. R.; Keinan, S.; Chaudret, R.; Piquemal, J. P.; Beratan, D. N.; Yang, W., NCIPLOT: a program for plotting non-covalent interaction regions. *J. Chem. Theory. Comput.* **2011,** *7* (3), 625-632.

12. Johnson, E. R.; Keinan, S.; Mori-Sánchez, P.; Contreras-García, J.; Cohen, A. J.; Yang, W., Revealing Noncovalent Interactions. *J. Am. Chem. Soc.* **2010,** *132* (18), 6498-6506.

13. Humphrey, W.; A., D.; Schulten, K., VMD: Visual Molecular Dynamics. *J. Mol. Graph.* **1996,** *14*, 33-38.

14. Guizzetti, S.; Benaglia, M.; Raimondi, L.; Celentano, G., Enantioselective Direct Aldol Reaction “on Water” Promoted by Chiral Organic Catalysts. *Org. Lett.* **2007,** *9* (7), 1247-1250.

15. Mase, N.; Nakai, Y.; Ohara, N.; Yoda, H.; Takabe, K.; Tanaka, F.; Barbas, C. F., Organocatalytic Direct Asymmetric Aldol Reactions in Water. *J. Am. Chem. Soc.* **2006,** *128* (3), 734-735.

16. De Vylder, A.; Lauwaert, J.; De Clercq, J.; Van Der Voort, P.; Jones, C. W.; Thybaut, J. W., Aminated poly(ethylene glycol) methacrylate resins as stable heterogeneous catalysts for the aldol reaction in water. *J. Catal.* **2020,** *381*, 540-546.

17. Guzman Rios, D.; Romero, M. A.; Gonzalez-Delgado, J. A.; Arteaga, J. F.; Pischel, U., Metal-Mediated Organocatalysis in Water: Serendipitous Discovery of Aldol Reaction Catalyzed by the [Ru(bpy)(2)(nornicotine)(2)](2+) Complex. *J. Org. Chem.* **2022,** *87* (8), 5412-5418.

18. Dickerson, T. J.; Janda, K. D., Aqueous Aldol Catalysis by a Nicotine Metabolite. *J. Am. Chem. Soc.* **2002,** *124* (13), 3220-3221.

19. Alvarez-Bermudez, O.; Landfester, K.; Zhang, K. A. I.; Munoz-Espi, R., Proline-Functionalized Magnetic Nanoparticles as Highly Performing Asymmetric Catalysts. *Macromol. Rapid Commun.* **2024,** *45* (24), e2400615.

20. Singappuli-Arachchige, D.; Kobayashi, T.; Wang, Z.; Burkhow, S. J.; Smith, E. A.; Pruski, M.; Slowing, I. I., Interfacial Control of Catalytic Activity in the Aldol Condensation: Combining the Effects of Hydrophobic Environments and Water. *ACS Catal.* **2019,** *9* (6), 5574-5582.
